# Alterations in infant gut microbiome composition and metabolism after exposure to glyphosate and Roundup and/or a spore-based formulation using the SHIME technology

**DOI:** 10.1017/gmb.2022.5

**Published:** 2022-07-26

**Authors:** Robin Mesnage, Marta Calatayud, Cindy Duysburgh, Massimo Marzorati, Michael N. Antoniou

**Affiliations:** 1 Gene Expression and Therapy Group, King’s College London, Faculty of Life Sciences & Medicine, Department of Medical and Molecular Genetics, Guy’s Hospital, London, SE1 9RT, UK.; 2 ProDigest BV, Ghent, Belgium; 3 Center for Microbial Ecology and Technology, Faculty of Bioscience Engineering, Department of Biotechnology, Ghent University, Ghent, Belgium

**Keywords:** pesticides, probiotics, metabolomics, SHIME, glyphosate, gut microbiota

## Abstract

Despite extensive research into the toxicology of the herbicide glyphosate, there are still major unknowns regarding its effects on the human gut microbiome. We describe the effects of glyphosate and a Roundup glyphosate-based herbicide on infant gut microbiota using SHIME technology. SHIME microbiota culture was undertaken in the presence of a concentration of 100-mg/L glyphosate and the same glyphosate equivalent concentration of Roundup. Roundup and to a lesser extent glyphosate caused an increase in fermentation activity, resulting in acidification of the microbial environment. This was also reflected by an increase in lactate and acetate production concomitant to a decrease in the levels of propionate, valerate, caproate and butyrate. Ammonium production reflecting proteolytic activities was increased by Roundup exposure. Global metabolomics revealed large-scale disturbances, including an increased abundance of long-chain polyunsaturated fatty acids. Changes in bacterial composition measured by qPCR and 16S rRNA suggested that lactobacilli had their growth stimulated as a result of microenvironment acidification. Co-treatment with the spore-based probiotic formulation MegaSporeBiotic reverted some of the changes in short-chain fatty acid levels. Altogether, our results suggest that glyphosate can exert effects on human gut microbiota.

## Introduction

Glyphosate is globally the most widely used broad-spectrum herbicide and crop desiccant (Maggi et al., 2019) being an active ingredient in many herbicide formulations, including Roundup. In addition to glyphosate salts, formulations of glyphosate-based herbicides (GBHs) contain some co-formulants such as surfactants, which vary in nature and concentration between different commercial products (Mesnage et al., 2019). Although GBHs have been approved by regulatory bodies worldwide, concerns about their effects on humans and the environment persist (Robinson et al., 2020).

Glyphosate principally acts by inhibiting the activity of 5-enolpyruvylshikimate-3-phosphate synthase (EPSPS) of the shikimate pathway (Boocock and Coggins, 1983), causing a shortage in aromatic amino acid biosynthesis in plants. The shikimate pathway is also found in microorganisms, including those inhabiting the human gut microbiome (Mesnage and Antoniou, 2020). Consequently, concerns have been raised about the ability of glyphosate and GBHs to have adverse effects through its interactions with the community of bacteria residing in the digestive tract. Likely, human exposure to glyphosate or GBH, mainly occurring via oral route due to direct exposure in occupational settings or environmental exposure through residues in food (Faniband et al., 2021), reaches the colonic environment due to a low absorption in the gastrointestinal tract (Anadón et al., 2009; Faniband et al., 2021).

We have recently described how glyphosate and a European Union Roundup GBH (MON 52276) can indeed inhibit EPSPS of the shikimate pathway in the gut microbiome of rats (Mesnage et al., 2021b). In addition, *Shinella zoogleoides* was increased by exposure to MON 52276 but not glyphosate (Mesnage et al., 2021b), suggesting a significant role of additives in GBH effects. However, the gut microbiota of laboratory rodents or wild animals is substantially different from the human gut microbiota and knowledge gaps remain as to whether glyphosate also affects human gut microbiota.

Especially relevant is the study of potential health effects and risk assessment in sensitive populations like infants. Infants are especially sensitive to xenobiotic exposures due to immaturity of some metabolic functions, larger surface area (relative to body weight), the high sensitivity of target organs (windows of susceptibility) and cumulative effects due to longer exposure times, putting them at a higher level of risk compared to adults (Calatayud Arroyo et al., 2021). The process of colonization and development of gut microbiota in early life has been previously linked to adult diseases, including immune and metabolic disorders, such as asthma, eczema, inflammatory bowel disease, type 1 diabetes or obesity (Zhuang et al., 2019), and our understanding on how environmental xenobiotic exposures might influence these processes is still in its infancy.

To cover this gap of knowledge, we used the *in vitro* SHIME technology mimicking the entire gastrointestinal, including the mucosal and luminal compartment (Van den Abbeele et al., 2013; Venema and van den Abbeele, 2013), and applied metagenomics and metabolomics approaches in a long-term (3 weeks), repeated dose setup. The first aim of the study was to assess the impact of glyphosate and a GBH on the activity and composition of microbiota obtained from a 3-year-old human donor. The effect of glyphosate alone versus glyphosate formulated product (Roundup MON 76207) was also evaluated. In a second stage, we assessed the efficacy of a spore-based formulation, MegaSporeBiotic (Microbiome Labs; https://microbiomelabs.com/home/), in remediating the effects of glyphosate on gut microbiota. To the best of our knowledge, no study has been performed to date to determine if the effects of glyphosate and GBHs on the gut microbiome can also be mitigated through the use of pre- and probiotics. Probiotics have been traditionally used to modulate gut microbial communities and provide benefit to the host via different mechanisms (Astolfi et al., 2019). Some probiotic strains have also been proved to reduce the toxicity of xenobiotics by modulating oxidative stress, or xenobiotics adsorption capacity, with a focus on inorganic compounds of Pb, Cd, As and Hg (de Matuoka e Chiocchetti et al., 2020; Majlesi et al., 2017).

Our results can increase the understanding of how glyphosate or GBH can affect human health in sensitive populations through modulation of the gut microbiota and how we can potentially promote a microbial equilibrium through probiotic supplementation.

## Material and methods

### Chemicals

The test products were glyphosate certified reference material, 1,000 μg/mL in H_2_O (EPA 547 Glyphosate Solution, Merck KGaA, Darmstadt, Germany) and the glyphosate containing herbicide formulation MON 76207 (Roundup PROMAX on the U.S. market, EPA Registration No. 524-579). The daily dose of glyphosate was selected during a pre-screening experiment made to evaluate changes in microbial activity and *Enterobacteriaceae* levels, and set at a concentration of 100 mg/L. Roundup was tested at a similar glyphosate level, taking into account that Roundup consisted for 48.7 per cent of glyphosate. During the co-treatment period, the spore-based probiotic, MegaSporeBiotic (Microbiome Labs Physicians Exclusive, LLC., St. Augustine, FL, USA), was additionally administered at a concentration of 340 mg containing 4 billion colony-forming units (CFU), corresponding to a human ingestion of 2 capsules/day. MegaSporeBiotic contains five different Bacillus strains, ie., *Bacillus indicus HU36, Bacillus clausii* (*SC-109*)*, Bacillus subtilis HU58, Bacillus licheniformis* (SL-307) and *Bacillus coagulans* (*SC-208*).

### Fecal sample

An infant donor was selected based on the following inclusion criteria: healthy, age between 12 and 36 months, no antibiotics or any other drug intake at least during the previous 6 months, normal weight, no constipation, hospital-born, no known diseases at least during the previous 6 months and exclusive breastfeeding for at least 3 months. The fecal sample was obtained in a plastic container with an “Oxoid AnaeroGen” bag to limit the sample’s exposure to oxygen, immediately transported to the lab and used to inoculate the M-SHIME system. Briefly, a mixture of 1:10 (w/v) of fecal sample and anaerobic phosphate buffer (K_2_HPO_4_ 8.8 g/L; KH_2_PO_4_ 6.8 g/L; sodium thioglycolate 0.1 g/L; sodium dithionite 0.015 g/L; N2-flushed for 15 min) was homogenized for 10 min (BagMixer 400, Interscience, Louvain-La-Neuve, Belgium). After centrifugation (500 g, 2 min; Centrifuge 5417C, Eppendorf, VWR, Belgium) to remove large particles, the fecal sample was used to inoculate different M-SHIME reactors.

### Short-term colonic incubation

Short-term colonic incubations were performed to determine the concentration of glyphosate to be administered in the long-term SHIME experiment. The short-term screening assay tested a concentration range of glyphosate (0, 0.5, 1, 3, 10, 100 and 1,000 mg/L) on a simulated proximal colonic environment (pH 6.2–6.4) with a representative bacterial inoculum. This bacterial inoculum was obtained from the long-term M-SHIME reactor during the stabilization period and simulated the colonic microbiota of a 3-year-old infant.

Briefly, short-term colonic incubations were performed by inoculating a 10 per cent (v/v) of a homogeneous mixture of the ascending, descending and transverse colon M-SHIME luminal fluid (stabilization phase) into colonic media (K_2_HPO_4_ 5.7 g/L; KH_2_PO_4_ 17.9 g/L; NaHCO_3_; 2.2 g/L; yeast extract 2.2 g/L; peptone 2.2 g/L; mucin 1.1 g/L; cysteine 0.5 g/L; Tween80 2.2 mL/L). Details on the M-SHIME inoculum are described in the next section. Incubations containing different glyphosate concentrations were performed for 48 h at 37°C, under shaking (90 rpm) and anaerobic conditions. Short-term effects of different glyphosate concentrations on microbial activity markers [gas production, acid/base consumption, lactate, ammonia, short-chain fatty acids (SCFAs) and branched short-chain fatty acids (b-SCFAs)] and qPCR measurement of *Enterobacteriaceae* were assessed at time points 0, 6, 24 and 48 h. Since the production of microbial metabolites in the colon reactors alters the pH, the pH is controlled through the addition of acid or base (ie., base consumption).

### Long-term colonic incubation

The reactor setup of the M-SHIME, representing the gastrointestinal tract of the human infant, was conducted as first described by Molly et al. (1993), with modifications as defined by Van den Abbeele et al. (2012, 2013, 2021).

The M-SHIME included both the luminal and mucosal microbiota, and consisted of three colonic vessels simulating the ascending, transverse and descending colon. Anaerobic conditions were achieved by 15-min flushing of all reactors with N2, 15 min twice per day. Double jacketed reactors connected to a recirculating warm water bath kept the M-SHIME units at 37ºC, and continuous stirring was maintained throughout the duration of the experiment. All reactors incorporated a mucosal environment in the luminal suspension. The mucosal environment consisted of 80 mucin agar-covered microcosms (AnoxKaldnes K1 carrier; AnoxKaldnes AB, Lund, Sweden) per vessel, prepared and submerged in defined colonic medium as previously described (Calatayud et al., 2021).

Each M-SHIME reactor was inoculated with 20 per cent (v/w) infant fecal inoculum. During the first 16 h, reactors were operated in batch mode to allow for initial stabilization of the system and colonization of the mucus microenvironment. Subsequently, the stabilization period (2 weeks, day-14 to day-1; Supplementary Figure S1) started in semi-continuous mode. Each experimental M-SHIME unit consisted of a first reactor that simulated over time the stomach and small intestine and that operated according to a fill-and-draw principle, with peristaltic pumps adding a defined amount of nutritional colonic medium to the stomach (gastric reactor = 140 mL; pH 3; 30 min), followed by the addition of 60 mL of simulated pancreatic and bile juice (NaHCO_3_ 2.6 g/L, Oxgall 4.8 g/L and pancreatin 1.9 g/L; small intestinal reactor = 200 mL; pH 6.5; 105 min). After this time, the intestinal suspension was pumped to the ascending colon vessel (AC reactor = 500 mL; pH 5.7–5.9; 250 min), and sequentially to the transverse (TC reactor = 800 mL; pH 6.2–6.4; 260 min) and descending colon (DC reactor = 600 mL; pH 6.6–6.9; 280 min) vessels. The pH was automatically adjusted by continuous measurement (Senseline pH meter F410; ProSense, Oosterhout, The Netherlands) coupled to a pH controller (Consort R301) and automatic pump (Master Flex 109 pump drive; Cole-Parmer Instrument Company, LLC), with dosing of either HCl (0.5 M) or NaOH (0.5 M; Carl Roth, Belgium), as required.

**Figure 1:**
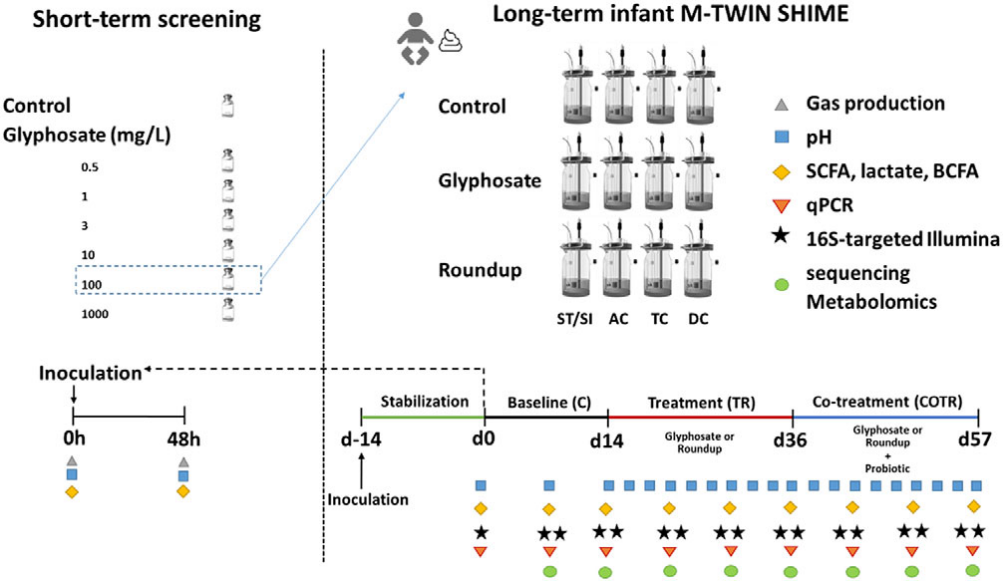
Experimental design. TWINSHIME was used in order to evaluate the impact of glyphosate and a Roundup glyphosate-based herbicide formulation on the microbiota of a healthy human 3-year-old donor. The effect of adding MegaSporeBiotic spore-based probiotic. Culture period was for 10 weeks in chambers simulating the ascending (AC), transverse (TC) and descending colon (DC). The index highlights the molecular measurements undertaken to assess the effects of the different treatments at various sampling timepoints (lower right panel).

After the stabilization period, a control period (C) of 2 weeks (d0–d14) was used as a baseline for microbial community composition and activity (Figure 1). During the control period, samples were obtained three times per week from the luminal environment to evaluate microbial activity (measurement of lactate, ammonia, SCFAs and b-SCFAs), and once per week for community composition employing qPCR for detection of Firmicutes and Bacteroidetes phyla, *Lactobacillus* spp., *Bifidobacterium* spp., *Akkermansia muciniphila* and *Faecalibacterium prausnitzii* and 16S rRNA Illumina sequencing. Community composition of the mucosal environment was also analysed in samples obtained once per week from the mucus beads.

Subsequently, during the treatment period (TR), a concentration of 100-mg/L glyphosate as pure standard or Roundup herbicide formulation at the same glyphosate equivalent concentration was added to the colonic nutritional media, and administered to the stomach reactor three times per day over 3 weeks, corresponding to a dose of glyphosate of 14 mg to be distributed over the SHIME unit (AC, TC and DC; total volume = 1.9 L). Due to the dynamic nature of the system following a fill-and-draw principle, a steady-state equilibrium was expected after the first 72 h of the assay. After the TR period, supplementation with the MegaSporeBiotic spore-based probiotic (4 billion CFUs) was administered on top of the nutritional media and glyphosate (COTR) for three more weeks (Figure 1).

During the TR and COTR periods, samples were obtained three times per week for assessing microbial metabolic activity and once per week for microbial community composition, as previously described (Figure 1). Additionally, luminal samples for metabolomics were obtained once per week during the C, TR and COTR periods.

### Microbial community activity

Metabolic activity of the gut microbial communities was evaluated by quantifying general markers of fermentation (pH and gas production), lactate, ammonia, SCFA and b-SCFA production during the short-term experiment at 0, 6, 24 and 48 h.

During the long-term M-TWIN SHIME assay, the same markers were quantified three times per week, with the exception of gas production.

The pH was recorded using an automatic probe (Senseline F410; ProSense, Oosterhout, The Netherlands). Gas production was estimated by measuring bioreactor pressure with a handheld pressure indicator (CPH6200; Wika, Echt, The Netherlands). Lactate quantification was performed using a commercial enzyme-based assay kit (R-Biopharm, Darmstadt, Germany) according to the manufacturer’s instructions. SCFA and b-SCFA measurements were conducted as previously described (De Weirdt et al., 2010). Ammonia was quantified by the Kjeldahl nitrogen determination method again as previously described (Chaikham et al., 2012).

### DNA extraction

Total genome DNA from pelleted bacterial cells obtained from a 1-mL sample or 0.25-g mucin agar collected from the mucus beads, was extracted as previously described (Boon et al., 2003) with modifications as described by Boon et al. (2003) and Duysburgh et al. (2019). DNA concentration and purity was monitored by electrophoresis on 2 per cent (w/v) agarose gels and spectrophotometrically by determination of A260/A280 ratios (Synergy HT Microplate Reader, BioTek). Amplicon-based metagenomics was performed using the NEBNext Ultra IIDNA Library Prep Kit (Cat No. E7645).

### Microbial community composition via qPCR

Specific taxa (Firmicutes and Bacteroidetes phylum, *Enterobacteriaceae* family, *Bifidobacterium* spp., *Lactobacillus* spp., *Akkermansia muciniphila* and *Faecalibacterium prausnitzii*) were analysed by qPCR using the primers and conditions described in Supplementary Tables S1 and S2. The qPCR was performed using a QuantStudio 5 Real-Time PCR system (Applied Biosystems, Foster City, CA, USA). Each sample was run in technical triplicate, and results are reported in log-based format (16S rRNA gene copies/mL).

### 16S rRNA gene amplicon sequencing

A total of 200-ng DNA was used for PCR amplification with primer sets targeting different 16S rRNA gene hypervariable regions. Microbial community composition was assessed at defined time points by next-generation 16S rRNA gene amplicon sequencing of the V3–V4 region under contract with Novogene (UK) Company Limited (Cambridge, UK). Primers targeting the V3–V4 hypervariable region of bacterial 16S rRNA genes were as follows: 341F (5′-CCTAYGGGRBGCASCAG-3′) and 806R (5′-GGACTACNNGGGTATCTAAT-3′), where F is forward and R is reverse. Each primer set was ligated with a unique barcode sequence. The PCR product was then selected for proper size and purified for library preparation. The same amount of PCR product from each sample was pooled, end polished, A-tailed and ligated with adapters. After purification, the library was analysed for size distribution, quantified using real-time PCR and sequenced on a NovaSeq 6,000 SP flowcell with PE250 platform.

### Metabolomics

All samples were maintained at −80^o^C until processed. Samples were prepared using the automated MicroLab STAR system from Hamilton Company. To remove protein, dissociate small molecules bound to protein or trapped in the precipitated protein matrix, and to recover chemically diverse metabolites, proteins were precipitated with methanol under vigorous shaking for 2 min (Glen Mills GenoGrinder 2000) followed by centrifugation. The resulting extract was divided into five fractions: two for analysis by two separate reverse phase (RP)/ultra-performance liquid chromatography (UPLC)-MS/MS methods with positive ion mode electrospray ionization (ESI), one for analysis by RP/UPLC-MS/MS with negative ion mode ESI, one for analysis by HILIC/UPLC-MS/MS with negative ion mode ESI and one sample was reserved for backup. Samples were placed briefly on a TurboVap (Zymark) to remove the organic solvent. The sample extracts were stored overnight under nitrogen before preparation for analysis. The details of the solvents and chromatography used are already described (Ford et al., 2020).

All methods utilized a Waters ACQUITY UPLC and a Thermo Scientific Q-Exactive high-resolution/accurate mass spectrometer interfaced with a heated electrospray ionization (HESI-II) source and Orbitrap mass analyser operated at 35,000 mass resolution. The sample extract was dried then reconstituted in solvents compatible to each of the four methods. Each reconstitution solvent contained a series of standards at fixed concentrations to ensure injection and chromatographic consistency. One aliquot was analysed using acidic positive ion conditions, chromatographically optimized for more hydrophilic compounds. In this method, the extract was gradient eluted from a C18 column (Waters UPLC BEH C18-2.1 × 100 mm, 1.7 μm) using water and methanol, containing 0.05 per cent perfluoropentanoic acid (PFPA) and 0.1 per cent formic acid (FA). Another aliquot was also analysed using acidic positive ion conditions; however, it was chromatographically optimized for more hydrophobic compounds. In this method, the extract was gradient eluted from the same aforementioned C18 column using methanol, acetonitrile, water, 0.05 per cent PFPA and 0.01 per cent FA and was operated at an overall higher organic content. Another aliquot was analysed using basic negative ion optimized conditions using a separate dedicated C18 column. The basic extracts were gradient eluted from the column using methanol and water, however, with 6.5m M ammonium bicarbonate at pH 8. The fourth aliquot was analysed via negative ionization following elution from a HILIC column (Waters UPLC BEH Amide 2.1 × 150 mm, 1.7 μm) using a gradient consisting of water and acetonitrile with 10-mM ammonium formate, pH 10.8. The MS analysis alternated between MS and data-dependent MSn scans using dynamic exclusion. The scan range varied slightly between methods but covered 70–1,000 m/z. Raw data files are archived and extracted as described below.

Raw data were extracted, peak-identified and QC processed using Metabolon’s hardware and software as already described (DeHaven et al., 2010). Several controls were analysed in concert with the experimental samples and were used to calculate instrument variability (5 per cent) and overall process variability (7 per cent). Peak area values allowed the determination of relative quantification among samples (Evans et al., 2009). The present dataset comprises a total of 842 biochemicals, 721 compounds of known identity (named biochemicals) and 121 compounds of unknown structural identity (unnamed biochemicals).

### Bioinformatics

Data processing was performed on the Rosalind high-performance compute cluster (a BRC/King’s College London computing facility to quickly perform large-scale calculations with a virtual machine cloud infrastructure) using four threads and a maximum RAM of 32 GB. The DADA2 algorithm (version 1.6) was first used to correct for sequencing errors and derive amplicon sequence variants (ASVs) using the R version 3.4.1. ASVs are a higher-resolution version of the operational taxonomic unit (OTU). While OTUs are clusters of sequences with 97 per cent similarity, ASVs are unique sequences (100 per cent similarity). The use of ASVs is being advocated to replace OTU in order to improve reproducibility between studies because a threshold of 97 per cent does not allow a reproducible classification of closely related bacteria (Callahan et al., 2017).

The taxonomy was assigned using the SiLVA ribosomal RNA gene database v132. The count table, the metadata, and the sequence taxonomies were ultimately combined into a single R object, which was analysed using the phyloseq package (McMurdie and Holmes, 2013). The alpha diversity, representing the diversity of the total number of species (the richness R) within the samples, was measured using the Shannon’s diversity index (Morgan and Huttenhower, 2012). Another important diversity measure is the beta diversity, representing the diversity of the total number of species between the samples (Morgan and Huttenhower, 2012). We used the Bray–Curtis dissimilarity index, which was calculated using the Vegan R package (version 2.5.2). The analysis of 16S rRNA gene composition was conducted with a linear-mixed model with Microbiome Multivariable Association with Linear Models (MaAsLin) 2.0 (package version 0.99.12; Mallick et al., 2021). The taxonomic composition was transformed using an arcsine square root transformation. The Benjamini–Hochberg method was used to control the false discovery rate of the MaAsLin analysis.

We further analysed the 16S rRNA gene sequencing dataset and the metabolome dataset with an orthogonal partial least squares discriminant analysis (OPLS-DA), which are robust methods to analyse large datasets in particular when the number of variables is larger than the number of samples. OPLS-DA can be used to distinguish the variability corresponding to the experimental perturbation from the portion of the data that are orthogonal, ie., independent from the experimental perturbation. The R package ropls version 1.20.0 was used with a non-linear iterative partial least squares algorithm (Thévenot et al., 2015). Prior to analysis, experimental variables were centred and unit-variance scaled. Since PLS-DA methods are prone to overfitting, we assessed the significance of our classification using permutation tests (permuted 1,000 times). The variables of importance (VIPs) were extracted from the models to determine as to what are the effects of the treatments.

In addition, Calypso version 8.4 (Zakrzewski et al., 2017) and MicrobiomeAnalyst (update 18 October 2021) online software (Dhariwal et al., 2017) were used to perform discriminant analysis of principal components (DAPCs), linear discriminant analysis effect size (LEfSe), heat tree (Foster et al., 2017) and a zero inflated Gaussian fit mix model from 16S rRNA gene sequence data normalized by cumulative-sum scaling (Paulson et al., 2013) and log2 transformation to account for the non-normal distribution of taxonomic counts data.

## Results

### Pre-screening experiment to determine glyphosate dosage

A first experiment was performed to test the short-term effect of six different concentrations of glyphosate (0.5, 1, 3, 10, 100 and 1,000 mg/L) on microbial activity and *Enterobacteriaceae* levels. The European Food Safety Agency (EFSA) has established an acute reference dose and acceptable daily intake (ADI) for glyphosate of 0.5 mg/kg body weight/day (EFSA, 2015). Assuming 15–20 kg of an infant of 3 years old, this would correspond to 7.5–10 mg/day, proving the doses used in this study are regulatory relevant.

Monitoring the pH during a colonic incubation provides an overall indication of the microbial fermentative activity (Supplementary Table S3). Limited effects of glyphosate were observed although glyphosate at a concentration of 1,000 mg/L lowered the acidity of the medium, which could indicate a modulatory effect on microbial activity resulting in altered metabolite (ie., lactate and acetate) production. Total gas production was also measured to reflect the rate of substrate fermentation (Supplementary Table S3). Glyphosate tended to slightly decrease the amount of gas production, except at a concentration of 1,000 mg/L for which an increased gas production was observed. SCFA production was affected by the highest concentration of glyphosate. A lower production of acetate, propionate and b-SCFAs and higher levels of butyrate were observed compared to the untreated control culture, while most other glyphosate doses did not alter SCFA levels. We also measured *Enterobacteriaceae* levels as glyphosate is known to affect this taxonomic group by inhibiting the shikimate pathway. Overall, it was observed that glyphosate did not impact *Enterobacteriaceae* abundance at any of the concentrations tested (Supplementary Table S3).

Considering an average weight of a healthy infant of 3 years old around 15 kg, 20 per cent of absorption of glyphosate at intestinal level, and assuming a steady-state concentration in the SHIME system, it was decided to select a concentration of 100 mg/L (14-mg glyphosate added to the system in each feeding cycle) for the long-term study, which would correspond to 1.2 mg/kg of body weight of glyphosate. This dose is in the range of the U.S. ADI (1.75 mg/kg body weight/day).

### Microbial community activity during the long-term infant M-SHIME experiment

During the control period, SCFA levels were very stable within (on average 93.2 per cent similar between consecutive time points in control period), and reproducible between both of the M-SHIME units (on average 91.7 per cent similar), indicating stability and reproducibility of the experimental setup. This high stability guarantees that any effects observed during the treatment truly result from the administered test products, while the high reproducibility between each of the units allows direct comparison between the products on virtually identical microbial communities. No statistically significant differences were detected between the two different control weeks when microbial community activity was evaluated (Figure 1).

**Figure 2:**
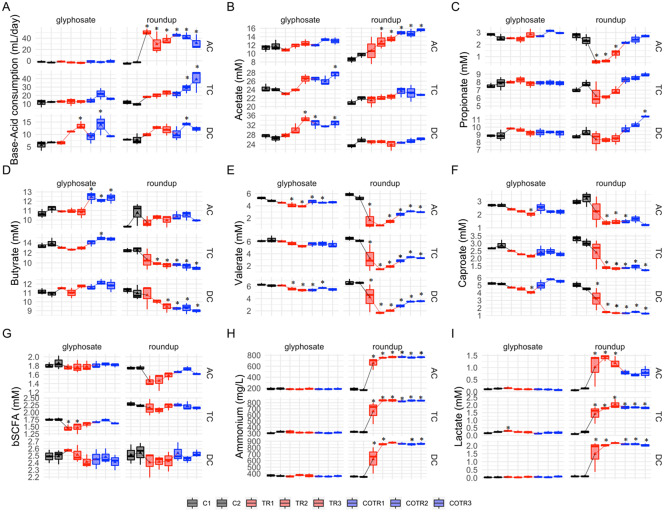
Analysis of microbial community activity after exposure to glyphosate or Roundup followed by co-exposure to the MegaSporeBiotic spore-based probiotic formulation in the microbiota derived from a healthy 3-year-old child. Average weekly base-acid consumption, SCFA levels (acetic acid, propionic acid, butyric acid, valeric and caproic acid), lactate, ammonium and b-SCFA in the culture chambers simulating the ascending (AC), transverse (TC) and descending (DC) colon during the control (black), treatment (red) and co-treatment (blue) weeks. (* Indicates statistically significant differences relative to C1 after Tukey’s honestly significant difference post hoc test with *p* < 0.05; *n* = 3 per week.)

In contrast, large product-dependent differences were observed (Figure 2). The Roundup formulation always resulted in more alterations than glyphosate alone. While base consumption remained relatively unaffected in the ascending and transverse colon reactors upon glyphosate treatment (Figure 2A), base consumption was significantly enhanced by 55.0, 6.2 and 2.0 mL/day in the ascending, transverse and descending colon, respectively, upon addition of Roundup.

Overall, glyphosate had limited effects on SCFA levels (Figure 2B–F) with acetate, propionate, butyrate, caproate and valerate levels remaining unaffected in the transverse colon, and only a slight increase in the amount of valerate (−1.09 and −0.74 mM for C2 vs. TR3 in TC and DC, respectively) and caproate (−0.71 and −1.16 mM for C2 vs. TR3 in TC and DC, respectively) was observed in the transverse and descendant colon areas. However, during the co-treatment period with MegaSporeBiotic prebiotic, an increase in the butyrate of 1.63, 1.0 and −0.1 mM (from TR3 to COTR1) was observed in the ascending, transverse and descending colon, respectively (Figure 2D).

Differences in SCFA levels were larger after exposure to Roundup (Figure 2B–F). Roundup increased acetate production by 4.9 mM in the ascending colon, whereas acetate levels remained unaffected in the transverse and descendant colon during the treatment period. The SCFAs most affected by Roundup were propionate, valerate and caproate, which had their levels reduced in the ascending and transverse colon, with a similar trend being observed in the descending colon. For instance, Roundup caused a decrease of propionate levels by 1.8 mM, valerate levels by 3.9 mM and caproate levels by 1.75 mM in the ascending colon from the end of the control period (C2) to the end of the treatment period (TR3). Interestingly, addition of MegaSporeBiotic resulted in recovery of propionate levels in all colonic regions during the Roundup treatment. In the distal colon areas (TC and DC), propionate levels even increased above levels observed during the control period (Figure 2C).

The human intestine harbours both lactate-producing and lactate-utilizing bacteria. Lactate produced by bacteria decreases the pH of the intestinal environment. Overall, treatment with glyphosate did not affect lactate levels in all colonic regions, except for a slight initial increase in the ascending and transverse colon reactors (Figure 2I). Roundup on the other hand significantly increased lactate levels in all colonic areas. The lactate levels were increased by 1 mM in the ascending colon, 1.8 mM in the transverse colon and 2 mM in the descending colon (comparison of C2 to TR3). Roundup but not glyphosate significantly increased ammonium levels in all colon regions (Figure 2H). Ammonium levels were increased by 598 mg/L in the ascending colon, 518 mg/L in the transverse colon and 526 mg/L in the descending colon (comparison of C2 to TR3).Overall, addition of MegaSporeBiotic did not affect ammonium levels during the glyphosate and Roundup treatment.

### Global metabolomics

We next explored the changes in gut microbial activity in greater detail using a global metabolomics approach. A total of 842 biochemicals (721 compounds of known identity and 121 compounds of unknown structural identity) were detected. Concentrations of SCFA, which were measured quantitatively and detected in the global untargeted metabolomics, correlated well for caproate (*R* ^2^ = 0.96; *p*-value < 2e-16), butyrate (*R* ^2^ = 0.82; *p*-value = 8.0e-13) and valerate (*R* ^2^ = 0.93; *p*-value < 2.2e-16), and confirmed the quality of our data (Supplementary Figure S1).

**Figure 3:**
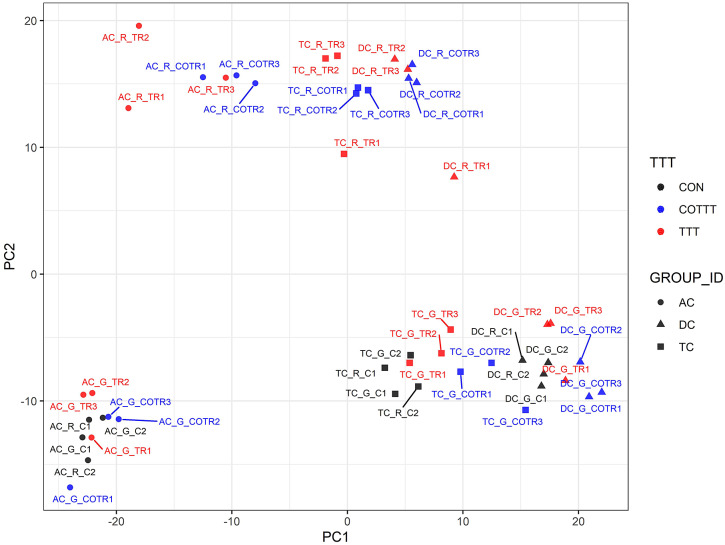
Principal component (PC) analysis to understand source of variation in the global metabolome profiles indicates that Roundup causes large-scale disturbances in microbial activity. The metabolome changes are visualized by plotting each sample in a space defined by the two principal components of a PC analysis. The name of each sample is defined by capital letters providing indication on the metadata including colon section [ascending (AC), transverse (TC) and descending (DC)], herbicide treatment [glyphosate (G) or Roundup (R)] and treatment time/time condition [control (C1, C2; black), treatment (TR1, TR2, TR3; red) and co-treatment (COTR1, COTR2, COTR3; blue)]. The first (PC1) and second (PC2) components of the PCA explained 31 and 20.5 per cent of the total variance.

We used a multivariate strategy to understand the effects of glyphosate and Roundup in the different SHIME compartments. The metabolome changes were first visualized by plotting each sample in a space defined by the two principal components of a principal component analysis (PCA; Figure 3). The first component separated the group of samples by colon regions. The second component separated the samples exposed to the Roundup formulation from the other samples, whereas samples exposed to glyphosate did not separate from the control group. In order to understand which metabolites were driving these differences stemming from the treatment with Roundup, we built an OPLS-DA model on the basis of the PCA results (Supplementary Figure S2). The OPLS-DA model separating the samples exposed to Roundup from the rest of the samples, appropriately classified all samples (R2X = 0.314, R2Y = 0.991 and Q2 = 0.93). The small difference between R2Y and Q2 (<0.2) and the Q2 value greater than 50 per cent revealed an excellent predictive capability. A 1,000-time permutation test further validated the OPLS-DA model as the empirical *p*-values for R2Y (*p* = 0.001), and Q2 (*p* = 0.001) indicated that the observed effects of Roundup are not part of the distribution formed by permuted data. OPLS-DA models built by discriminating the samples according to their exposure to glyphosate, or to MegaSporeBiotic, were not found to be of sufficient quality to allow reliable conclusions and were thus not explored in greater detail.

**Figure 4:**
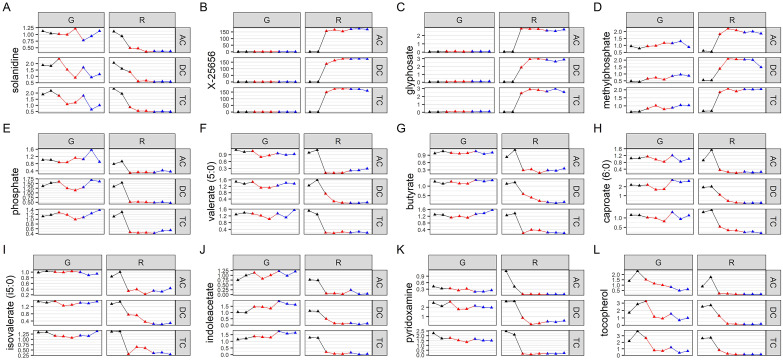
Variation in abundance of metabolites mostly affected by Roundup exposure. Normalised abundance levels are presented for key metabolites in glyphosate (G) and Roundup (R) SHIME in the ascending (AC), transverse (TC) and descending colon (DC) during the control (black), treatment (red) and co-treatment (blue) weeks, among 125 metabolites discriminating the Roundup exposed samples in an OPLS-DA model (Supplementary Figure S8).

We then evaluated which metabolites were affected by Roundup, and also performed a metabolic pathway enrichment analysis. The metabolites which are the best discriminators were selected using VIP scores. A selection of metabolites, which were found to be relevant for glyphosate toxicology, is presented in Figure 4A–L, whereas plots for the 125 metabolites with a VIP score of >1.5 are available as Supplementary Figure S10. Although glyphosate levels were unsurprisingly found to be the most variable (Figure 4C), the levels of this compound were affected only by the Roundup treatment. This could suggest that the surfactant co-formulants present in Roundup increased glyphosate availability. The most variable metabolite increased after treatment with Roundup was found to be one of unknown structural identities (Figure 4B), which suggests that glyphosate or Roundup metabolic pathways are still not fully elucidated. Levels of SCFAs (Figure 4F–I) were decreased, confirming the results of the targeted analysis of microbial activity (Figure 1). Overall, most of the metabolites which were altered by Roundup exposure had their levels decreased, and were part of many microbial biochemical pathways, such as vitamin or hormone precursors (Figure 4J–), which suggests that this pesticide mixture exerts global inhibitory effects on microbial metabolism. Methylphosphate (Figure 4D) was among the few metabolites having its levels increased by Roundup exposure. In addition, we noticed the solanidine had its levels decreased by both Roundup and glyphosate exposure (Figure 4). Aromatic amino acids, which have their levels decreased in plants following the shikimate pathway inhibition by glyphosate, were relatively unchanged, although some trends could suggest that further studies with a higher statistical power could reveal some effects (Supplementary Figure S3). Statistical significance of pathway enrichment was tested using a two-sided hypergeometric test. This revealed that Roundup affected the pathways for long-chain polyunsaturated fatty acid (PUFA; n3 and n6) metabolism (*p* = 0.00003), SCFA metabolism (*p* = 0.02), (hypo)xanthine/inosine containing purine metabolism (*p* = 0.04) and xenobiotic chemical metabolism (*p* = 0.003). While most of the SCFA fatty acids had their levels decreased, PUFAs (n3 and n6) were among the small proportion of metabolites which had their abundance increased by Roundup exposure, including docosapentaenoate (22:5n3 and 22:5n6), docosahexaenoate (22:6n3), eicosapentaenoate (20:5n3), dihomo-linolenate (20:3n3 and 20:2n6), linoleate (18:2n6), arachidonate (20:4n6) and cis-4-decenoate (10:1n6; Supplementary Figure S10).

### Microbial community composition during the long-term M-SHIME culture

Previous studies have reported that glyphosate and its formulations exerted a wide spectrum effects on bacterial growth and affected gut microbial community composition at the phylum level, in particular for Bacteroidetes phylum and Firmicutes phylum. In order to evaluate whether we can identify which bacterial species are affected by glyphosate and Roundup, we sequenced amplicons of the V3–V4 regions from the 16S rRNA genes.

In the present study, qPCR was also used to monitor the abundance of taxa with known health roles. This analysis was done both in the luminal and the mucosal gut microbiota populations. The results from the qPCR experiments for the Bacteroidetes phylum and Firmicutes phyla (Supplementary Figure S4), as well as for *Akkermansia muciniphila*, *Faecalibacterium prausnitzii, Bifidobacterium spp. and Lactobacillus spp.* (Supplementary Figure S4), presented a substantial degree of technical variation, which limited the conclusiveness of this compositional analysis. This is reflected by the variations exhibited during the control period, with large changes in bacterial abundance observed between C1 and C2. In addition, poor correlations between the results of the qPCR and the 16S RNA gene analysis for major taxa such as Firmicutes and Bacteroidetes can be observed (Supplementary Figure S5). Thus, our analysis of bacterial composition only provided preliminary evidence, which would need to be confirmed in other studies. It is, however, noteworthy that there is a trend towards an increase in *Lactobacillus spp.* levels caused by the exposure to glyphosate and Roundup in both the luminal and mucosal compartments (Supplementary Figure S6A). Lactobacilli are regarded as beneficial saccharolytic bacteria that are capable of producing high concentrations of lactate. The increase in lactobacilli could thus explain the increase in lactate levels in all colon compartments upon Roundup and glyphosate treatments (Figures 3 and 4).

The Firmicutes-to-Bacteroidetes (F/B) ratio has been proposed as a potential biomarker of gut dysbiosis associated with specific diseased conditions (Magne et al., 2020). Glyphosate significantly reduced F/B ratio in the ascending and distal colon in the luminal compartment (*p* < 0.05), a trend also observed for the mucus environment in all the compartments. Contrastingly, Roundup treatment increased F/B ratio in the luminal (DC) and mucosal (AC and TC) compartments (*p* < 0.05) (Supplementary Table S4). Co-treatment with the spore-based probiotic MegaSporeBiotic did not rescue F/B ratios.

**Figure 5:**
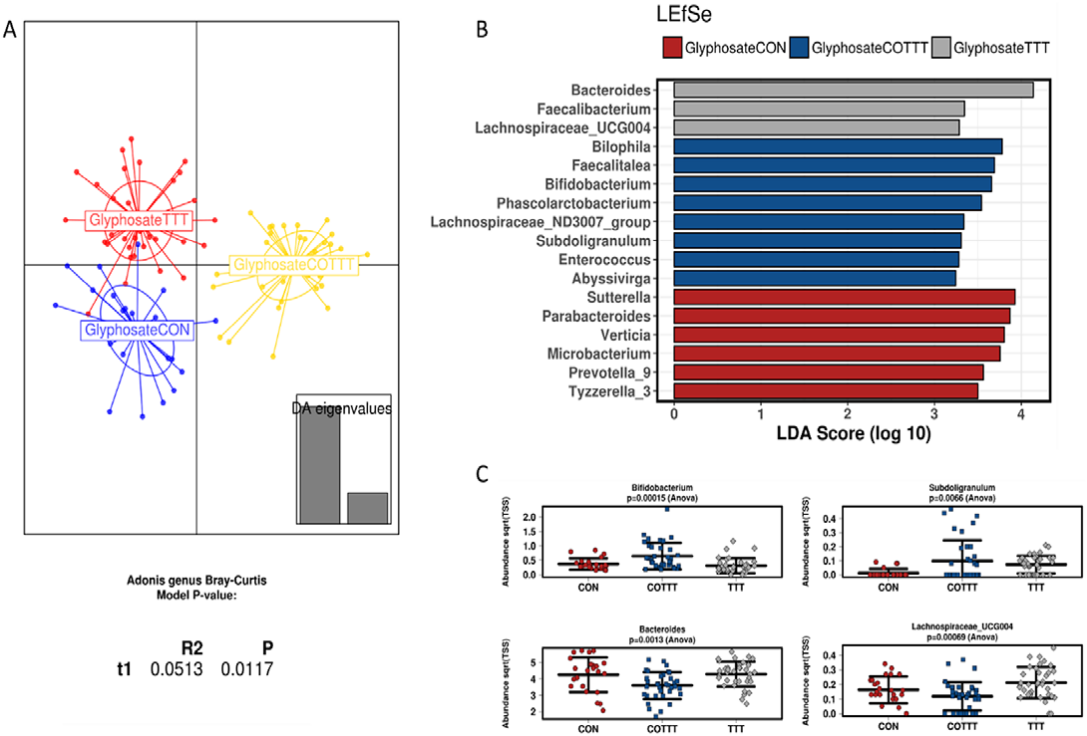
Effect of glyphosate on infant gut microbial structure. (A) Discriminant analysis of principal components and Adonis test based on the Bray–Curtis distance at genus level. (B) Linear discriminant analysis effect size for the glyphosate arm during the control (CON), treatment (TTT) and co-treatment (COTT) conditions. (C) Strip char plots of selected features significantly different (ANOVA; *p* < 0.05) between conditions.

Microbiota DNA sequencing provided an average of 112,230 reads for each sample (min 57,313–max 139,760). A summary of the composition for the different compartments during the control period indicated that the gut microbiome was dominated by Firmicutes and Bacteroidetes (Supplementary Figure S7). The phylum Verrucomicrobia accounting for approximately 5 per cent of the assigned abundance was mainly represented by *Akkermansia muciniphila.* This taxonomic profile is typical of people from Western societies. At lower taxonomic levels, we observed that a total of three genera accounted for approximately 70 per cent of the assigned abundance (Figure 5A). This included the *Megasphaera* spp., which are generally not among the most abundant species in the general population from Western societies, but which is abundant in the gut microbiome from individuals whose diet is rich in dairy products, which can be the case for young children (Dhakan et al., 2019).

Analysing glyphosate and Roundup treatments together, the DAPC plot showed a different clustering based on control, treatment or co-treatment (*p* < 0.05; Adonis test at genus level; Supplementary Figure S8A). The only family affected by treatments was *Lactobacillaceae,* due to an enrichment in glyphosate or Roundup treated reactors (LDA > 3), whereas co-treatment enriched *Synergistaceae, Desulfovibrionaceae, Acidaminococcaceae* and *Bifidobacteriaceae* (LDA > 3) among others (Supplementary Figure S8B). To define the effect of specific treatments on infant gut microbiota, samples from glyphosate or Roundup conditions were independently analysed. Samples from glyphosate treatment and further co-treatment with MegaSporeBiotic probiotic clustered separately from the control (*p* < 0.05; Adonis at genus level; Figure 5A). Samples from the control condition were enriched in *Sutterella, Parabacteroides, Verticia, Microbacterium, Prevotella* and *Tyzerella* genera (LDA > 3). During the glyphosate treatment, an enrichment in *Bacteroides, Faecalibacterium* and *Lachnospiraceae_*UCG004 was observed. Finally, probiotic treatment enriched *Bilophila, Faecalitalea, Bifidobacterium, Subdoligranulum* and *Phascolarctobacterium*, among other genera (Figure 5B,C).

**Figure 6:**
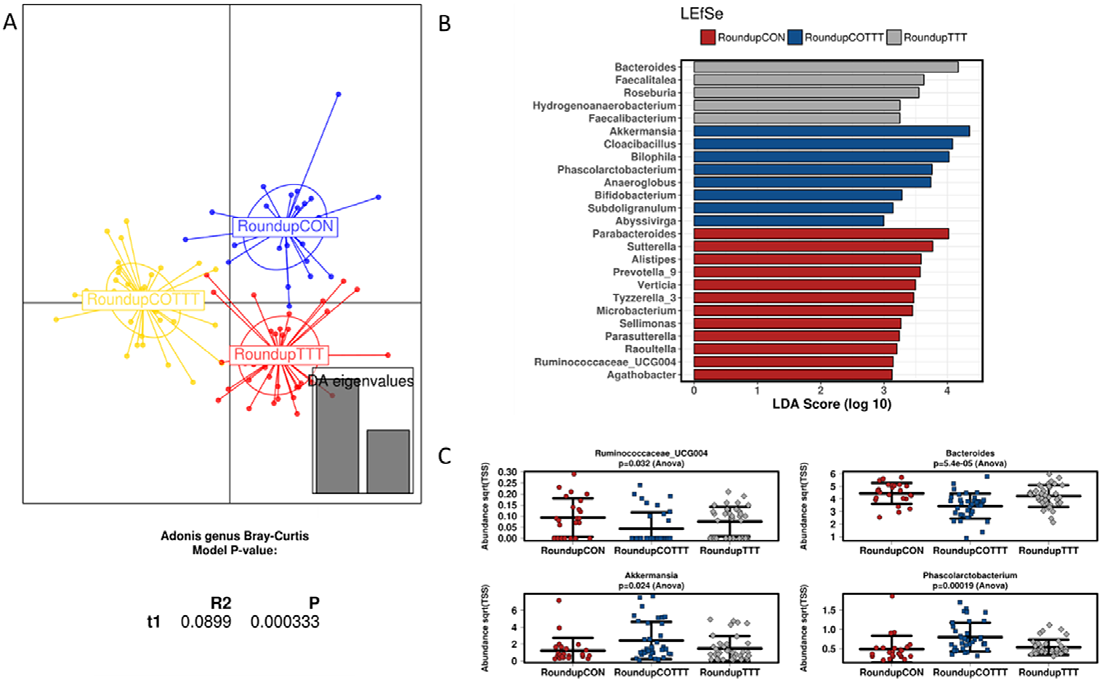
Effect of Roundup on infant gut microbial structure. (A) Discriminant analysis of principal components and Adonis test based on the Bray–Curtis distance at genus level. (B) Linear discriminant analysis effect size for the Roundup arm during the control (CON), treatment (TTT) and co-treatment (COTT) conditions. (C) Strip char plots of selected features significantly different (ANOVA; *p* < 0.05) between conditions.

Roundup treatment also induced a different clustering from control and co-treatment (*p* < 0.0001; Adonis at genus level; Figure 6A), with enrichment in *Bacteroides*, *Faecalitalea, Roseburia, Hydrogenoanaerobacterium* and *Faecalibacterium.* MegaSporeBiotic probiotic co-treatment enriched, in addition to some genera described for the glyphosate condition, *Akkermansia* and *Cloacibacillus.* When analysing the data using a non-metric multidimensional scaling of Bray–Curtis distances (Supplementary Figure S9), no clear clustering based on treatment was observed, suggesting a potential effect of time on composition profiles.

**Figure 7:**
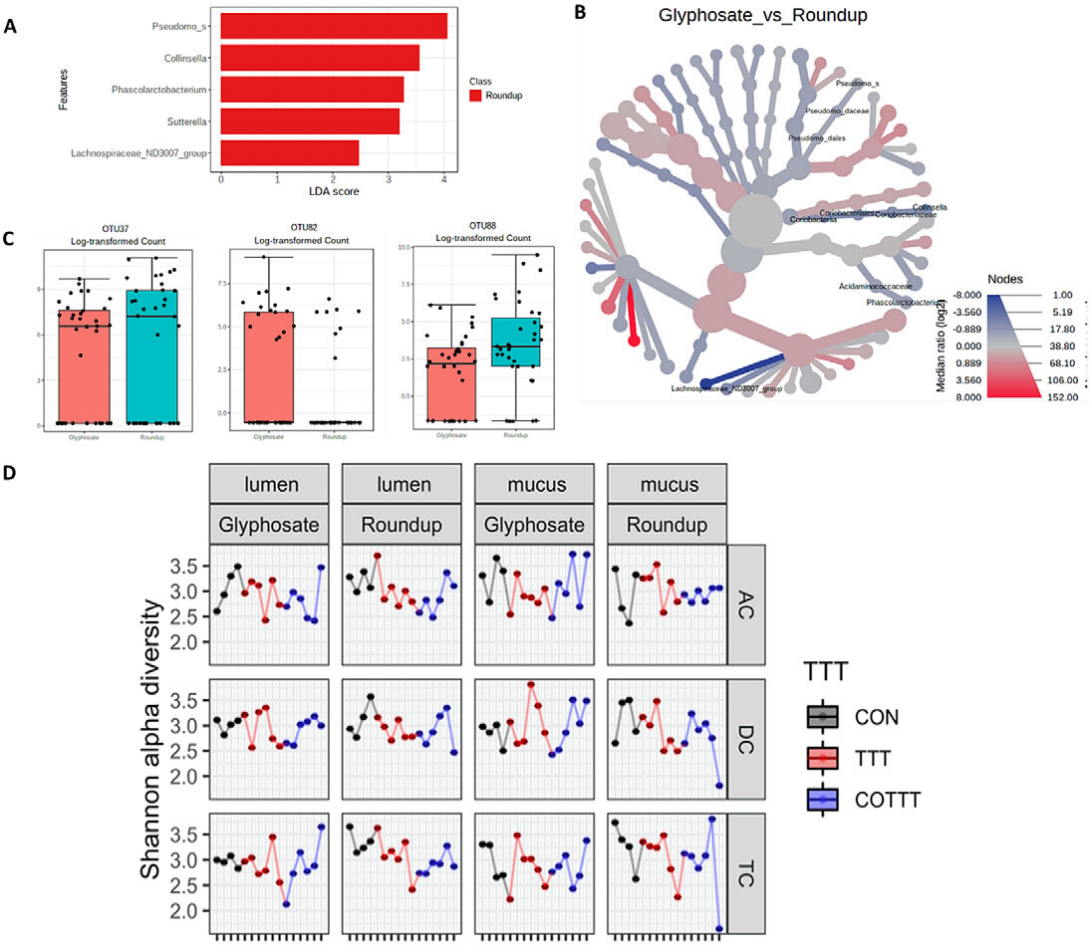
A 16S rRNA gene amplicon sequencing approach to assess alterations in gut microbiome composition. (A) Linear discriminant analysis effect size including treatment samples from glyphosate and Roundup treatment arms of the study. (B) Heat tree analysis of differences between glyphosate and Roundup treatments at genus level. Significantly different taxa between groups are written in the figure.

Glyphosate and Roundup treatments were further compared, showing increased levels of *Pseudomonadaceae, Collinsella*, *Phascolarctobacterium, Sutterella* and *Lachnospiraceae*_ND3007_group in the presence of Roundup (LEfSe, LDA > 2, Figure 7A; heat tree based on non-parametric Wilcoxon Rank Sum test, Figure 7B). At a lower phylogenetic level, OTU37 (*Bacteroides* spp.), OTU82 (*Klebsiella* spp.) and OTU88 (*Collinsella* spp.) were significantly different between glyphosate and Roundup treated cultures (Figure 7C; zero-inflated Gaussian fit mixed model MicrobiomeAnalyst software).

We found a statistically significant difference in average bacterial Shannon alpha diversity (Figure 7C) by the treatments (*p* = 0.02), but it was not different between glyphosate and Roundup (*p* = 0.42), between the mucus and the lumen (*p* = 0.87) or between the different SHIME compartments (*p* = 0.64). Tukey multiple comparisons of means further showed that alpha diversity decreased with the pesticide treatment (*p* = 0.03), and later recovered after exposure to MegaSporeBiotic (Figure 7C).

As for the analysis employing qPCR, the changes in bacterial composition derived from 16S rRNA gene sequencing presented a substantial degree of technical variation reflected by the variations exhibited during the control period as illustrated for the most frequently found genera *Megasphaera*, *Bacteroides*, *Lachnoclostridium* and *Akkermansia* (Supplementary Figure S7).

Altogether, our study of the changes in gut microbial activity and composition after a long-term exposure to glyphosate and Roundup reveals profound changes in the fermentation activity and metabolite profiles, while changes in composition profiles were less pronounced with the current experimental design.

## Discussion

We describe for the first time the effects of glyphosate and a formulated glyphosate Roundup herbicide on human gut microbiota. Using an *in vitro* technology mimicking the entire gastrointestinal tract revealed that glyphosate caused large-scale disturbances in the activity of gut microbiota obtained from a healthy infant.

Both glyphosate and Roundup caused a change in the fermentation activity of the infant gut microbiota as reflected by the increase in base consumption. This is because optimal environmental conditions are maintained in the SHIME by base addition in order to maintain the pH, which can change as a result of bacterial fermentation. This acidification of the gut microbial ecosystem by glyphosate can be linked to an increase in the production of acetate and lactate, as bacterial production of these compounds tends to acidify the medium. This in turn favours the growth of bacteria, which can tolerate a lower pH such as lactobacilli which had their growth increased during the treatment period in this study. Despite the fact that specific *Lactobacillus* and *Bifidobacterium* strains are currently accepted as probiotics (Hill et al., 2014), composition of mucosal-associated microbes in inflammatory bowel patients has shown increased proportions of Bifidobacteria and lactobacilli, against a decrease in butyrate-producing bacteria (Wang et al., 2014). This study suggests that imbalance between gut commensals might influence host inflammatory responses. This also correlates well with findings of our recent laboratory animal study showing that rats exposed prenatally until adulthood to glyphosate presented increased levels of lactobacilli (Mesnage et al., 2021a).

Glyphosate exposure levels have been reported in a small number of studies, with variable levels found in occupationally, para-occupationally or environmentally individuals, with ranges from 0.16 to 73.5 μg/L in urine (Gillezeau et al., 2019, 2020). We applied a dose of 100 mg/L, which would correspond to 1.1 mg/kg of oral exposure, taking into account infant weight, repeated dose regimen, colonic volumes and glyphosate absorption in the intestinal tract. Fecal levels of glyphosate are not usually reported in human biomonitoring studies, and data are derived from animal. The no-observed-adverse-effect level (NOAEL) and ADI of glyphosate are currently set at 50 and 0.5 mg/Kg bw, respectively. Glyphosate intake in these levels would derive in fecal contents around 35–40 mg/Kg bw/day or 0.35–0.4 mg/Kg bw/day, if considering 20–30 per cent of oral bioavailability. This would represent 525–600 mg (NOAEL) or 5.3–6 mg (ADI) of glyphosate in feces for an infant (15 kg). Considering colonic volume of healthy infants around 0.2 L (Sharif et al., 2021), NOAEL levels of glyphosate could represent 2,625–3,000 mg/L in feces, and ADI levels of glyphosate 100-time lower values. We used 100 mg/L, a dose in between these two reference values.

The pH of the gut environment is key in maintaining ecosystem homeostasis, and it has been described as the strongest driver for microbial community structure *in vitro* (Ilhan Zehra et al., 2017). When the pH of the gut microbiome environment decreases, it impairs the growth of pH sensitive bacteria such as *Bacteroides* spp (Walker et al., 2005) and inhibits lactate-consuming species, leading to lactate accumulation (Ilhan Zehra et al., 2017). Lactate accumulation has been linked to butyrate and propionate reduction, due to major shifts in microbiota composition, with Bacteroidetes and anaerobic Firmicutes being substituted by Actinobacteria, lactobacilli and Proteobacteria (Ilhan Zehra et al., 2017). Since some members of the Bacteroides family are known propionate or butyrate producers (Walker et al., 2005), the acidification of the gut microbial environment by glyphosate is also likely to explain the decrease in specific bacterial metabolites. This is also visible in our recent animal study in which Bacteroidota abundance was decreased by glyphosate exposure (Mesnage et al., 2021a). In addition, reductions in butyrate and propionate induced by pesticides can be potentially caused by alterations in enzymatic paths involved in cross-feeding interactions, eg., involving co-A transferase (Louis and Flint, 2017) or the acrylate pathway.

Microbial SCFAs are essential for maintaining gut homeostasis through different mechanisms involving control of gut barrier integrity, regulating the luminal pH, mucus production, providing energy for epithelial cells and modulating mucosal immune function (Blaak et al., 2020). Lactate accumulation has been described in colitis patients (Vernia et al., 1988), and it can also be used as a growth substrate by sulphate-reducing gut bacteria (Marquet et al., 2009), promoting the formation of toxic concentrations of hydrogen sulphide. Reduction of butyrate and propionate production and lactate accumulation induced by a GBH indicates functional dysbiosis in our *in vitro* SHIME system. However, potential consequences on the host still require further investigation.

Both the production of ammonium and b-SCFA result from protein degradation and reflect proteolytic activity of the gut microbiota. Ammonium production was substantially increased by Roundup exposure but not by glyphosate. This could be associated with the increased abundance of lactobacilli as the higher metabolic activity of this class of bacteria can enhance deamination processes, which is the main pathway of amino acid fermentation in humans. It is worth noting that an increased production of ammonium is detrimental because it can activate cell proliferation mechanisms in colonocytes and subsequently promote colon carcinogenesis (Visek, 1978).

Global metabolomics revealed disturbances in a large number of metabolic pathways. However, the relevance to health of most of these changes are still elusive. This is the case for solanidine levels, which were decreased by both Roundup and glyphosate exposure. This is particularly interesting because this has already been described in our study involving subchronic exposure to another Roundup formulation in the rat gut microbiome (Mesnage et al., 2021b), and in potato plants grown in glyphosate-treated soil (Rainio et al., 2020). Methylphosphate was among the few metabolites, which had its abundance stimulated by Roundup. Since glyphosate is an organophosphate compound, this observation may originate from previously unknown biotransformation of glyphosate. In another bioreactor study investigating the porcine gut microbiota, it was found that AMPA was produced by the bacterial communities from the degradation of glyphosate (Fritz-Wallace et al., 2020). A more recent study of the metabolism of glyphosate *in vitro* by the use of human fecal suspension samples found that biotransformation of glyphosate seems to be negligible in human gut microbiota from 15 healthy adult humans (Huch et al., 2021)

Perhaps the most surprising metabolome changes we observed were the increase in levels of n3 and n6 long-chain PUFAs. Some limited groups of bacteria can synthetize distinct unsaturated fatty acids such as arachidonic acid (20:4 n-6), eicosapentaenoic acid (20:5 n-3) and docosahexaenoic acid (22:6 n-3) (Okuyama et al., 2007; Yoshida et al., 2016). Anaerobic *de novo* synthesis of PUFA is mediated by the PUFA synthase complex, but only described in environmental microorganisms up to now (Metz et al., 2001; Okuyama et al., 2007; Yoshida et al., 2016). On the other hand, human gut microbiota modulates PUFA metabolism, involving multiple microbial species (Ewaschuk et al., 2006; McIntosh et al., 2009). Recently, docosahexaenoic acid and arachidonic acid were positively correlated with *Bacteroides* in patients with chronic spontaneous urticaria (Wang et al., 2020). It is also possible that PUFAs are formed from the metabolism of Roundup surfactants, which are in general synthetized from vegetable oils or animal fat (Mesnage et al., 2019). However, surfactants employed in formulating Roundup herbicides are generally considered as confidential business information by the manufacturer and thus not generally disclosed (Mesnage et al., 2019). Therefore, it is not fully clear if the blend of surfactant entering in the composition of Roundup PROMAX used in this investigation could contain surfactants synthetized from PUFA-containing oils.

The increase of long-chain fatty acids has been positively correlated with an elevated abundance of *Lactobacillus* in a rodent model of neonatal maternal separation characterized by accelerated colonic motility and gut dysbiosis, suggesting a relevant role of these molecules on host-microbiota interplay and potentially affecting host metabolism (Zhao et al., 2018). Fatty acid profiles in aquatic ecosystems have been proposed as biomarkers of pesticide exposure (Gonçalves et al., 2021). Short-term exposure to glyphosate or Roundup in the sea cucumber *Holothuria forskali* perturbed fatty acid composition, including some essential fatty acids (Telahigue et al., 2021). Changes in composition of PUFAs, ie., alteration in the n3/n6 ratio, have been associated with increased incidence and prevalence of being overweight and obesity, linked to inflammatory responses (Jovanovic et al., 2021). Altogether, we propose pesticide disruption of fatty acid human gut metabolome as a potential biomarker of exposure, and also a mechanism of metabolic disturbance potentially influencing the host. Given the large number of changes detected in pathways for long-chain PUFAs, future studies could benefit from the use of more targeted and quantitative metabolome profiling methods such as lipidomics.

Whereas structural changes based on 16S rRNA gene sequencing did not indicate dramatic shifts on microbial composition, alpha diversity was reduced by exposure to both glyphosate and Roundup. Lower alpha diversity indices during infancy have been correlated with lower cognitive performance (Carlson et al., 2018), and negative health outcomes, including type 1 diabetes (Kostic et al., 2015) and asthma (Abrahamsson et al., 2014). The dynamic alterations in functional capacity of the simulated infant gut ecosystem suggest an impact of glyphosate or Roundup exposure on host function. A study in rats assessing prenatal exposure to glyphosate or two Roundup formulations showed the gut microbiome of F1 offspring was affected by both treatments, with a reduction in Bacteroidota abundance, concomitant to increased levels of Firmicutes and Actinobacteria (Mesnage et al., 2021a).

Within the temporal framework used in this study, we observed a time-course modulation of the microbial metabolic landscape suggesting structural resilience of the infant gut ecosystem to glyphosate or Roundup exposure, but a functional dysbiosis affecting key microbial metabolites for microbiota-host cross-talk. For example, carboxyethyl-gamma aminobutyric acid (GABA) was significantly reduced in the ascending colon after the Roundup treatment. GABA is linked to glutamate metabolism and the gut–brain axis and different microbial taxa regulate the GABAergic system in the human gut, including *Bifidobacterium* spp. (Duranti et al., 2020) and *Bacteroides* spp (Otaru et al., 2021).

Our results suggest that the addition of MegaSporeBiotic spore-based probiotic affected the gut microbiota and mitigated glyphosate-induced changes. Some Bacillus species are adapted to survive in the intestine (Cartman et al., 2008; Tam et al., 2006). In such cases, spores ingested with food are able to survive transit through the stomach after which they germinate and proliferate, a phenomenon that has been proven using *in vivo* studies (Cartman et al., 2008; Tam et al., 2006). Following growth and proliferation, they re-sporulate as the bacteria pass through the intestine and are shed into the environment. Such germination and re-sporulation would be a necessity for certain health benefits, as was shown for the stimulation of the development of the gut-associated lymphoid tissue in rabbits by *B. subtilis* (Rhee et al., 2004). However, our study does not distinguish as to whether the Bacillus spores mitigated the effects of glyphosate by directly counteracting its effects, or if they add effects which are independent of glyphosate presence. Caution is also needed when extrapolating these findings to real-world glyphosate exposure. Our results and previous studies in animal model systems suggest structural and metabolic shifts in the gut environment due to exposure to pesticide residues (Tsiaoussis et al., 2019). The effect of these changes on human health remains to be determined. In addition, the conclusiveness of this study is very limited by its experimental design since it is very difficult to generalise conclusions from a study performed with one just biological sample. In addition, it is likely that the statistical power did not allow the detection of effects for taxa present a low abundance (less than 5 per cent) in the gut microbiome samples. Further experiments will be needed to validate these first observations.

## Data Availability

Data are available upon reasonable request. Please direct enquiries to the corresponding author.

## Supplementary material

For supplementary material accompanying this paper visit https://doi.org/10.1017/gmb.2022.5. click here to view supplementary material
