# Supplementary material for: Alterations in infant gut microbiome composition and metabolism after exposure to glyphosate and Roundup and/or a spore-based formulation using the SHIME technology
Source: Gut Microbiome (Camb). 2022 Jul 26;3:e6. doi: 10.1017/gmb.2022.5 (PMC11406414; doi:10.1017/gmb.2022.5)
Supplement: Supplementary file 1 [file gmbsup.zip › S2632289722000056sup003.pdf]

SCFA

Base-Acid

```
## [1] "For glyphosate in AC"
```

```
##             Df Sum Sq Mean Sq F value Pr(>F)
## Week         7  25.29   3.613   0.998  0.468
## Residuals   16  57.94   3.621
```

```
##   Tukey multiple comparisons of means
##     95% family-wise confidence level
##
## Fit: aov(formula = Value ~ Week, data = BaseAcid_G_AC)
##
## $Week
##                    diff       lwr      upr     p adj
## C2-C1       -1.11659333 -6.495883 4.262697 0.9950413
## COTR1-C1     0.21853833 -5.160752 5.597828 0.9999999
## COTR2-C1    -0.88965833 -6.268948 4.489632 0.9987866
## COTR3-C1     1.31999667 -4.059293 6.699287 0.9867968
## TR1-C1       0.25366222 -5.125628 5.632952 0.9999997
## TR2-C1      -1.20975667 -6.589047 4.169533 0.9920176
## TR3-C1      -2.16439333 -7.543683 3.214897 0.8478094
## COTR1-C2     1.33513167 -4.044158 6.714422 0.9859168
## COTR2-C2     0.22693500 -5.152355 5.606225 0.9999999
## COTR3-C2     2.43659000 -2.942700 7.815880 0.7613947
## TR1-C2       1.37025556 -4.009035 6.749546 0.9837060
## TR2-C2      -0.09316333 -5.472453 5.286127 1.0000000
## TR3-C2      -1.04780000 -6.427090 4.331490 0.9966294
## COTR2-COTR1 -1.10819667 -6.487487 4.271093 0.9952617
## COTR3-COTR1  1.10145833 -4.277832 6.480748 0.9954329
## TR1-COTR1    0.03512389 -5.344166 5.414414 1.0000000
## TR2-COTR1   -1.42829500 -6.807585 3.950995 0.9795032
## TR3-COTR1   -2.38293167 -7.762222 2.996358 0.7796848
## COTR3-COTR2  2.20965500 -3.169635 7.588945 0.8346387
## TR1-COTR2    1.14332056 -4.235970 6.522611 0.9942859
## TR2-COTR2   -0.32009833 -5.699388 5.059192 0.9999987
## TR3-COTR2   -1.27473500 -6.654025 4.104555 0.9891829
## TR1-COTR3   -1.06633444 -6.445625 4.312956 0.9962483
## TR2-COTR3   -2.52975333 -7.909043 2.849537 0.7284832
## TR3-COTR3   -3.48439000 -8.863680 1.894900 0.3784428
## TR2-TR1     -1.46341889 -6.842709 3.915871 0.9766035
## TR3-TR1     -2.41805556 -7.797346 2.961235 0.7677722
## TR3-TR2     -0.95463667 -6.333927 4.424653 0.9981069
```

```
## [1] "For glyphosate in DC"
```

```
##             Df Sum Sq Mean Sq F value   Pr(>F)
## Week         7 202.40  28.914   7.268 0.000674 ***
## Residuals   15  59.68   3.979
```

```
## ---
## Signif. codes:  0 '***' 0.001 '**' 0.01 '*' 0.05 '.' 0.1 ' ' 1
## 1 observation deleted due to missingness
```

```
##   Tukey multiple comparisons of means
##     95% family-wise confidence level
##
## Fit: aov(formula = Value ~ Week, data = BaseAcid_G_DC)
##
## $Week
##                   diff         lwr        upr     p adj
## C2-C1        1.0506611  -4.6381086  6.7394308 0.9973796
## COTR1-C1     3.1992767  -2.4894931  8.8880464 0.5328193
## COTR2-C1     8.2318600   2.5430903 13.9206297 0.0027201
## COTR3-C1     3.4750333  -2.2137364  9.1638031 0.4378463
## TR1-C1       0.8992622  -4.7895075  6.5880319 0.9990148
## TR2-C1       5.4127350  -0.9475029 11.7729729 0.1252443
## TR3-C1       7.7154017   2.0266319 13.4041714 0.0049035
## COTR1-C2     2.1486156  -3.5401542  7.8373853 0.8779255
## COTR2-C2     7.1811989   1.4924292 12.8699686 0.0090666
## COTR3-C2     2.4243722  -3.2643975  8.1131419 0.8021118
## TR1-C2      -0.1513989  -5.8401686  5.5373708 1.0000000
## TR2-C2       4.3620739  -1.9981640 10.7223118 0.3094495
## TR3-C2       6.6647406   0.9759708 12.3535103 0.0164492
## COTR2-COTR1  5.0325833  -0.6561864 10.7213531 0.1024349
## COTR3-COTR1  0.2757567  -5.4130131  5.9645264 0.9999997
## TR1-COTR1   -2.3000144  -7.9887842  3.3887553 0.8384749
## TR2-COTR1    2.2134583  -4.1467796  8.5736962 0.9149330
## TR3-COTR1    4.5161250  -1.1726447 10.2048947 0.1742946
## COTR3-COTR2 -4.7568267 -10.4455964  0.9319431 0.1366706
## TR1-COTR2   -7.3325978 -13.0213675 -1.6438281 0.0076145
## TR2-COTR2   -2.8191250  -9.1793629  3.5411129 0.7715789
## TR3-COTR2   -0.5164583  -6.2052281  5.1723114 0.9999750
## TR1-COTR3   -2.5757711  -8.2645408  3.1129986 0.7538220
## TR2-COTR3    1.9377017  -4.4225362  8.2979396 0.9551214
## TR3-COTR3    4.2403683  -1.4484014  9.9291381 0.2276000
## TR2-TR1      4.5134728  -1.8467651 10.8737107 0.2745181
## TR3-TR1      6.8161394   1.1273697 12.5049092 0.0138151
## TR3-TR2      2.3026667  -4.0575712  8.6629046 0.8983979
```

```
## [1] "For glyphosate in TC"
```

```
##             Df Sum Sq Mean Sq F value Pr(>F)
## Week         7  180.0   25.72   1.729  0.177
## Residuals   15  223.2   14.88
## 1 observation deleted due to missingness
```

```
##   Tukey multiple comparisons of means
##     95% family-wise confidence level
##
## Fit: aov(formula = Value ~ Week, data = BaseAcid_G_TC)
##
## $Week
##                   diff        lwr       upr     p adj
## C2-C1        0.7918133 -10.208756 11.792383 0.9999949
## COTR1-C1     2.5355189  -8.465050 13.536088 0.9901249
```

```
## COTR2-C1    10.5784967  -1.720514 22.877507 0.1186768
## COTR3-C1     4.3069467  -6.693623 15.307516 0.8581333
## TR1-C1       1.7449244  -9.255645 12.745494 0.9989929
## TR2-C1       1.2936300  -9.706939 12.294199 0.9998573
## TR3-C1       1.0646239  -9.935945 12.065193 0.9999614
## COTR1-C2     1.7437056  -9.256864 12.744275 0.9989974
## COTR2-C2     9.7866833  -2.512327 22.085694 0.1724712
## COTR3-C2     3.5151333  -7.485436 14.515703 0.9431634
## TR1-C2       0.9531111 -10.047458 11.953680 0.9999818
## TR2-C2       0.5018167 -10.498753 11.502386 0.9999998
## TR3-C2       0.2728106 -10.727759 11.273380 1.0000000
## COTR2-COTR1  8.0429778  -4.256033 20.341988 0.3607907
## COTR3-COTR1  1.7714278  -9.229142 12.771997 0.9988914
## TR1-COTR1   -0.7905944 -11.791164 10.209975 0.9999949
## TR2-COTR1   -1.2418889 -12.242458  9.758680 0.9998913
## TR3-COTR1   -1.4708950 -12.471464  9.529674 0.9996667
## COTR3-COTR2 -6.2715500 -18.570560  6.027460 0.6403668
## TR1-COTR2   -8.8335722 -21.132583  3.465438 0.2626319
## TR2-COTR2   -9.2848667 -21.583877  3.014144 0.2161983
## TR3-COTR2   -9.5138727 -21.812883  2.785138 0.1952441
## TR1-COTR3   -2.5620222 -13.562592  8.438547 0.9895150
## TR2-COTR3   -3.0133167 -14.013886  7.987253 0.9741657
## TR3-COTR3   -3.2423227 -14.242892  7.758247 0.9620696
## TR2-TR1     -0.4512944 -11.451864 10.549275 0.9999999
## TR3-TR1     -0.6803005 -11.680870 10.320269 0.9999982
## TR3-TR2     -0.2290061 -11.229575 10.771563 1.0000000

## [1] "For Roundup in AC"

##             Df Sum Sq Mean Sq F value  Pr(>F)
## Week         7   9423  1346.1   14.75 6.8e-06 ***
## Residuals   16   1461    91.3
## ---
## Signif. codes:  0 '***' 0.001 '**' 0.01 '*' 0.05 '.' 0.1 ' ' 1

##   Tukey multiple comparisons of means
##     95% family-wise confidence level
##
## Fit: aov(formula = Value ~ Week, data = BaseAcid_R_AC)
##
## $Week
##                  diff        lwr       upr     p adj
## C2-C1        1.798054 -25.210788 28.806897 0.9999973
## COTR1-C1     50.541469  23.532627 77.550311 0.0001623
## COTR2-C1     47.282641  20.273799 74.291483 0.0003457
## COTR3-C1     35.935497   8.926654 62.944339 0.0054874
## TR1-C1       55.033430  28.024588 82.042272 0.0000593
## TR2-C1       34.025136   7.016293 61.033978 0.0088467
## TR3-C1       41.491047  14.482204 68.499889 0.0013872
## COTR1-C2     48.743414  21.734572 75.752257 0.0002457
## COTR2-C2     45.484587  18.475744 72.493429 0.0005289
## COTR3-C2     34.137442   7.128600 61.146284 0.0086017
## TR1-C2       53.235376  26.226533 80.244218 0.0000883
## TR2-C2       32.227081   5.218239 59.235923 0.0138642
## TR3-C2       39.692992  12.684150 66.701834 0.0021574
```

```
## COTR2-COTR1  -3.258828 -30.267670 23.750014 0.9998444
## COTR3-COTR1 -14.605972 -41.614814 12.402870 0.5858006
## TR1-COTR1     4.491961 -22.516881 31.500803 0.9987426
## TR2-COTR1   -16.516333 -43.525176 10.492509 0.4445658
## TR3-COTR1    -9.050422 -36.059264 17.958420 0.9322089
## COTR3-COTR2 -11.347144 -38.355987 15.661698 0.8193867
## TR1-COTR2     7.750789 -19.258053 34.759631 0.9688815
## TR2-COTR2   -13.257506 -40.266348 13.751337 0.6878577
## TR3-COTR2    -5.791594 -32.800437 21.217248 0.9939757
## TR1-COTR3    19.097933  -7.910909 46.106776 0.2834651
## TR2-COTR3    -1.910361 -28.919203 25.098481 0.9999958
## TR3-COTR3     5.555550 -21.453292 32.564392 0.9953057
## TR2-TR1     -21.008294 -48.017137  6.000548 0.1940473
## TR3-TR1     -13.542383 -40.551226 13.466459 0.6665725
## TR3-TR2       7.465911 -19.542931 34.474753 0.9745002
```

```
## [1] "For Roundup in DC"
```

```
##             Df Sum Sq Mean Sq F value  Pr(>F)
## Week         7 101.28  14.468   4.916 0.00469 **
## Residuals   15  44.14   2.943
## ---
## Signif. codes:  0 '***' 0.001 '**' 0.01 '*' 0.05 '.' 0.1 ' ' 1
## 1 observation deleted due to missingness
```

```
##   Tukey multiple comparisons of means
##     95% family-wise confidence level
##
## Fit: aov(formula = Value ~ Week, data = BaseAcid_R_DC)
##
## $Week
##                    diff         lwr        upr     p adj
## C2-C1       -0.03285611 -5.50301275  5.4373005 1.0000000
## COTR1-C1     2.28491167 -3.18524498  7.7550683 0.8165791
## COTR2-C1     6.30395833  0.83380169 11.7741150 0.0186451
## COTR3-C1     4.29980833 -1.17034831  9.7699650 0.1821241
## TR1-C1       2.00726167 -3.46289498  7.4774183 0.8922069
## TR2-C1       4.88230278 -0.58785387 10.3524594 0.0976594
## TR3-C1       3.99314750 -1.47700914  9.4633041 0.2470025
## COTR1-C2     2.31776778 -2.57488906  7.2104246 0.7134020
## COTR2-C2     6.33681444  1.44415760 11.2294713 0.0073110
## COTR3-C2     4.33266444 -0.55999240  9.2253213 0.1018841
## TR1-C2       2.04011778 -2.85253906  6.9327746 0.8178038
## TR2-C2       4.91515889  0.02250205  9.8078157 0.0485573
## TR3-C2       4.02600361 -0.86665323  8.9186605 0.1476743
## COTR2-COTR1  4.01904667 -0.87361017  8.9117035 0.1488936
## COTR3-COTR1  2.01489667 -2.87776017  6.9075535 0.8263535
## TR1-COTR1   -0.27765000 -5.17030684  4.6150068 0.9999990
## TR2-COTR1    2.59739111 -2.29526573  7.4900480 0.5972726
## TR3-COTR1    1.70823583 -3.18442101  6.6008927 0.9136676
## COTR3-COTR2 -2.00415000 -6.89680684  2.8885068 0.8299397
## TR1-COTR2   -4.29669667 -9.18935351  0.5959602 0.1065041
## TR2-COTR2   -1.42165556 -6.31431240  3.4710013 0.9647577
## TR3-COTR2   -2.31081083 -7.20346767  2.5818460 0.7162003
## TR1-COTR3   -2.29254667 -7.18520351  2.6001102 0.7235128
```

```
## TR2-COTR3    0.58249444 -4.31016240  5.4751513 0.9998451
## TR3-COTR3   -0.30666083 -5.19931767  4.5859960 0.9999980
## TR2-TR1      2.87504111 -2.01761573  7.7676980 0.4824886
## TR3-TR1      1.98588583 -2.90677101  6.8785427 0.8359547
## TR3-TR2     -0.88915528 -5.78181212  4.0035016 0.9976288
```

```
## [1] "For Roundup in TC"
```

```
##             Df Sum Sq Mean Sq F value   Pr(>F)    
## Week         7 1836.7  262.38   7.495 0.000442 ***
## Residuals   16  560.1   35.01                     
## ---
## Signif. codes:  0 '***' 0.001 '**' 0.01 '*' 0.05 '.' 0.1 ' ' 1
```

```
##   Tukey multiple comparisons of means
##     95% family-wise confidence level
## 
## Fit: aov(formula = Value ~ Week, data = BaseAcid_R_TC)
## 
## $Week
##                   diff         lwr        upr     p adj
## C2-C1        -2.548371 -19.2737738 14.1770316 0.9992821
## COTR1-C1      9.573584  -7.1518183 26.2989871 0.5213052
## COTR2-C1     16.479118  -0.2462849 33.2045205 0.0549931
## COTR3-C1     27.164923  10.4395206 43.8903260 0.0007793
## TR1-C1        6.170407 -10.5549960 22.8958094 0.8945852
## TR2-C1        7.950668  -8.7747349 24.6760705 0.7185734
## TR3-C1       11.100190  -5.6252127 27.8255927 0.3512237
## COTR1-C2     12.121956  -4.6034471 28.8473583 0.2587061
## COTR2-C2     19.027489   2.3020862 35.7528916 0.0201401
## COTR3-C2     29.713294  12.9878917 46.4386971 0.0002934
## TR1-C2        8.718778  -8.0066249 25.4441805 0.6259214
## TR2-C2       10.499039  -6.2263638 27.2244416 0.4142883
## TR3-C2       13.648561  -3.0768416 30.3739638 0.1560753
## COTR2-COTR1   6.905533  -9.8198694 23.6309360 0.8312598
## COTR3-COTR1  17.591339   0.8659362 34.3167416 0.0356445
## TR1-COTR1    -3.403178 -20.1285805 13.3222249 0.9956036
## TR2-COTR1    -1.622917 -18.3483194 15.1024860 0.9999640
## TR3-COTR1     1.526606 -15.1987971 18.2520083 0.9999762
## COTR3-COTR2  10.685806  -6.0395971 27.4112083 0.3940673
## TR1-COTR2   -10.308711 -27.0341138  6.4166916 0.4354300
## TR2-COTR2    -8.528450 -25.2538527  8.1969527 0.6492502
## TR3-COTR2    -5.378928 -22.1043305 11.3464749 0.9444268
## TR1-COTR3   -20.994517 -37.7199194 -4.2691140 0.0091216
## TR2-COTR3   -19.214256 -35.9396583 -2.4888529 0.0186875
## TR3-COTR3   -16.064733 -32.7901360  0.6606694 0.0644638
## TR2-TR1       1.780261 -14.9451416 18.5056638 0.9999327
## TR3-TR1       4.929783 -11.7956194 21.6551860 0.9642162
## TR3-TR2       3.149522 -13.5758805 19.8749249 0.9972623
```

Acetate

```
## [1] "For glyphosate in AC"
```

```
##             Df Sum Sq Mean Sq F value Pr(>F)  
## Week         7 14.483  2.0690   3.547  0.017 *
```

```
## Residuals   16  9.333  0.5833
## ---
## Signif. codes:  0 '***' 0.001 '**' 0.01 '*' 0.05 '.' 0.1 ' ' 1
##   Tukey multiple comparisons of means
##     95% family-wise confidence level
##
## Fit: aov(formula = Value ~ Week, data = Acetate_G_AC)
##
## $Week
##                    diff        lwr         upr     p adj
## C2-C1        0.45320644 -1.7057902  2.61220313 0.9946956
## COTR1-C1     0.61341610 -1.5455806  2.77241279 0.9704793
## COTR2-C1     2.04390991 -0.1150868  4.20290660 0.0703789
## COTR3-C1     1.64101377 -0.5179829  3.80001045 0.2140773
## TR1-C1      -0.48134804 -2.6403447  1.67764864 0.9924138
## TR2-C1       0.49732118 -1.6616755  2.65631786 0.9908183
## TR3-C1       1.08591363 -1.0730831  3.24491032 0.6633924
## COTR1-C2     0.16020966 -1.9987870  2.31920635 0.9999942
## COTR2-C2     1.59070347 -0.5682932  3.74970016 0.2427977
## COTR3-C2     1.18780733 -0.9711894  3.34680401 0.5665324
## TR1-C2      -0.93455448 -3.0935512  1.22444221 0.7976627
## TR2-C2       0.04411474 -2.1148820  2.20311143 1.0000000
## TR3-C2       0.63270719 -1.5262895  2.79170388 0.9652704
## COTR2-COTR1  1.43049381 -0.7285029  3.58949050 0.3530623
## COTR3-COTR1  1.02759767 -1.1313990  3.18659436 0.7174065
## TR1-COTR1   -1.09476414 -3.2537608  1.06423255 0.6550464
## TR2-COTR1   -0.11609492 -2.2750916  2.04290177 0.9999994
## TR3-COTR1    0.47249753 -1.6864992  2.63149422 0.9932005
## COTR3-COTR2 -0.40289614 -2.5618928  1.75610055 0.9974112
## TR1-COTR2   -2.52525795 -4.6842546 -0.36626126 0.0162468
## TR2-COTR2   -1.54658873 -3.7055854  0.61240796 0.2703000
## TR3-COTR2   -0.95799628 -3.1169930  1.20100041 0.7783459
## TR1-COTR3   -2.12236181 -4.2813585  0.03663488 0.0557937
## TR2-COTR3   -1.14369259 -3.3026893  1.01530410 0.6085494
## TR3-COTR3   -0.55510014 -2.7140968  1.60389655 0.9828386
## TR2-TR1      0.97866922 -1.1803275  3.13766591 0.7607610
## TR3-TR1      1.56726167 -0.5917350  3.72625836 0.2571399
## TR3-TR2      0.58859245 -1.5704042  2.74758914 0.9763341
## [1] "For glyphosate in DC"
##             Df Sum Sq Mean Sq F value   Pr(>F)
## Week         7 179.05   25.58   12.01 2.59e-05 ***
## Residuals   16  34.09    2.13
## ---
## Signif. codes:  0 '***' 0.001 '**' 0.01 '*' 0.05 '.' 0.1 ' ' 1
##   Tukey multiple comparisons of means
##     95% family-wise confidence level
##
## Fit: aov(formula = Value ~ Week, data = Acetate_G_DC)
##
## $Week
##                     diff        lwr        upr     p adj
## C2-C1       -0.945133944 -5.0710675  3.1807996 0.9911105
```

```
## COTR1-C1     5.232089981  1.1061564  9.3580235 0.0083632
## COTR2-C1     3.974056231 -0.1518773  8.0999898 0.0633661
## COTR3-C1     5.228393505  1.1024599  9.3543271 0.0084139
## TR1-C1       0.312385790 -3.8135478  4.4383193 0.9999934
## TR2-C1       2.654316014 -1.4716175  6.7802496 0.3862004
## TR3-C1       7.111406772  2.9854732 11.2373403 0.0004106
## COTR1-C2     6.177223925  2.0512904 10.3031575 0.0017963
## COTR2-C2     4.919190175  0.7932566  9.0451237 0.0139527
## COTR3-C2     6.173527449  2.0475939 10.2994610 0.0018070
## TR1-C2       1.257519734 -2.8684138  5.3834533 0.9575049
## TR2-C2       3.599449958 -0.5264836  7.7253835 0.1116566
## TR3-C2       8.056540716  3.9306072 12.1824743 0.0000987
## COTR2-COTR1 -1.258033750 -5.3839673  2.8678998 0.9574166
## COTR3-COTR1 -0.003696475 -4.1296300  4.1222371 1.0000000
## TR1-COTR1   -4.919704191 -9.0456377 -0.7937706 0.0139410
## TR2-COTR1   -2.577773966 -6.7037075  1.5481596 0.4197309
## TR3-COTR1    1.879316792 -2.2466168  6.0052504 0.7566711
## COTR3-COTR2  1.254337274 -2.8715963  5.3802708 0.9580491
## TR1-COTR2   -3.661670441 -7.7876040  0.4642631 0.1018409
## TR2-COTR2   -1.319740216 -5.4456738  2.8061933 0.9458875
## TR3-COTR2    3.137350541 -0.9885830  7.2632841 0.2137068
## TR1-COTR3   -4.916007716 -9.0419413 -0.7900742 0.0140254
## TR2-COTR3   -2.574077491 -6.7000110  1.5518561 0.4213872
## TR3-COTR3    1.883013267 -2.2429203  6.0089468 0.7549917
## TR2-TR1      2.341930225 -1.7840033  6.4678638 0.5309702
## TR3-TR1      6.799020983  2.6730874 10.9249545 0.0006681
## TR3-TR2      4.457090758  0.3311572  8.5830243 0.0295266

## [1] "For glyphosate in TC"

##             Df Sum Sq Mean Sq F value  Pr(>F)
## Week         7  54.59   7.799    11.8 2.9e-05 ***
## Residuals   16  10.58   0.661
## ---
## Signif. codes:  0 '***' 0.001 '**' 0.01 '*' 0.05 '.' 0.1 ' ' 1

##   Tukey multiple comparisons of means
##     95% family-wise confidence level
##
## Fit: aov(formula = Value ~ Week, data = Acetate_G_TC)
##
## $Week
##                    diff         lwr       upr     p adj
## C2-C1       -0.27554724 -2.57414551 2.0230510 0.9998510
## COTR1-C1     2.21584613 -0.08275214 4.5144444 0.0630394
## COTR2-C1     1.67025451 -0.62834376 3.9688528 0.2561709
## COTR3-C1     3.23686017  0.93826190 5.5354584 0.0032535
## TR1-C1      -1.29551674 -3.59411501 1.0030815 0.5390834
## TR2-C1      -0.37864052 -2.67723879 1.9199578 0.9988170
## TR3-C1       2.23055071 -0.06804756 4.5291490 0.0605112
## COTR1-C2     2.49139337  0.19279509 4.7899916 0.0288273
## COTR2-C2     1.94580175 -0.35279652 4.2444000 0.1304609
## COTR3-C2     3.51240741  1.21380914 5.8110057 0.0014638
## TR1-C2      -1.01996950 -3.31856777 1.2786288 0.7783232
## TR2-C2      -0.10309328 -2.40169156 2.1955050 0.9999998
```

```
## TR3-C2       2.50609795  0.20749967  4.8046962 0.0276282
## COTR2-COTR1 -0.54559162 -2.84418989  1.7530067 0.9890808
## COTR3-COTR1  1.02101404 -1.27758423  3.3196123 0.7774998
## TR1-COTR1   -3.51136286 -5.80996114 -1.2127646 0.0014682
## TR2-COTR1   -2.59448665 -4.89308492 -0.2958884 0.0213786
## TR3-COTR1    0.01470458 -2.28389369  2.3133029 1.0000000
## COTR3-COTR2  1.56660566 -0.73199261  3.8652039 0.3221637
## TR1-COTR2   -2.96577125 -5.26436952 -0.6671730 0.0072008
## TR2-COTR2   -2.04889503 -4.34749330  0.2497032 0.0994372
## TR3-COTR2    0.56029620 -1.73830207  2.8588945 0.9872872
## TR1-COTR3   -4.53237691 -6.83097518 -2.2337786 0.0000879
## TR2-COTR3   -3.61550069 -5.91409897 -1.3169024 0.0010894
## TR3-COTR3   -1.00630946 -3.30490774  1.2922888 0.7889828
## TR2-TR1      0.91687621 -1.38172206  3.2154745 0.8530871
## TR3-TR1      3.52606744  1.22746917  5.8246657 0.0014074
## TR3-TR2      2.60919123  0.31059296  4.9077895 0.0204825

## [1] "For Roundup in AC"

##             Df Sum Sq Mean Sq F value   Pr(>F)
## Week         7 143.40  20.486   9.278 0.000128 ***
## Residuals   16  35.33   2.208
## ---
## Signif. codes:  0 '***' 0.001 '**' 0.01 '*' 0.05 '.' 0.1 ' ' 1

##   Tukey multiple comparisons of means
##     95% family-wise confidence level
##
## Fit: aov(formula = Value ~ Week, data = Acetate_R_AC)
##
## $Week
##                   diff         lwr         upr     p adj
## C2-C1        1.1052301 -3.09518336  5.30564353 0.9804972
## COTR1-C1     6.3343613  2.13394787 10.53477475 0.0016714
## COTR2-C1     6.1680870  1.96767354 10.36850043 0.0021745
## COTR3-C1     6.9640885  2.76367509 11.16450198 0.0006260
## TR1-C1       2.0994886 -2.10092479  6.29990209 0.6697695
## TR2-C1       4.2818524  0.08143896  8.48226584 0.0440767
## TR3-C1       4.8928144  0.69240093  9.09322782 0.0167800
## COTR1-C2     5.2291312  1.02871778  9.42954467 0.0097813
## COTR2-C2     5.0628569  0.86244345  9.26327034 0.0127768
## COTR3-C2     5.8588585  1.65844501 10.05927189 0.0035588
## TR1-C2       0.9942586 -3.20615488  5.19467200 0.9892526
## TR2-C2       3.1766223 -1.02379113  7.37703576 0.2185918
## TR3-C2       3.7875843 -0.41282915  7.98799773 0.0932883
## COTR2-COTR1 -0.1662743 -4.36668777  4.03413912 0.9999999
## COTR3-COTR1  0.6297272 -3.57068622  4.83014067 0.9993533
## TR1-COTR1   -4.2348727 -8.43528610 -0.03445922 0.0474071
## TR2-COTR1   -2.0525089 -6.25292235  2.14790453 0.6922901
## TR3-COTR1   -1.4415469 -5.64196038  2.75886651 0.9241128
## COTR3-COTR2  0.7960016 -3.40441189  4.99641500 0.9971531
## TR1-COTR2   -4.0685983 -8.26901178  0.13181511 0.0612040
## TR2-COTR2   -1.8862346 -6.08664802  2.31417886 0.7686057
## TR3-COTR2   -1.2752726 -5.47568605  2.92514084 0.9583339
## TR1-COTR3   -4.8645999 -9.06501333 -0.66418645 0.0175546
```

```
## TR2-COTR3   -2.6822361 -6.88264958  1.51817731 0.3946606
## TR3-COTR3   -2.0712742 -6.27168760  2.12913928 0.6833310
## TR2-TR1      2.1823638 -2.01804969  6.38277719 0.6294765
## TR3-TR1      2.7933257 -1.40708771  6.99373917 0.3489907
## TR3-TR2      0.6109620 -3.58945147  4.81137542 0.9994684
```

```
## [1] "For Roundup in DC"
```

```
##             Df Sum Sq Mean Sq F value Pr(>F)
## Week         7  19.08   2.726   2.187 0.0923 .
## Residuals   16  19.94   1.246
## ---
## Signif. codes:  0 '***' 0.001 '**' 0.01 '*' 0.05 '.' 0.1 ' ' 1
```

```
##   Tukey multiple comparisons of means
##     95% family-wise confidence level
##
## Fit: aov(formula = Value ~ Week, data = Acetate_R_DC)
##
## $Week
##                   diff        lwr       upr     p adj
## C2-C1        2.4526584 -0.7028517 5.6081685 0.1946643
## COTR1-C1     1.3253575 -1.8301526 4.4808676 0.8195751
## COTR2-C1     2.0703514 -1.0851587 5.2258615 0.3639990
## COTR3-C1     2.8120191 -0.3434910 5.9675292 0.0995727
## TR1-C1       1.5974345 -1.5580756 4.7529446 0.6567471
## TR2-C1       1.7664308 -1.3890793 4.9219409 0.5468544
## TR3-C1       0.4634794 -2.6920307 3.6189895 0.9994336
## COTR1-C2    -1.1273009 -4.2828110 2.0282092 0.9086998
## COTR2-C2    -0.3823070 -3.5378171 2.7732031 0.9998401
## COTR3-C2     0.3593607 -2.7961494 3.5148708 0.9998941
## TR1-C2      -0.8552239 -4.0107340 2.3002862 0.9770755
## TR2-C2      -0.6862276 -3.8417377 2.4692825 0.9934507
## TR3-C2      -1.9891790 -5.1446891 1.1663311 0.4094364
## COTR2-COTR1  0.7449939 -2.4105162 3.9005040 0.9894115
## COTR3-COTR1  1.4866616 -1.6688485 4.6421717 0.7268240
## TR1-COTR1    0.2720770 -2.8834331 3.4275871 0.9999838
## TR2-COTR1    0.4410733 -2.7144368 3.5965834 0.9995897
## TR3-COTR1   -0.8618780 -4.0173881 2.2936321 0.9760932
## COTR3-COTR2  0.7416677 -2.4138424 3.8971778 0.9896812
## TR1-COTR2   -0.4729169 -3.6284270 2.6825932 0.9993547
## TR2-COTR2   -0.3039206 -3.4594307 2.8515895 0.9999657
## TR3-COTR2   -1.6068720 -4.7623821 1.5486381 0.6506449
## TR1-COTR3   -1.2145846 -4.3700947 1.9409255 0.8733672
## TR2-COTR3   -1.0455883 -4.2010984 2.1099218 0.9357519
## TR3-COTR3   -2.3485397 -5.5040498 0.8069704 0.2332651
## TR2-TR1      0.1689963 -2.9865138 3.3245064 0.9999994
## TR3-TR1     -1.1339551 -4.2894652 2.0215550 0.9062383
## TR3-TR2     -1.3029514 -4.4584615 1.8525587 0.8312016
```

```
## [1] "For Roundup in TC"
```

```
##             Df Sum Sq Mean Sq F value Pr(>F)
## Week         7  16.51   2.359   2.012  0.117
## Residuals   16  18.76   1.172
```

```
##   Tukey multiple comparisons of means
```

```
##     95% family-wise confidence level
##
## Fit: aov(formula = Value ~ Week, data = Acetate_R_TC)
##
## $Week
##                   diff        lwr       upr     p adj
## C2-C1        1.0446356 -2.0162376 4.1055088 0.9260682
## COTR1-C1     2.6876871 -0.3731861 5.7485603 0.1078636
## COTR2-C1     2.2897830 -0.7710902 5.3506562 0.2285189
## COTR3-C1     1.8398272 -1.2210460 4.9007004 0.4645764
## TR1-C1       0.5425620 -2.5183112 3.6034352 0.9981208
## TR2-C1       1.1967317 -1.8641415 4.2576049 0.8647549
## TR3-C1       1.4294206 -1.6314525 4.4902938 0.7348150
## COTR1-C2     1.6430515 -1.4178217 4.7039246 0.5940174
## COTR2-C2     1.2451474 -1.8157258 4.3060206 0.8409284
## COTR3-C2     0.7951916 -2.2656816 3.8560647 0.9818215
## TR1-C2      -0.5020736 -3.5629468 2.5587996 0.9988485
## TR2-C2       0.1520961 -2.9087771 3.2129693 0.9999996
## TR3-C2       0.3847850 -2.6760882 3.4456582 0.9997958
## COTR2-COTR1 -0.3979041 -3.4587773 2.6629691 0.9997451
## COTR3-COTR1 -0.8478599 -3.9087331 2.2130133 0.9742144
## TR1-COTR1   -2.1451251 -5.2059982 0.9157481 0.2926589
## TR2-COTR1   -1.4909554 -4.5518285 1.5699178 0.6953723
## TR3-COTR1   -1.2582664 -4.3191396 1.8026067 0.8341432
## COTR3-COTR2 -0.4499558 -3.5108290 2.6109174 0.9994306
## TR1-COTR2   -1.7472210 -4.8080942 1.3136522 0.5244779
## TR2-COTR2   -1.0930513 -4.1539245 1.9678219 0.9088665
## TR3-COTR2   -0.8603624 -3.9212355 2.2005108 0.9721162
## TR1-COTR3   -1.2972652 -4.3581383 1.7636080 0.8131956
## TR2-COTR3   -0.6430955 -3.7039687 2.4177777 0.9946672
## TR3-COTR3   -0.4104066 -3.4712797 2.6504666 0.9996876
## TR2-TR1      0.6541697 -2.4067035 3.7150429 0.9940942
## TR3-TR1      0.8868586 -2.1740146 3.9477318 0.9672729
## TR3-TR2      0.2326889 -2.8281843 3.2935621 0.9999932
```

Propionate

```r
### Propionate ####
Propionate <- microbial_activity[microbial_activity$Compound == 'Propionate',]

C <- ggplot(data = Propionate, aes(x = Time, y = Value, colour = Time, fill = Time)) +
  geom_boxplot(alpha = 0.5, outlier.color = 'white') +
  stat_summary(aes(group = 1), geom = "point", fun = mean, shape = 17, size = 1) +
  stat_summary(aes(group = 1), geom = "line",  size = 0.2, fun = mean) +
  scale_x_discrete(breaks = c(1,2,3,4,5,6,7,8), labels = c("C", "C", "TR", "TR", "TR", "COTR", "COTR",
  scale_fill_manual(values = c("black", "black", "red", "red", "red", "blue", "blue", "blue")) +
  scale_colour_manual(values = c("black", "black", "red", "red", "red", "blue", "blue", "blue")) +
  ylab("Propionate (mM)") +
  facet_grid(Compartment ~ Arm, scales = 'free') + theme_bw() +
  theme(axis.text.x = element_text(angle = 90, vjust = 0.5, hjust=1), text = element_text(size = 12)) +
  theme(legend.position = "none", axis.title.x=element_blank(), axis.text.x =element_blank()) + labs(ta

print("For glyphosate in AC")
```

```
## [1] "For glyphosate in AC"
```

```r
Propionate_G_AC <- Propionate[Propionate$Arm == 'glyphosate' & Propionate$Compartment == 'AC',]
res.aov <- aov(Value ~ Week, data = Propionate_G_AC)
summary(res.aov)
```

```
##             Df Sum Sq Mean Sq F value Pr(>F)
## Week         7  1.181  0.1687   3.708 0.0142 *
## Residuals   16  0.728  0.0455
## ---
## Signif. codes:  0 '***' 0.001 '**' 0.01 '*' 0.05 '.' 0.1 ' ' 1
```

```r
TukeyHSD(res.aov, which = "Week")
```

```
##   Tukey multiple comparisons of means
##     95% family-wise confidence level
##
## Fit: aov(formula = Value ~ Week, data = Propionate_G_AC)
##
## $Week
##                    diff         lwr         upr     p adj
## C2-C1       -0.27811006 -0.88109858  0.32487846 0.7458632
## COTR1-C1    -0.12772939 -0.73071791  0.47525913 0.9943997
## COTR2-C1     0.30935669 -0.29363184  0.91234521 0.6428493
## COTR3-C1     0.11887244 -0.48411609  0.72186096 0.9963724
## TR1-C1      -0.30662783 -0.90961636  0.29636069 0.6521025
## TR2-C1      -0.38208923 -0.98507775  0.22089929 0.4034846
## TR3-C1       0.02968498 -0.57330354  0.63267350 0.9999997
## COTR1-C2     0.15038067 -0.45260785  0.75336919 0.9855304
## COTR2-C2     0.58746675 -0.01552177  1.19045527 0.0590309
## COTR3-C2     0.39698250 -0.20600603  0.99997102 0.3601682
## TR1-C2      -0.02851777 -0.63150630  0.57447075 0.9999997
## TR2-C2      -0.10397917 -0.70696769  0.49900936 0.9984195
## TR3-C2       0.30779504 -0.29519348  0.91078356 0.6481480
## COTR2-COTR1  0.43708608 -0.16590244  1.04007460 0.2585663
## COTR3-COTR1  0.24660183 -0.35638670  0.84959035 0.8375082
## TR1-COTR1   -0.17889844 -0.78188697  0.42409008 0.9629818
## TR2-COTR1   -0.25435984 -0.85734836  0.34862869 0.8165414
## TR3-COTR1    0.15741437 -0.44557415  0.76040289 0.9813269
## COTR3-COTR2 -0.19048425 -0.79347277  0.41250427 0.9491244
## TR1-COTR2   -0.61598452 -1.21897304 -0.01299600 0.0434584
## TR2-COTR2   -0.69144591 -1.29443444 -0.08845739 0.0189536
## TR3-COTR2   -0.27967171 -0.88266023  0.32331682 0.7409292
## TR1-COTR3   -0.42550027 -1.02848879  0.17748825 0.2855658
## TR2-COTR3   -0.50096166 -1.10395019  0.10202686 0.1431796
## TR3-COTR3   -0.08918746 -0.69217598  0.51380107 0.9994074
## TR2-TR1     -0.07546139 -0.67844992  0.52752713 0.9998018
## TR3-TR1      0.33631281 -0.26667571  0.93930134 0.5510331
## TR3-TR2      0.41177421 -0.19121431  1.01476273 0.3200493
```

```r
print("For glyphosate in DC")
```

```
## [1] "For glyphosate in DC"
```

```r
Propionate_G_DC <- Propionate[Propionate$Arm == 'glyphosate' & Propionate$Compartment == 'DC',]
res.aov <- aov(Value ~ Week, data = Propionate_G_DC)
summary(res.aov)
```

```
##             Df Sum Sq Mean Sq F value Pr(>F)
## Week         7  2.413  0.3447   1.774  0.162
## Residuals   16  3.109  0.1943
```

```r
TukeyHSD(res.aov, which = "Week")
```

```
##   Tukey multiple comparisons of means
##     95% family-wise confidence level
##
## Fit: aov(formula = Value ~ Week, data = Propionate_G_DC)
##
## $Week
##                    diff        lwr       upr     p adj
## C2-C1        0.06353492 -1.1826314 1.3097013 0.9999996
## COTR1-C1     0.45510804 -0.7910583 1.7012744 0.8992078
## COTR2-C1     0.50096544 -0.7452009 1.7471318 0.8483478
## COTR3-C1     0.33947975 -0.9066866 1.5856461 0.9764296
## TR1-C1       0.97306565 -0.2731007 2.2192320 0.1907971
## TR2-C1       0.84625612 -0.3999102 2.0924225 0.3260684
## TR3-C1       0.36338193 -0.8827844 1.6095483 0.9661602
## COTR1-C2     0.39157312 -0.8545932 1.6377395 0.9504549
## COTR2-C2     0.43743053 -0.8087358 1.6835969 0.9157418
## COTR3-C2     0.27594483 -0.9702215 1.5221112 0.9927123
## TR1-C2       0.90953073 -0.3366356 2.1556971 0.2518620
## TR2-C2       0.78272120 -0.4634451 2.0288875 0.4136039
## TR3-C2       0.29984701 -0.9463193 1.5460134 0.9881933
## COTR2-COTR1  0.04585741 -1.2003089 1.2920238 1.0000000
## COTR3-COTR1 -0.11562828 -1.3617946 1.1305381 0.9999734
## TR1-COTR1    0.51795761 -0.7282087 1.7641240 0.8267773
## TR2-COTR1    0.39114808 -0.8550183 1.6373144 0.9507225
## TR3-COTR1   -0.09172611 -1.3378925 1.1544402 0.9999945
## COTR3-COTR2 -0.16148569 -1.4076520 1.0846807 0.9997504
## TR1-COTR2    0.47210021 -0.7740661 1.7182666 0.8816742
## TR2-COTR2    0.34529068 -0.9008757 1.5914570 0.9741729
## TR3-COTR2   -0.13758351 -1.3837499 1.1085828 0.9999140
## TR1-COTR3    0.63358590 -0.6125804 1.8797522 0.6522771
## TR2-COTR3    0.50677637 -0.7393900 1.7529427 0.8411266
## TR3-COTR3    0.02390218 -1.2222642 1.2700685 1.0000000
## TR2-TR1     -0.12680953 -1.3729759 1.1193568 0.9999503
## TR3-TR1     -0.60968372 -1.8558501 0.6364826 0.6910840
## TR3-TR2     -0.48287419 -1.7290405 0.7632922 0.8697442
```

```r
print("For glyphosate in TC")
```

```
## [1] "For glyphosate in TC"
```

```r
Propionate_G_TC <- Propionate[Propionate$Arm == 'glyphosate' & Propionate$Compartment == 'TC',]
res.aov <- aov(Value ~ Week, data = Propionate_G_TC)
summary(res.aov)
```

```
##             Df Sum Sq Mean Sq F value Pr(>F)
## Week         7  0.902  0.1289   0.618  0.734
## Residuals   16  3.338  0.2086
```

```r
TukeyHSD(res.aov, which = "Week")
```

```
##   Tukey multiple comparisons of means
##     95% family-wise confidence level
##
## Fit: aov(formula = Value ~ Week, data = Propionate_G_TC)
##
## $Week
##                    diff        lwr       upr     p adj
## C2-C1        0.31461558 -0.9765134 1.6057445 0.9873111
## COTR1-C1     0.37211812 -0.9190108 1.6632471 0.9681685
## COTR2-C1     0.40831129 -0.8828177 1.6994402 0.9488493
## COTR3-C1     0.32917130 -0.9619576 1.6203002 0.9836271
## TR1-C1       0.48028501 -0.8108439 1.7714139 0.8906858
## TR2-C1       0.73270568 -0.5584233 2.0238346 0.5312130
## TR3-C1       0.24885259 -1.0422763 1.5399815 0.9968405
## COTR1-C2     0.05750254 -1.2336264 1.3486315 0.9999998
## COTR2-C2     0.09369571 -1.1974332 1.3848246 0.9999950
## COTR3-C2     0.01455572 -1.2765732 1.3056847 1.0000000
## TR1-C2       0.16566943 -1.1254595 1.4567984 0.9997661
## TR2-C2       0.41809010 -0.8730388 1.7092190 0.9425276
## TR3-C2      -0.06576299 -1.3568919 1.2253659 0.9999996
## COTR2-COTR1  0.03619317 -1.2549358 1.3273221 1.0000000
## COTR3-COTR1 -0.04294681 -1.3340757 1.2481821 1.0000000
## TR1-COTR1    0.10816689 -1.1829620 1.3992958 0.9999867
## TR2-COTR1    0.36058756 -0.9305414 1.6517165 0.9730569
## TR3-COTR1   -0.12326552 -1.4143945 1.1678634 0.9999677
## COTR3-COTR2 -0.07913998 -1.3702689 1.2119890 0.9999984
## TR1-COTR2    0.07197372 -1.2191552 1.3631027 0.9999992
## TR2-COTR2    0.32439439 -0.9667345 1.6155233 0.9849139
## TR3-COTR2   -0.15945869 -1.4505876 1.1316702 0.9998184
## TR1-COTR3    0.15111371 -1.1400152 1.4422426 0.9998729
## TR2-COTR3    0.40353438 -0.8875946 1.6946633 0.9517622
## TR3-COTR3   -0.08031871 -1.3714476 1.2108102 0.9999983
## TR2-TR1      0.25242067 -1.0387083 1.5435496 0.9965525
## TR3-TR1     -0.23143242 -1.5225614 1.0596965 0.9979847
## TR3-TR2     -0.48385309 -1.7749820 0.8072758 0.8871001
```

```r
print("For Roundup in AC")
```

```
## [1] "For Roundup in AC"
```

```r
Propionate_R_AC <- Propionate[Propionate$Arm == 'roundup' & Propionate$Compartment == 'AC',]
res.aov <- aov(Value ~ Week, data = Propionate_R_AC)
summary(res.aov)
```

```
##             Df Sum Sq Mean Sq F value   Pr(>F)
## Week         7 16.698  2.3854    25.4 1.58e-07 ***
## Residuals   16  1.502  0.0939
## ---
## Signif. codes:  0 '***' 0.001 '**' 0.01 '*' 0.05 '.' 0.1 ' ' 1
```

```r
TukeyHSD(res.aov, which = "Week")
```

```
##   Tukey multiple comparisons of means
##     95% family-wise confidence level
```

```
##
## Fit: aov(formula = Value ~ Week, data = Propionate_R_AC)
##
## $Week
##                     diff        lwr         upr     p adj
## C2-C1       -0.362876215 -1.2290951  0.50334262 0.8213893
## COTR1-C1    -0.548485871 -1.4147047  0.31773296 0.4043241
## COTR2-C1    -0.286328159 -1.1525470  0.57989068 0.9364979
## COTR3-C1    -0.002566685 -0.8687855  0.86365215 1.0000000
## TR1-C1      -2.136903438 -3.0031223 -1.27068460 0.0000053
## TR2-C1      -2.061798146 -2.9280170 -1.19557931 0.0000084
## TR3-C1      -1.445822624 -2.3120415 -0.57960379 0.0005824
## COTR1-C2    -0.185609656 -1.0518285  0.68060918 0.9940020
## COTR2-C2     0.076548056 -0.7896708  0.94276689 0.9999809
## COTR3-C2     0.360309530 -0.5059093  1.22652837 0.8262616
## TR1-C2      -1.774027223 -2.6402461 -0.90780839 0.0000557
## TR2-C2      -1.698921931 -2.5651408 -0.83270310 0.0000936
## TR3-C2      -1.082946409 -1.9491652 -0.21672757 0.0094380
## COTR2-COTR1  0.262157712 -0.6040611  1.12837655 0.9590016
## COTR3-COTR1  0.545919186 -0.3202996  1.41213802 0.4097105
## TR1-COTR1   -1.588417567 -2.4546364 -0.72219873 0.0002050
## TR2-COTR1   -1.513312275 -2.3795311 -0.64709344 0.0003537
## TR3-COTR1   -0.897336753 -1.7635556 -0.03111792 0.0395579
## COTR3-COTR2  0.283761474 -0.5824574  1.14998031 0.9391999
## TR1-COTR2   -1.850575279 -2.7167941 -0.98435644 0.0000332
## TR2-COTR2   -1.775469987 -2.6416888 -0.90925115 0.0000552
## TR3-COTR2   -1.159494465 -2.0257133 -0.29327563 0.0051970
## TR1-COTR3   -2.134336753 -3.0005556 -1.26811792 0.0000054
## TR2-COTR3   -2.059231461 -2.9254503 -1.19301263 0.0000086
## TR3-COTR3   -1.443255940 -2.3094748 -0.57703710 0.0005936
## TR2-TR1      0.075105292 -0.7911135  0.94132413 0.9999832
## TR3-TR1      0.691080814 -0.1751380  1.55729965 0.1733363
## TR3-TR2      0.615975522 -0.2502433  1.48219436 0.2777020
```

```r
print("For Roundup in DC")
```

```
## [1] "For Roundup in DC"
```

```r
Propionate_R_DC <- Propionate[Propionate$Arm == 'roundup' & Propionate$Compartment == 'DC',]
res.aov <- aov(Value ~ Week, data = Propionate_R_DC)
summary(res.aov)
```

```
##             Df Sum Sq Mean Sq F value   Pr(>F)    
## Week         7 25.635   3.662   8.738 0.000182 ***
## Residuals   16  6.705   0.419                     
## ---
## Signif. codes:  0 '***' 0.001 '**' 0.01 '*' 0.05 '.' 0.1 ' ' 1
```

```r
TukeyHSD(res.aov, which = "Week")
```

```
##   Tukey multiple comparisons of means
##     95% family-wise confidence level
##
## Fit: aov(formula = Value ~ Week, data = Propionate_R_DC)
##
## $Week
```

```
##                   diff         lwr         upr     p adj
## C2-C1        0.59547836 -1.23453819  2.42549492 0.9411495
## COTR1-C1     0.92972814 -0.90028842  2.75974469 0.6530618
## COTR2-C1     1.54778308 -0.28223348  3.37779963 0.1310379
## COTR3-C1     2.66360705  0.83359050  4.49362361 0.0023704
## TR1-C1      -0.39194402 -2.22196057  1.43807253 0.9940188
## TR2-C1      -0.46707415 -2.29709070  1.36294241 0.9835262
## TR3-C1      -0.29598317 -2.12599972  1.53403339 0.9989472
## COTR1-C2     0.33424978 -1.49576678  2.16426633 0.9977342
## COTR2-C2     0.95230472 -0.87771184  2.78232127 0.6277897
## COTR3-C2     2.06812869  0.23811213  3.89814524 0.0211808
## TR1-C2      -0.98742238 -2.81743894  0.84259417 0.5882994
## TR2-C2      -1.06255251 -2.89256907  0.76746404 0.5048080
## TR3-C2      -0.89146153 -2.72147808  0.93855503 0.6953090
## COTR2-COTR1  0.61805494 -1.21196161  2.44807150 0.9296325
## COTR3-COTR1  1.73387891 -0.09613764  3.56389547 0.0700353
## TR1-COTR1   -1.32167216 -3.15168871  0.50834440 0.2621717
## TR2-COTR1   -1.39680229 -3.22681884  0.43321427 0.2103596
## TR3-COTR1   -1.22571130 -3.05572786  0.60430525 0.3411126
## COTR3-COTR2  1.11582397 -0.71419258  2.94584053 0.4479539
## TR1-COTR2   -1.93972710 -3.76974365 -0.10971054 0.0337737
## TR2-COTR2   -2.01485723 -3.84487378 -0.18484067 0.0257244
## TR3-COTR2   -1.84376624 -3.67378280 -0.01374969 0.0476203
## TR1-COTR3   -3.05555107 -4.88556763 -1.22553452 0.0005803
## TR2-COTR3   -3.13068120 -4.96069776 -1.30066465 0.0004457
## TR3-COTR3   -2.95959022 -4.78960677 -1.12957366 0.0008152
## TR2-TR1     -0.07513013 -1.90514668  1.75488643 0.9999999
## TR3-TR1      0.09596085 -1.73405570  1.92597741 0.9999995
## TR3-TR2      0.17109098 -1.65892557  2.00110754 0.9999720
```

```r
print("For Roundup in TC")
```

```
## [1] "For Roundup in TC"
```

```r
Propionate_R_TC <- Propionate[Propionate$Arm == 'roundup' & Propionate$Compartment == 'TC',]
res.aov <- aov(Value ~ Week, data = Propionate_R_TC)
summary(res.aov)
```

```
##             Df Sum Sq Mean Sq F value   Pr(>F)    
## Week         7 23.876   3.411   7.164 0.000569 ***
## Residuals   16  7.617   0.476                     
## ---
## Signif. codes:  0 '***' 0.001 '**' 0.01 '*' 0.05 '.' 0.1 ' ' 1
```

```r
TukeyHSD(res.aov, which = "Week")
```

```
##   Tukey multiple comparisons of means
##     95% family-wise confidence level
##
## Fit: aov(formula = Value ~ Week, data = Propionate_R_TC)
##
## $Week
##                  diff         lwr         upr     p adj
## C2-C1        0.7703347 -1.18016661  2.72083599 0.8589671
## COTR1-C1     1.2638162 -0.68668506  3.21431754 0.3780889
## COTR2-C1     1.5112921 -0.43920917  3.46179342 0.1973318
```

```
## COTR3-C1     1.9468123 -0.00368897  3.89731363 0.0506165
## TR1-C1      -0.7551275 -2.70562881  1.19537379 0.8702284
## TR2-C1      -0.9033621 -2.85386342  1.04713918 0.7422004
## TR3-C1      -0.1345780 -2.08507927  1.81592333 0.9999965
## COTR1-C2     0.4934816 -1.45701975  2.44398285 0.9843137
## COTR2-C2     0.7409574 -1.20954387  2.69145873 0.8802742
## COTR3-C2     1.1764776 -0.77402366  3.12697894 0.4605434
## TR1-C2      -1.5254622 -3.47596350  0.42503910 0.1894743
## TR2-C2      -1.6736968 -3.62419811  0.27680448 0.1217373
## TR3-C2      -0.9049127 -2.85541396  1.04558864 0.7406824
## COTR2-COTR1  0.2474759 -1.70302542  2.19797718 0.9997829
## COTR3-COTR1  0.6829961 -1.26750521  2.63349738 0.9166839
## TR1-COTR1   -2.0189438 -3.96944505 -0.06844246 0.0397749
## TR2-COTR1   -2.1671784 -4.11767966 -0.21667707 0.0240523
## TR3-COTR1   -1.3983942 -3.34889551  0.55210709 0.2694684
## COTR3-COTR2  0.4355202 -1.51498110  2.38602150 0.9923463
## TR1-COTR2   -2.2664196 -4.21692094 -0.31591834 0.0171071
## TR2-COTR2   -2.4146542 -4.36515555 -0.46415295 0.0102508
## TR3-COTR2   -1.6458701 -3.59637139  0.30463120 0.1325716
## TR1-COTR3   -2.7019398 -4.65244114 -0.75143854 0.0037953
## TR2-COTR3   -2.8501745 -4.80067575 -0.89967315 0.0022813
## TR3-COTR3   -2.0813903 -4.03189160 -0.13088900 0.0322145
## TR2-TR1     -0.1482346 -2.09873591  1.80226669 0.9999932
## TR3-TR1      0.6205495 -1.32995176  2.57105084 0.9473048
## TR3-TR2      0.7687842 -1.18171715  2.71928545 0.8601376
```

Butyrate

```
### Butyrate ####
Butyrate <- microbial_activity[microbial_activity$Compound == 'Butyrate',]

D <- ggplot(data = Butyrate, aes(x = Time, y = Value, colour = Time, fill = Time)) +
  geom_boxplot(alpha = 0.5, outlier.color = 'white') +
  stat_summary(aes(group = 1), geom = "point", fun = mean, shape = 17, size = 1) +
  stat_summary(aes(group = 1), geom = "line",  size = 0.2, fun = mean) +
  scale_x_discrete(breaks = c(1,2,3,4,5,6,7,8), labels = c("C", "C", "TR", "TR", "TR", "COTR", "COTR",
  scale_fill_manual(values = c("black", "black", "red", "red", "red", "blue", "blue", "blue")) +
  scale_colour_manual(values = c("black", "black", "red", "red", "red", "blue", "blue", "blue")) +
  ylab("Butyrate (mM)") +
  facet_grid(Compartment ~ Arm, scales = 'free') + theme_bw() +
  theme(axis.text.x = element_text(angle = 90, vjust = 0.5, hjust=1), text = element_text(size = 12)) +
  theme(legend.position = "none", axis.title.x=element_blank(), axis.text.x =element_blank()) + labs(ta

print("For glyphosate in AC")
```

```
## [1] "For glyphosate in AC"
```

```
Butyrate_G_AC <- Butyrate[Butyrate$Arm == 'glyphosate' & Butyrate$Compartment == 'AC',]
res.aov <- aov(Value ~ Week, data = Butyrate_G_AC)
summary(res.aov)
```

```
##             Df Sum Sq Mean Sq F value   Pr(>F)
## Week         7 11.355  1.6222   8.245 0.000256 ***
## Residuals   16  3.148  0.1967
## ---
```

```
## Signif. codes:  0 '***' 0.001 '**' 0.01 '*' 0.05 '.' 0.1 ' ' 1
```

```r
TukeyHSD(res.aov, which = "Week")
```

```
##   Tukey multiple comparisons of means
##     95% family-wise confidence level
##
## Fit: aov(formula = Value ~ Week, data = Butyrate_G_AC)
##
## $Week
##                    diff         lwr          upr     p adj
## C2-C1        0.51813143 -0.73573623  1.771999083 0.8306959
## COTR1-C1     1.77554443  0.52167678  3.029412089 0.0030864
## COTR2-C1     1.40505546  0.15118780  2.658923117 0.0225757
## COTR3-C1     1.67885408  0.42498643  2.932721740 0.0051840
## TR1-C1       0.22122260 -1.03264506  1.475090257 0.9981749
## TR2-C1       0.32612401 -0.92774364  1.579991668 0.9817043
## TR3-C1       0.14462214 -1.10924552  1.398489795 0.9998848
## COTR1-C2     1.25741301  0.00354535  2.511280662 0.0490915
## COTR2-C2     0.88692403 -0.36694362  2.140791690 0.2831014
## COTR3-C2     1.16072266 -0.09314500  2.414590313 0.0803340
## TR1-C2      -0.29690883 -1.55077648  0.956958830 0.9892292
## TR2-C2      -0.19200741 -1.44587507  1.061860241 0.9992585
## TR3-C2      -0.37350929 -1.62737694  0.880358368 0.9622032
## COTR2-COTR1 -0.37048897 -1.62435663  0.883378684 0.9637555
## COTR3-COTR1 -0.09669035 -1.35055801  1.157177307 0.9999925
## TR1-COTR1   -1.55432183 -2.80818949 -0.300454177 0.0101368
## TR2-COTR1   -1.44942042 -2.70328808 -0.195552765 0.0178116
## TR3-COTR1   -1.63092229 -2.88478995 -0.377054638 0.0067096
## COTR3-COTR2  0.27379862 -0.98006903  1.527666279 0.9932896
## TR1-COTR2   -1.18383286 -2.43770052  0.070034796 0.0715247
## TR2-COTR2   -1.07893145 -2.33279911  0.174936207 0.1199948
## TR3-COTR2   -1.26043332 -2.51430098 -0.006565666 0.0483299
## TR1-COTR3   -1.45763148 -2.71149914 -0.203763827 0.0170451
## TR2-COTR3   -1.35273007 -2.60659773 -0.098862416 0.0298052
## TR3-COTR3   -1.53423194 -2.78809960 -0.280364289 0.0112948
## TR2-TR1      0.10490141 -1.14896624  1.358769067 0.9999868
## TR3-TR1     -0.07660046 -1.33046812  1.177267194 0.9999985
## TR3-TR2     -0.18150187 -1.43536953  1.072365783 0.9994848
```

```r
print("For glyphosate in DC")
```

```
## [1] "For glyphosate in DC"
```

```r
Butyrate_G_DC <- Butyrate[Butyrate$Arm == 'glyphosate' & Butyrate$Compartment == 'DC',]
res.aov <- aov(Value ~ Week, data = Butyrate_G_DC)
summary(res.aov)
```

```
##             Df Sum Sq Mean Sq F value Pr(>F)
## Week         7  4.224  0.6035   3.471 0.0186 *
## Residuals   16  2.782  0.1739
## ---
## Signif. codes:  0 '***' 0.001 '**' 0.01 '*' 0.05 '.' 0.1 ' ' 1
```

```r
TukeyHSD(res.aov, which = "Week")
```

```
##   Tukey multiple comparisons of means
```

```
##     95% family-wise confidence level
##
## Fit: aov(formula = Value ~ Week, data = Butyrate_G_DC)
##
## $Week
##                   diff        lwr          upr     p adj
## C2-C1       -0.26620524 -1.4448721  0.912461654 0.9918156
## COTR1-C1     0.49528313 -0.6833838  1.673950021 0.8192550
## COTR2-C1     1.03237676 -0.1462901  2.211043654 0.1093140
## COTR3-C1     0.66535168 -0.5133152  1.844018572 0.5372893
## TR1-C1       0.39105504 -0.7876118  1.569721935 0.9353559
## TR2-C1      -0.15171589 -1.3303828  1.026951005 0.9997612
## TR3-C1       0.60757084 -0.5710961  1.786237730 0.6378626
## COTR1-C2     0.76148837 -0.4171785  1.940155257 0.3813876
## COTR2-C2     1.29858200  0.1199151  2.477248890 0.0255991
## COTR3-C2     0.93155692 -0.2471100  2.110223808 0.1808504
## TR1-C2       0.65726028 -0.5214066  1.835927171 0.5512639
## TR2-C2       0.11448935 -1.0641775  1.293156241 0.9999637
## TR3-C2       0.87377608 -0.3048908  2.052442965 0.2369743
## COTR2-COTR1  0.53709363 -0.6415733  1.715760523 0.7563152
## COTR3-COTR1  0.17006855 -1.0085983  1.348735441 0.9994954
## TR1-COTR1   -0.10422809 -1.2828950  1.074438804 0.9999808
## TR2-COTR1   -0.64699902 -1.8256659  0.531667874 0.5690791
## TR3-COTR1    0.11228771 -1.0663792  1.290954599 0.9999682
## COTR3-COTR2 -0.36702508 -1.5456920  0.811641808 0.9526473
## TR1-COTR2   -0.64132172 -1.8199886  0.537345171 0.5789687
## TR2-COTR2   -1.18409265 -2.3627595 -0.005425759 0.0485290
## TR3-COTR2   -0.42480592 -1.6034728  0.753860966 0.9049923
## TR1-COTR3   -0.27429664 -1.4529635  0.904370253 0.9902566
## TR2-COTR3   -0.81706757 -1.9957345  0.361599323 0.3040760
## TR3-COTR3   -0.05778084 -1.2364477  1.120886048 0.9999997
## TR2-TR1     -0.54277093 -1.7214378  0.635895960 0.7472369
## TR3-TR1      0.21651579 -0.9621511  1.395182685 0.9976522
## TR3-TR2      0.75928672 -0.4193802  1.937953615 0.3846720
```

```r
print("For glyphosate in TC")
```

```
## [1] "For glyphosate in TC"
```

```r
Butyrate_G_TC <- Butyrate[Butyrate$Arm == 'glyphosate' & Butyrate$Compartment == 'TC',]
res.aov <- aov(Value ~ Week, data = Butyrate_G_TC)
summary(res.aov)
```

```
##             Df Sum Sq Mean Sq F value   Pr(>F)
## Week         7  14.91  2.1297   8.291 0.000248 ***
## Residuals   16   4.11  0.2569
## ---
## Signif. codes:  0 '***' 0.001 '**' 0.01 '*' 0.05 '.' 0.1 ' ' 1
```

```r
TukeyHSD(res.aov, which = "Week")
```

```
##   Tukey multiple comparisons of means
##     95% family-wise confidence level
##
## Fit: aov(formula = Value ~ Week, data = Butyrate_G_TC)
##
```

```
## $Week
##                     diff         lwr         upr     p adj
## C2-C1         0.51922829 -0.91344575  1.95190233 0.9025953
## COTR1-C1      0.57363678 -0.85903726  2.00631082 0.8508016
## COTR2-C1      1.53518904  0.10251500  2.96786309 0.0312800
## COTR3-C1      1.34453989 -0.08813415  2.77721393 0.0741315
## TR1-C1       -0.34205361 -1.77472765  1.09062043 0.9887075
## TR2-C1       -0.78261764 -2.21529168  0.65005640 0.5745400
## TR3-C1       -0.44522812 -1.87790217  0.98744592 0.9531205
## COTR1-C2      0.05440849 -1.37826555  1.48708253 0.9999999
## COTR2-C2      1.01596075 -0.41671329  2.44863480 0.2805268
## COTR3-C2      0.82531160 -0.60736244  2.25798564 0.5139344
## TR1-C2       -0.86128190 -2.29395594  0.57139214 0.4643990
## TR2-C2       -1.30184593 -2.73451997  0.13082811 0.0893455
## TR3-C2       -0.96445641 -2.39713046  0.46821763 0.3355580
## COTR2-COTR1   0.96155226 -0.47112178  2.39422631 0.3388581
## COTR3-COTR1   0.77090311 -0.66177093  2.20357716 0.5913517
## TR1-COTR1    -0.91569039 -2.34836443  0.51698365 0.3936190
## TR2-COTR1    -1.35625442 -2.78892846  0.07641962 0.0703945
## TR3-COTR1    -1.01886490 -2.45153895  0.41380914 0.2776240
## COTR3-COTR2 -0.19064915 -1.62332319  1.24202489 0.9997027
## TR1-COTR2    -1.87724265 -3.30991669 -0.44456861 0.0062888
## TR2-COTR2    -2.31780668 -3.75048072 -0.88513264 0.0008122
## TR3-COTR2    -1.98041717 -3.41309121 -0.54774313 0.0038708
## TR1-COTR3    -1.68659350 -3.11926754 -0.25391946 0.0154382
## TR2-COTR3    -2.12715753 -3.55983157 -0.69448349 0.0019509
## TR3-COTR3    -1.78976802 -3.22244206 -0.35709398 0.0094987
## TR2-TR1      -0.44056403 -1.87323807  0.99211001 0.9555424
## TR3-TR1      -0.10317452 -1.53584856  1.32949952 0.9999953
## TR3-TR2       0.33738951 -1.09528453  1.77006355 0.9895648
```

```r
print("For Roundup in AC")
```

```
## [1] "For Roundup in AC"
```

```r
Butyrate_R_AC <- Butyrate[Butyrate$Arm == 'roundup' & Butyrate$Compartment == 'AC',]
res.aov <- aov(Value ~ Week, data = Butyrate_R_AC)
summary(res.aov)
```

```
##             Df Sum Sq Mean Sq F value Pr(>F)
## Week         7  4.674  0.6677   1.846  0.147
## Residuals   16  5.788  0.3617
```

```r
TukeyHSD(res.aov, which = "Week")
```

```
##   Tukey multiple comparisons of means
##     95% family-wise confidence level
##
## Fit: aov(formula = Value ~ Week, data = Butyrate_R_AC)
##
## $Week
##                     diff        lwr       upr     p adj
## C2-C1         1.46698214 -0.2332100 3.1671743 0.1183077
## COTR1-C1      1.09613472 -0.6040574 2.7963269 0.3837540
## COTR2-C1      1.22108807 -0.4791041 2.9212802 0.2677062
## COTR3-C1      0.62597764 -1.0742145 2.3261698 0.8955195
```

```
## TR1-C1       0.44603806 -1.2541541 2.1462302 0.9808128
## TR2-C1       0.99797662 -0.7022155 2.6981688 0.4921807
## TR3-C1       0.67557035 -1.0246218 2.3757625 0.8553914
## COTR1-C2    -0.37084743 -2.0710396 1.3293447 0.9933336
## COTR2-C2    -0.24589407 -1.9460862 1.4542981 0.9994877
## COTR3-C2    -0.84100450 -2.5411966 0.8591876 0.6802296
## TR1-C2      -1.02094409 -2.7211362 0.6792481 0.4657222
## TR2-C2      -0.46900552 -2.1691977 1.2311866 0.9747816
## TR3-C2      -0.79141180 -2.4916039 0.9087803 0.7377236
## COTR2-COTR1  0.12495335 -1.5752388 1.8251455 0.9999946
## COTR3-COTR1 -0.47015708 -2.1703492 1.2300351 0.9744472
## TR1-COTR1   -0.65009666 -2.3502888 1.0500955 0.8768858
## TR2-COTR1   -0.09815810 -1.7983502 1.6020340 0.9999990
## TR3-COTR1   -0.42056437 -2.1207565 1.2796278 0.9861826
## COTR3-COTR2 -0.59511043 -2.2953026 1.1050817 0.9168361
## TR1-COTR2   -0.77505001 -2.4752422 0.9251421 0.7559754
## TR2-COTR2   -0.22311145 -1.9233036 1.4770807 0.9997288
## TR3-COTR2   -0.54551772 -2.2457099 1.1546744 0.9450569
## TR1-COTR3   -0.17993958 -1.8801317 1.5202526 0.9999352
## TR2-COTR3    0.37199898 -1.3281932 2.0721911 0.9932101
## TR3-COTR3    0.04959271 -1.6505994 1.7497849 1.0000000
## TR2-TR1      0.55193856 -1.1482536 2.2521307 0.9418178
## TR3-TR1      0.22953229 -1.4706599 1.9297244 0.9996732
## TR3-TR2     -0.32240627 -2.0225984 1.3777859 0.9971416
```

```r
print("For Roundup in DC")
```

```
## [1] "For Roundup in DC"
```

```r
Butyrate_R_DC <- Butyrate[Butyrate$Arm == 'roundup' & Butyrate$Compartment == 'DC',]
res.aov <- aov(Value ~ Week, data = Butyrate_R_DC)
summary(res.aov)
```

```
##             Df Sum Sq Mean Sq F value  Pr(>F)
## Week         7 16.930  2.4186   5.438 0.00244 **
## Residuals   16  7.115  0.4447
## ---
## Signif. codes:  0 '***' 0.001 '**' 0.01 '*' 0.05 '.' 0.1 ' ' 1
```

```r
TukeyHSD(res.aov, which = "Week")
```

```
##   Tukey multiple comparisons of means
##     95% family-wise confidence level
##
## Fit: aov(formula = Value ~ Week, data = Butyrate_R_DC)
##
## $Week
##                    diff         lwr        upr     p adj
## C2-C1       -0.33752394 -2.22264400  1.5475961 0.9979989
## COTR1-C1    -2.05129113 -3.93641119 -0.1661711 0.0280208
## COTR2-C1    -2.00973856 -3.89485862 -0.1246185 0.0324279
## COTR3-C1    -2.33651422 -4.22163428 -0.4513942 0.0101485
## TR1-C1      -0.54531424 -2.43043430  1.3398058 0.9675486
## TR2-C1      -1.28277359 -3.16789365  0.6023465 0.3238659
## TR3-C1      -1.86091066 -3.74603072  0.0242094 0.0543294
## COTR1-C2    -1.71376718 -3.59888725  0.1713529 0.0891127
```

```
## COTR2-C2    -1.67221462 -3.55733468  0.2129054 0.1021015
## COTR3-C2    -1.99899028 -3.88411034 -0.1138702 0.0336726
## TR1-C2      -0.20779030 -2.09291036  1.6773298 0.9999149
## TR2-C2      -0.94524965 -2.83036971  0.9398704 0.6665295
## TR3-C2      -1.52338671 -3.40850677  0.3617333 0.1633970
## COTR2-COTR1  0.04155257 -1.84356749  1.9266726 1.0000000
## COTR3-COTR1 -0.28522309 -2.17034315  1.5998970 0.9993138
## TR1-COTR1    1.50597688 -0.37914318  3.3910969 0.1722880
## TR2-COTR1    0.76851753 -1.11660253  2.6536376 0.8395451
## TR3-COTR1    0.19038047 -1.69473959  2.0755005 0.9999528
## COTR3-COTR2 -0.32677566 -2.21189572  1.5583444 0.9983665
## TR1-COTR2    1.46442432 -0.42069574  3.3495444 0.1951308
## TR2-COTR2    0.72696497 -1.15815509  2.6120850 0.8723553
## TR3-COTR2    0.14882790 -1.73629216  2.0339480 0.9999912
## TR1-COTR3    1.79119998 -0.09392008  3.6763200 0.0688404
## TR2-COTR3    1.05374063 -0.83137943  2.9388607 0.5485146
## TR3-COTR3    0.47560356 -1.40951650  2.3607236 0.9845584
## TR2-TR1     -0.73745935 -2.62257941  1.1476607 0.8644328
## TR3-TR1     -1.31559641 -3.20071647  0.5695236 0.2970301
## TR3-TR2     -0.57813706 -2.46325712  1.3069830 0.9561440
```

```
print("For Roundup in TC")
```

```
## [1] "For Roundup in TC"
```

```
Butyrate_R_TC <- Butyrate[Butyrate$Arm == 'roundup' & Butyrate$Compartment == 'TC',]
res.aov <- aov(Value ~ Week, data = Butyrate_R_TC)
summary(res.aov)
```

```
##             Df Sum Sq Mean Sq F value  Pr(>F)
## Week         7  41.55   5.936   7.306 0.00051 ***
## Residuals   16  13.00   0.812
## ---
## Signif. codes:  0 '***' 0.001 '**' 0.01 '*' 0.05 '.' 0.1 ' ' 1
```

```
TukeyHSD(res.aov, which = "Week")
```

```
##   Tukey multiple comparisons of means
##     95% family-wise confidence level
##
## Fit: aov(formula = Value ~ Week, data = Butyrate_R_TC)
##
## $Week
##                    diff        lwr         upr     p adj
## C2-C1        0.09401942 -2.4539441  2.64198295 1.0000000
## COTR1-C1    -2.84532319 -5.3932867 -0.29735965 0.0231672
## COTR2-C1    -3.14586985 -5.6938334 -0.59790631 0.0104822
## COTR3-C1    -3.48889236 -6.0368559 -0.94092882 0.0042256
## TR1-C1      -1.78690443 -4.3348680  0.76105911 0.2919404
## TR2-C1      -2.58401940 -5.1319829 -0.03605586 0.0456076
## TR3-C1      -2.94644028 -5.4944038 -0.39847674 0.0177601
## COTR1-C2    -2.93934260 -5.4873061 -0.39137906 0.0180954
## COTR2-C2    -3.23988927 -5.7878528 -0.69192573 0.0081699
## COTR3-C2    -3.58291178 -6.1308753 -1.03494824 0.0032975
## TR1-C2      -1.88092385 -4.4288874  0.66703969 0.2409575
## TR2-C2      -2.67803882 -5.2260024 -0.13007528 0.0358147
```

```
## TR3-C2      -3.04045969 -5.5884232 -0.49249615 0.0138563
## COTR2-COTR1 -0.30054667 -2.8485102  2.24741687 0.9998662
## COTR3-COTR1 -0.64356917 -3.1915327  1.90439436 0.9844593
## TR1-COTR1    1.05841876 -1.4895448  3.60638230 0.8271735
## TR2-COTR1    0.26130379 -2.2866598  2.80926733 0.9999476
## TR3-COTR1   -0.10111709 -2.6490806  2.44684645 0.9999999
## COTR3-COTR2 -0.34302251 -2.8909860  2.20494103 0.9996792
## TR1-COTR2    1.35896542 -1.1889981  3.90692896 0.6010957
## TR2-COTR2    0.56185045 -1.9861131  3.10981399 0.9928901
## TR3-COTR2    0.19942958 -2.3485340  2.74739312 0.9999917
## TR1-COTR3    1.70198793 -0.8459756  4.24995147 0.3440756
## TR2-COTR3    0.90487296 -1.6430906  3.45283650 0.9111269
## TR3-COTR3    0.54245209 -2.0055115  3.09041563 0.9942287
## TR2-TR1     -0.79711497 -3.3450785  1.75084857 0.9515301
## TR3-TR1     -1.15953585 -3.7074994  1.38842769 0.7574299
## TR3-TR2     -0.36242088 -2.9103844  2.18554266 0.9995403
```

Valerate

```
### Valerate ####
Valerate <- microbial_activity[microbial_activity$Compound == 'Valerate',]

E <- ggplot(data = Valerate, aes(x = Time, y = Value, colour = Time, fill = Time)) +
  geom_boxplot(alpha = 0.5, outlier.color = 'white') +
  stat_summary(aes(group = 1), geom = "point", fun = mean, shape = 17, size = 1) +
  stat_summary(aes(group = 1), geom = "line",  size = 0.2, fun = mean) +
  scale_x_discrete(breaks = c(1,2,3,4,5,6,7,8), labels = c("C", "C", "TR", "TR", "TR", "COTR", "COTR",
  scale_fill_manual(values = c("black", "black", "red", "red", "red", "blue", "blue", "blue")) +
  scale_colour_manual(values = c("black", "black", "red", "red", "red", "blue", "blue", "blue")) +
  ylab("Valerate (mM)") +
  facet_grid(Compartment ~ Arm, scales = 'free') + theme_bw() +
  theme(axis.text.x = element_text(angle = 90, vjust = 0.5, hjust=1), text = element_text(size = 12)) +
  theme(legend.position = "none", axis.title.x=element_blank(), axis.text.x =element_blank()) + labs(ta

print("For glyphosate in AC")
```

```
## [1] "For glyphosate in AC"
```

```
Valerate_G_AC <- Valerate[Valerate$Arm == 'glyphosate' & Valerate$Compartment == 'AC',]
res.aov <- aov(Value ~ Week, data = Valerate_G_AC)
summary(res.aov)
```

```
##             Df Sum Sq Mean Sq F value   Pr(>F)
## Week         7  3.533  0.5048   7.823 0.000347 ***
## Residuals   16  1.032  0.0645
## ---
## Signif. codes:  0 '***' 0.001 '**' 0.01 '*' 0.05 '.' 0.1 ' ' 1
```

```
TukeyHSD(res.aov, which = "Week")
```

```
##   Tukey multiple comparisons of means
##     95% family-wise confidence level
##
## Fit: aov(formula = Value ~ Week, data = Valerate_G_AC)
##
## $Week
```

```
##                     diff        lwr         upr     p adj
## C2-C1       -0.413845132 -1.1318994  0.30420915 0.5133754
## COTR1-C1    -0.616502334 -1.3345566  0.10155195 0.1213808
## COTR2-C1    -0.745059891 -1.4631142 -0.02700560 0.0391230
## COTR3-C1    -0.700640001 -1.4186943  0.01741429 0.0584692
## TR1-C1      -0.746666960 -1.4647212 -0.02861267 0.0385525
## TR2-C1      -1.155128464 -1.8731828 -0.43707418 0.0008620
## TR3-C1      -1.323584157 -2.0416384 -0.60552987 0.0001931
## COTR1-C2    -0.202657202 -0.9207115  0.51539709 0.9715057
## COTR2-C2    -0.331214759 -1.0492690  0.38683953 0.7457729
## COTR3-C2    -0.286794869 -1.0048492  0.43125942 0.8523016
## TR1-C2      -0.332821828 -1.0508761  0.38523246 0.7415104
## TR2-C2      -0.741283332 -1.4593376 -0.02322904 0.0404955
## TR3-C2      -0.909739025 -1.6277933 -0.19168474 0.0084283
## COTR2-COTR1 -0.128557557 -0.8466118  0.58949673 0.9979996
## COTR3-COTR1 -0.084137668 -0.8021920  0.63391662 0.9998720
## TR1-COTR1   -0.130164627 -0.8482189  0.58788966 0.9978383
## TR2-COTR1   -0.538626130 -1.2566804  0.17942816 0.2260152
## TR3-COTR1   -0.707081824 -1.4251361  0.01097246 0.0551904
## COTR3-COTR2  0.044419890 -0.6736344  0.76247418 0.9999983
## TR1-COTR2   -0.001607069 -0.7196614  0.71644722 1.0000000
## TR2-COTR2   -0.410068573 -1.1281229  0.30798571 0.5239561
## TR3-COTR2   -0.578524266 -1.2965786  0.13953002 0.1656965
## TR1-COTR3   -0.046026959 -0.7640812  0.67202733 0.9999979
## TR2-COTR3   -0.454488462 -1.1725427  0.26356582 0.4047790
## TR3-COTR3   -0.622944156 -1.3409984  0.09511013 0.1149885
## TR2-TR1     -0.408461503 -1.1265158  0.30959278 0.5284763
## TR3-TR1     -0.576917197 -1.2949715  0.14113709 0.1678401
## TR3-TR2     -0.168455694 -0.8865100  0.54959859 0.9897919
```

```r
print("For glyphosate in DC")
```

```
## [1] "For glyphosate in DC"
```

```r
Valerate_G_DC <- Valerate[Valerate$Arm == 'glyphosate' & Valerate$Compartment == 'DC',]
res.aov <- aov(Value ~ Week, data = Valerate_G_DC)
summary(res.aov)
```

```
##             Df Sum Sq Mean Sq F value   Pr(>F)
## Week         7 2.9197  0.4171    9.88 8.74e-05 ***
## Residuals   16 0.6755  0.0422
## ---
## Signif. codes:  0 '***' 0.001 '**' 0.01 '*' 0.05 '.' 0.1 ' ' 1
```

```r
TukeyHSD(res.aov, which = "Week")
```

```
##   Tukey multiple comparisons of means
##     95% family-wise confidence level
##
## Fit: aov(formula = Value ~ Week, data = Valerate_G_DC)
##
## $Week
##                   diff         lwr          upr     p adj
## C2-C1       -0.23014289 -0.81097225  0.350686468 0.8570574
## COTR1-C1    -0.91829959 -1.49912894 -0.337470228 0.0010330
## COTR2-C1    -0.57737890 -1.15820825  0.003450461 0.0519606
```

```
## COTR3-C1    -0.83252417 -1.41335353 -0.251694811 0.0027490
## TR1-C1      -0.16150452 -0.74233388  0.419324837 0.9736816
## TR2-C1      -0.68755155 -1.26838091 -0.106722194 0.0147767
## TR3-C1      -0.97706051 -1.55788987 -0.396231154 0.0005354
## COTR1-C2    -0.68815670 -1.26898605 -0.107327338 0.0146734
## COTR2-C2    -0.34723601 -0.92806536  0.233593351 0.4708864
## COTR3-C2    -0.60238128 -1.18321064 -0.021551921 0.0392525
## TR1-C2       0.06863837 -0.51219099  0.649467727 0.9998645
## TR2-C2      -0.45740866 -1.03823802  0.123420695 0.1837766
## TR3-C2      -0.74691762 -1.32774698 -0.166088264 0.0074131
## COTR2-COTR1  0.34092069 -0.23990867  0.921750047 0.4922277
## COTR3-COTR1  0.08577542 -0.49505394  0.666604775 0.9994134
## TR1-COTR1    0.75679507  0.17596571  1.337624423 0.0066088
## TR2-COTR1    0.23074803 -0.35008132  0.811577391 0.8555051
## TR3-COTR1   -0.05876093 -0.63959028  0.522068432 0.9999522
## COTR3-COTR2 -0.25514527 -0.83597463  0.325684085 0.7863386
## TR1-COTR2    0.41587438 -0.16495498  0.996703733 0.2707833
## TR2-COTR2   -0.11017266 -0.69100201  0.470656702 0.9971367
## TR3-COTR2   -0.39968161 -0.98051097  0.181147743 0.3118839
## TR1-COTR3    0.67101965  0.09019029  1.251849006 0.0178932
## TR2-COTR3    0.14497262 -0.43585674  0.725801974 0.9854637
## TR3-COTR3   -0.14453634 -0.72536570  0.436293015 0.9857086
## TR2-TR1     -0.52604703 -1.10687639  0.054782326 0.0910247
## TR3-TR1     -0.81555599 -1.39638535 -0.234726633 0.0033435
## TR3-TR2     -0.28950896 -0.87033832  0.291320398 0.6725808
```

```
print("For glyphosate in TC")
```

```
## [1] "For glyphosate in TC"
```

```
Valerate_G_TC <- Valerate[Valerate$Arm == 'glyphosate' & Valerate$Compartment == 'TC',]
res.aov <- aov(Value ~ Week, data = Valerate_G_TC)
summary(res.aov)
```

```
##             Df Sum Sq Mean Sq F value Pr(>F)
## Week         7  2.646  0.3780    2.26 0.0838 .
## Residuals   16  2.676  0.1672
## ---
## Signif. codes:  0 '***' 0.001 '**' 0.01 '*' 0.05 '.' 0.1 ' ' 1
```

```
TukeyHSD(res.aov, which = "Week")
```

```
##   Tukey multiple comparisons of means
##     95% family-wise confidence level
##
## Fit: aov(formula = Value ~ Week, data = Valerate_G_TC)
##
## $Week
##                    diff        lwr        upr     p adj
## C2-C1        0.25075107 -0.9052390 1.40674112 0.9935492
## COTR1-C1    -0.49735634 -1.6533464 0.65863371 0.8022211
## COTR2-C1    -0.42617556 -1.5821656 0.72981449 0.8949087
## COTR3-C1    -0.53654773 -1.6925378 0.61944232 0.7402864
## TR1-C1      -0.01885545 -1.1748455 1.13713461 1.0000000
## TR2-C1      -0.37160217 -1.5275922 0.78438788 0.9445492
## TR3-C1      -0.84208825 -1.9980783 0.31390180 0.2537362
```

```
## COTR1-C2    -0.74810741 -1.9040975 0.40788264 0.3794639
## COTR2-C2    -0.67692663 -1.8329167 0.47906342 0.4949477
## COTR3-C2    -0.78729880 -1.9432889 0.36869125 0.3229358
## TR1-C2      -0.26960651 -1.4255966 0.88638354 0.9901328
## TR2-C2      -0.62235324 -1.7783433 0.53363681 0.5907639
## TR3-C2      -1.09283932 -2.2488294 0.06315073 0.0709716
## COTR2-COTR1  0.07118078 -1.0848093 1.22717084 0.9999984
## COTR3-COTR1 -0.03919139 -1.1951814 1.11679866 1.0000000
## TR1-COTR1    0.47850090 -0.6774892 1.63449095 0.8295557
## TR2-COTR1    0.12575417 -1.0302359 1.28174422 0.9999221
## TR3-COTR1   -0.34473191 -1.5007220 0.81125814 0.9619884
## COTR3-COTR2 -0.11037217 -1.2663622 1.04561788 0.9999677
## TR1-COTR2    0.40732011 -0.7486699 1.56331017 0.9142607
## TR2-COTR2    0.05457339 -1.1014167 1.21056344 0.9999997
## TR3-COTR2   -0.41591269 -1.5719027 0.74007736 0.9057293
## TR1-COTR3    0.51769229 -0.6382978 1.67368234 0.7708547
## TR2-COTR3    0.16494556 -0.9910445 1.32093561 0.9995308
## TR3-COTR3   -0.30554052 -1.4615306 0.85044953 0.9800089
## TR2-TR1     -0.35274673 -1.5087368 0.80324332 0.9572475
## TR3-TR1     -0.82323281 -1.9792229 0.33275725 0.2762253
## TR3-TR2     -0.47048608 -1.6264761 0.68550397 0.8406092
```

```r
print("For Roundup in AC")
```

```
## [1] "For Roundup in AC"
```

```r
Valerate_R_AC <- Valerate[Valerate$Arm == 'roundup' & Valerate$Compartment == 'AC',]
res.aov <- aov(Value ~ Week, data = Valerate_R_AC)
summary(res.aov)
```

```
##             Df Sum Sq Mean Sq F value   Pr(>F)    
## Week         7  68.32   9.760   22.81 3.41e-07 ***
## Residuals   16   6.85   0.428                     
## ---
## Signif. codes:  0 '***' 0.001 '**' 0.01 '*' 0.05 '.' 0.1 ' ' 1
```

```r
TukeyHSD(res.aov, which = "Week")
```

```
##   Tukey multiple comparisons of means
##     95% family-wise confidence level
## 
## Fit: aov(formula = Value ~ Week, data = Valerate_R_AC)
## 
## $Week
##                   diff       lwr         upr     p adj
## C2-C1       -0.5694628 -2.418580  1.27965451 0.9552106
## COTR1-C1    -3.2541939 -5.103311 -1.40507655 0.0003260
## COTR2-C1    -2.7767424 -4.625860 -0.92762504 0.0017436
## COTR3-C1    -2.8900564 -4.739174 -1.04093911 0.0011632
## TR1-C1      -4.2194431 -6.068560 -2.37032578 0.0000145
## TR2-C1      -5.0744688 -6.923586 -3.22535146 0.0000013
## TR3-C1      -4.4236706 -6.272788 -2.57455327 0.0000079
## COTR1-C2    -2.6847311 -4.533848 -0.83561374 0.0024283
## COTR2-C2    -2.2072795 -4.056397 -0.35816223 0.0138187
## COTR3-C2    -2.3205936 -4.169711 -0.47147630 0.0091385
## TR1-C2      -3.6499803 -5.499098 -1.80086297 0.0000868
```

```
## TR2-C2      -4.5050060 -6.354123 -2.65588865 0.0000062
## TR3-C2      -3.8542078 -5.703325 -2.00509046 0.0000450
## COTR2-COTR1  0.4774515 -1.371666  2.32656883 0.9824286
## COTR3-COTR1  0.3641374 -1.484980  2.21325476 0.9963964
## TR1-COTR1   -0.9652492 -2.814367  0.88386809 0.6244500
## TR2-COTR1   -1.8202749 -3.669392  0.02884241 0.0553033
## TR3-COTR1   -1.1694767 -3.018594  0.67964061 0.4056733
## COTR3-COTR2 -0.1133141 -1.962431  1.73580324 0.9999985
## TR1-COTR2   -1.4427007 -3.291818  0.40641658 0.1914795
## TR2-COTR2   -2.2977264 -4.146844 -0.44860911 0.0099345
## TR3-COTR2   -1.6469282 -3.496046  0.20218909 0.0998725
## TR1-COTR3   -1.3293867 -3.178504  0.51973065 0.2667007
## TR2-COTR3   -2.1844124 -4.033530 -0.33529503 0.0150188
## TR3-COTR3   -1.5336142 -3.382731  0.31550317 0.1443808
## TR2-TR1     -0.8550257 -2.704143  0.99409163 0.7436242
## TR3-TR1     -0.2042275 -2.053345  1.64488983 0.9999138
## TR3-TR2      0.6507982 -1.198319  2.49991552 0.9147120
```

```
print("For Roundup in DC")
```

```
## [1] "For Roundup in DC"
```

```
Valerate_R_DC <- Valerate[Valerate$Arm == 'roundup' & Valerate$Compartment == 'DC',]
res.aov <- aov(Value ~ Week, data = Valerate_R_DC)
summary(res.aov)
```

```
##             Df Sum Sq Mean Sq F value  Pr(>F)
## Week         7  74.01  10.573   14.96 6.2e-06 ***
## Residuals   16  11.31   0.707
## ---
## Signif. codes:  0 '***' 0.001 '**' 0.01 '*' 0.05 '.' 0.1 ' ' 1
```

```
TukeyHSD(res.aov, which = "Week")
```

```
##   Tukey multiple comparisons of means
##     95% family-wise confidence level
##
## Fit: aov(formula = Value ~ Week, data = Valerate_R_DC)
##
## $Week
##                    diff        lwr         upr     p adj
## C2-C1       -0.08704461 -2.4639067  2.28981751 1.0000000
## COTR1-C1    -3.84543118 -6.2222933 -1.46856906 0.0008120
## COTR2-C1    -3.13128088 -5.5081430 -0.75441876 0.0059948
## COTR3-C1    -3.07338662 -5.4502487 -0.69652451 0.0070663
## TR1-C1      -2.40100678 -4.7778689 -0.02414467 0.0468084
## TR2-C1      -4.99470938 -7.3715715 -2.61784727 0.0000407
## TR3-C1      -4.59603384 -6.9728960 -2.21917172 0.0001107
## COTR1-C2    -3.75838657 -6.1352487 -1.38152445 0.0010315
## COTR2-C2    -3.04423627 -5.4210984 -0.66737415 0.0076766
## COTR3-C2    -2.98634202 -5.3632041 -0.60947990 0.0090495
## TR1-C2      -2.31396217 -4.6908243  0.06289994 0.0593050
## TR2-C2      -4.90766477 -7.2845269 -2.53080266 0.0000505
## TR3-C2      -4.50898923 -6.8858513 -2.13212711 0.0001385
## COTR2-COTR1  0.71415030 -1.6627118  3.09101241 0.9604946
## COTR3-COTR1  0.77204455 -1.6048176  3.14890667 0.9416572
```

```
## TR1-COTR1    1.44442439 -0.9324377  3.82128651 0.4518330
## TR2-COTR1   -1.14927821 -3.5261403  1.22758391 0.7024837
## TR3-COTR1   -0.75060266 -3.1274648  1.62625945 0.9492081
## COTR3-COTR2  0.05789425 -2.3189679  2.43475637 1.0000000
## TR1-COTR2    0.73027410 -1.6465880  3.10713621 0.9557384
## TR2-COTR2   -1.86342851 -4.2402906  0.51343361 0.1874621
## TR3-COTR2   -1.46475296 -3.8416151  0.91210915 0.4356098
## TR1-COTR3    0.67237984 -1.7044823  3.04924196 0.9711532
## TR2-COTR3   -1.92132276 -4.2981849  0.45553936 0.1631777
## TR3-COTR3   -1.52264721 -3.8995093  0.85421490 0.3910090
## TR2-TR1     -2.59370260 -4.9705647 -0.21684049 0.0274530
## TR3-TR1     -2.19502706 -4.5718892  0.18183506 0.0814576
## TR3-TR2      0.39867554 -1.9781866  2.77553766 0.9986731
```

```r
print("For Roundup in TC")
```

```
## [1] "For Roundup in TC"
```

```r
Valerate_R_TC <- Valerate[Valerate$Arm == 'roundup' & Valerate$Compartment == 'TC',]
res.aov <- aov(Value ~ Week, data = Valerate_R_TC)
summary(res.aov)
```

```
##             Df Sum Sq Mean Sq F value   Pr(>F)    
## Week         7  71.39   10.20   15.46 4.97e-06 ***
## Residuals   16  10.55    0.66                     
## ---
## Signif. codes:  0 '***' 0.001 '**' 0.01 '*' 0.05 '.' 0.1 ' ' 1
```

```r
TukeyHSD(res.aov, which = "Week")
```

```
##   Tukey multiple comparisons of means
##     95% family-wise confidence level
## 
## Fit: aov(formula = Value ~ Week, data = Valerate_R_TC)
## 
## $Week
##                    diff       lwr        upr     p adj
## C2-C1       -0.39487614 -2.690815  1.9010631 0.9984453
## COTR1-C1    -3.76883299 -6.064772 -1.4728937 0.0006962
## COTR2-C1    -3.13357231 -5.429512 -0.8376330 0.0043542
## COTR3-C1    -3.25417406 -5.550113 -0.9582348 0.0030595
## TR1-C1      -3.34128624 -5.637226 -1.0453470 0.0023737
## TR2-C1      -5.11295506 -7.408894 -2.8170158 0.0000198
## TR3-C1      -4.66408676 -6.960026 -2.3681475 0.0000615
## COTR1-C2    -3.37395685 -5.669896 -1.0780176 0.0021588
## COTR2-C2    -2.73869617 -5.034635 -0.4427569 0.0138980
## COTR3-C2    -2.85929792 -5.155237 -0.5633586 0.0097508
## TR1-C2      -2.94641010 -5.242349 -0.6504708 0.0075463
## TR2-C2      -4.71807892 -7.014018 -2.4221396 0.0000535
## TR3-C2      -4.26921061 -6.565150 -1.9732713 0.0001747
## COTR2-COTR1  0.63526068 -1.660679  2.9312000 0.9743692
## COTR3-COTR1  0.51465893 -1.781280  2.8105982 0.9921686
## TR1-COTR1    0.42754675 -1.868393  2.7234860 0.9974449
## TR2-COTR1   -1.34412207 -3.640061  0.9518172 0.4952395
## TR3-COTR1   -0.89525376 -3.191193  1.4006855 0.8662638
## COTR3-COTR2 -0.12060175 -2.416541  2.1753375 0.9999995
```

```
## TR1-COTR2   -0.20771393 -2.503653  2.0882254 0.9999776
## TR2-COTR2   -1.97938275 -4.275322  0.3165565 0.1188150
## TR3-COTR2   -1.53051444 -3.826454  0.7654248 0.3463255
## TR1-COTR3   -0.08711218 -2.383051  2.2088271 0.9999999
## TR2-COTR3   -1.85878100 -4.154720  0.4371583 0.1620049
## TR3-COTR3   -1.40991269 -3.705852  0.8860266 0.4396911
## TR2-TR1     -1.77166882 -4.067608  0.5242705 0.2008407
## TR3-TR1     -1.32280052 -3.618740  0.9731388 0.5137657
## TR3-TR2      0.44886831 -1.847071  2.7448076 0.9965523
```

Caproate

```
### Caproate ####
Caproate <- microbial_activity[microbial_activity$Compound == 'Caproate',]

F <- ggplot(data = Caproate, aes(x = Time, y = Value, colour = Time, fill = Time)) +
  geom_boxplot(alpha = 0.5, outlier.color = 'white') +
  stat_summary(aes(group = 1), geom = "point", fun = mean, shape = 17, size = 1) +
  stat_summary(aes(group = 1), geom = "line",  size = 0.2, fun = mean) +
  scale_x_discrete(breaks = c(1,2,3,4,5,6,7,8), labels = c("C", "C", "TR", "TR", "TR", "COTR", "COTR",
  scale_fill_manual(values = c("black", "black", "red", "red", "red", "blue", "blue", "blue")) +
  scale_colour_manual(values = c("black", "black", "red", "red", "red", "blue", "blue", "blue")) +
  ylab("Caproate (mM)") +
  facet_grid(Compartment ~ Arm, scales = 'free') + theme_bw() +
  theme(axis.text.x = element_text(angle = 90, vjust = 0.5, hjust=1), text = element_text(size = 12)) +
  theme(legend.position = "none", axis.title.x=element_blank(), axis.text.x =element_blank()) + labs(ta

print("For glyphosate in AC")
```

```
## [1] "For glyphosate in AC"
```

```
Caproate_G_AC <- Caproate[Caproate$Arm == 'glyphosate' & Caproate$Compartment == 'AC',]
res.aov <- aov(Value ~ Week, data = Caproate_G_AC)
summary(res.aov)
```

```
##             Df Sum Sq Mean Sq F value  Pr(>F)
## Week         7 1.3486 0.19266   4.737 0.00478 **
## Residuals   16 0.6507 0.04067
## ---
## Signif. codes:  0 '***' 0.001 '**' 0.01 '*' 0.05 '.' 0.1 ' ' 1
```

```
TukeyHSD(res.aov, which = "Week")
```

```
##   Tukey multiple comparisons of means
##     95% family-wise confidence level
##
## Fit: aov(formula = Value ~ Week, data = Caproate_G_AC)
##
## $Week
##                   diff        lwr         upr     p adj
## C2-C1       0.01759728 -0.5524792  0.58767373 1.0000000
## COTR1-C1   -0.17989928 -0.7499757  0.39017718 0.9493878
## COTR2-C1   -0.49212471 -1.0622012  0.07795174 0.1180029
## COTR3-C1   -0.47938128 -1.0494577  0.09069517 0.1348831
## TR1-C1     -0.28901125 -0.8590877  0.28106520 0.6552534
## TR2-C1     -0.44572013 -1.0157966  0.12435632 0.1897159
```

```
## TR3-C1       -0.69649240 -1.2665688 -0.12641594 0.0114364
## COTR1-C2     -0.19749656 -0.7675730  0.37257990 0.9207064
## COTR2-C2     -0.50972199 -1.0797984  0.06035446 0.0977616
## COTR3-C2     -0.49697856 -1.0670550  0.07309789 0.1120784
## TR1-C2       -0.30660853 -0.8766850  0.26346792 0.5918648
## TR2-C2       -0.46331741 -1.0333939  0.10675905 0.1590977
## TR3-C2       -0.71408968 -1.2841661 -0.14401322 0.0092849
## COTR2-COTR1 -0.31222544 -0.8823019  0.25785102 0.5716108
## COTR3-COTR1 -0.29948201 -0.8695585  0.27059444 0.6175990
## TR1-COTR1   -0.10911197 -0.6791884  0.46096448 0.9969733
## TR2-COTR1   -0.26582085 -0.8358973  0.30425560 0.7361761
## TR3-COTR1   -0.51659312 -1.0866696  0.05348333 0.0907431
## COTR3-COTR2  0.01274343 -0.5573330  0.58281988 1.0000000
## TR1-COTR2    0.20311346 -0.3669630  0.77318992 0.9098038
## TR2-COTR2    0.04640459 -0.5236719  0.61648104 0.9999891
## TR3-COTR2   -0.20436768 -0.7744441  0.36570877 0.9072532
## TR1-COTR3    0.19037004 -0.3797064  0.76044649 0.9333151
## TR2-COTR3    0.03366116 -0.5364153  0.60373761 0.9999988
## TR3-COTR3   -0.21711111 -0.7871876  0.35296534 0.8789649
## TR2-TR1     -0.15670888 -0.7267853  0.41336757 0.9752519
## TR3-TR1     -0.40748115 -0.9775576  0.16259531 0.2724934
## TR3-TR2     -0.25077227 -0.8208487  0.31930418 0.7852399
```

```r
print("For glyphosate in DC")
```

```
## [1] "For glyphosate in DC"
```

```r
Caproate_G_DC <- Caproate[Caproate$Arm == 'glyphosate' & Caproate$Compartment == 'DC',]
res.aov <- aov(Value ~ Week, data = Caproate_G_DC)
summary(res.aov)
```

```
##             Df Sum Sq Mean Sq F value   Pr(>F)
## Week         7  6.542  0.9346   9.962 8.31e-05 ***
## Residuals   16  1.501  0.0938
## ---
## Signif. codes:  0 '***' 0.001 '**' 0.01 '*' 0.05 '.' 0.1 ' ' 1
```

```r
TukeyHSD(res.aov, which = "Week")
```

```
##   Tukey multiple comparisons of means
##     95% family-wise confidence level
##
## Fit: aov(formula = Value ~ Week, data = Caproate_G_DC)
##
## $Week
##                   diff        lwr         upr     p adj
## C2-C1       -0.0482405 -0.9140983  0.81761734 0.9999992
## COTR1-C1    -0.2446359 -1.1104937  0.62122194 0.9713419
## COTR2-C1     0.5207901 -0.3450677  1.38664799 0.4638144
## COTR3-C1     0.2704462 -0.5954116  1.13630404 0.9519156
## TR1-C1      -0.5338679 -1.3997258  0.33198990 0.4350045
## TR2-C1      -0.7064934 -1.5723512  0.15936447 0.1561583
## TR3-C1      -1.2112066 -2.0770644 -0.34534874 0.0034639
## COTR1-C2    -0.1963954 -1.0622532  0.66946244 0.9916080
## COTR2-C2     0.5690306 -0.2968272  1.43488849 0.3621578
## COTR3-C2     0.3186867 -0.5471711  1.18454454 0.8956718
```

```
## TR1-C2      -0.4856274 -1.3514853  0.38023040 0.5446742
## TR2-C2      -0.6582529 -1.5241107  0.20760497 0.2139009
## TR3-C2      -1.1629661 -2.0288239 -0.29710824 0.0050394
## COTR2-COTR1  0.7654261 -0.1004318  1.63128389 0.1040274
## COTR3-COTR1  0.5150821 -0.3507757  1.38093994 0.4766358
## TR1-COTR1   -0.2892320 -1.1550899  0.57662581 0.9332165
## TR2-COTR1   -0.4618575 -1.3277153  0.40400038 0.6009790
## TR3-COTR1   -0.9665707 -1.8324285 -0.10071283 0.0232275
## COTR3-COTR2 -0.2503440 -1.1162018  0.61551389 0.9676336
## TR1-COTR2   -1.0546581 -1.9205159 -0.18880024 0.0117258
## TR2-COTR2   -1.2272835 -2.0931414 -0.36142567 0.0030583
## TR3-COTR2   -1.7319967 -2.5978546 -0.86613888 0.0000740
## TR1-COTR3   -0.8043141 -1.6701720  0.06154371 0.0787326
## TR2-COTR3   -0.9769396 -1.8427974 -0.11108172 0.0214409
## TR3-COTR3   -1.4816528 -2.3475106 -0.61579493 0.0004444
## TR2-TR1     -0.1726254 -1.0384833  0.69323241 0.9961150
## TR3-TR1     -0.6773386 -1.5431965  0.18851920 0.1892750
## TR3-TR2     -0.5047132 -1.3705711  0.36114463 0.5002631
```

```r
print("For glyphosate in TC")
```

```
## [1] "For glyphosate in TC"
```

```r
Caproate_G_TC <- Caproate[Caproate$Arm == 'glyphosate' & Caproate$Compartment == 'TC',]
res.aov <- aov(Value ~ Week, data = Caproate_G_TC)
summary(res.aov)
```

```
##             Df Sum Sq Mean Sq F value Pr(>F)
## Week         7 1.1362  0.1623   3.697 0.0144 *
## Residuals   16 0.7024  0.0439
## ---
## Signif. codes:  0 '***' 0.001 '**' 0.01 '*' 0.05 '.' 0.1 ' ' 1
```

```r
TukeyHSD(res.aov, which = "Week")
```

```
##   Tukey multiple comparisons of means
##     95% family-wise confidence level
##
## Fit: aov(formula = Value ~ Week, data = Caproate_G_TC)
##
## $Week
##                     diff        lwr         upr     p adj
## C2-C1        0.210680952 -0.3816234  0.80298530 0.9104858
## COTR1-C1    -0.240298898 -0.8326033  0.35200545 0.8426370
## COTR2-C1    -0.203674816 -0.7959792  0.38862954 0.9234104
## COTR3-C1    -0.393314595 -0.9856189  0.19898976 0.3506079
## TR1-C1      -0.174270690 -0.7665750  0.41803366 0.9645446
## TR2-C1      -0.394337322 -0.9866417  0.19796703 0.3477345
## TR3-C1      -0.506503099 -1.0988075  0.08580125 0.1239086
## COTR1-C2    -0.450979850 -1.0432842  0.14132450 0.2125366
## COTR2-C2    -0.414355768 -1.0066601  0.17794858 0.2945224
## COTR3-C2    -0.603995546 -1.1962999 -0.01169119 0.0439761
## TR1-C2      -0.384951641 -0.9772560  0.20735271 0.3746462
## TR2-C2      -0.605018274 -1.1973226 -0.01271392 0.0434833
## TR3-C2      -0.717184051 -1.3094884 -0.12487970 0.0123105
## COTR2-COTR1  0.036624082 -0.5556803  0.62892843 0.9999984
```

```
## COTR3-COTR1 -0.153015697 -0.7453200  0.43928866 0.9823777
## TR1-COTR1    0.066028208 -0.5262761  0.65833256 0.9999082
## TR2-COTR1   -0.154038424 -0.7463428  0.43826593 0.9817152
## TR3-COTR1   -0.266204201 -0.8585086  0.32610015 0.7679082
## COTR3-COTR2 -0.189639778 -0.7819441  0.40266457 0.9456303
## TR1-COTR2    0.029404126 -0.5629002  0.62170848 0.9999996
## TR2-COTR2   -0.190662506 -0.7829669  0.40164185 0.9441744
## TR3-COTR2   -0.302828283 -0.8951326  0.28947607 0.6464669
## TR1-COTR3    0.219043905 -0.3732604  0.81134826 0.8934581
## TR2-COTR3   -0.001022727 -0.5933271  0.59128163 1.0000000
## TR3-COTR3   -0.113188504 -0.7054929  0.47911585 0.9970024
## TR2-TR1     -0.220066632 -0.8123710  0.37223772 0.8912574
## TR3-TR1     -0.332232409 -0.9245368  0.26007194 0.5445679
## TR3-TR2     -0.112165777 -0.7044701  0.48013858 0.9971654
```

```
print("For Roundup in AC")
```

```
## [1] "For Roundup in AC"
```

```
Caproate_R_AC <- Caproate[Caproate$Arm == 'roundup' & Caproate$Compartment == 'AC',]
res.aov <- aov(Value ~ Week, data = Caproate_R_AC)
summary(res.aov)
```

```
##             Df Sum Sq Mean Sq F value  Pr(>F)
## Week         7 12.134  1.7334    7.14 0.00058 ***
## Residuals   16  3.884  0.2428
## ---
## Signif. codes:  0 '***' 0.001 '**' 0.01 '*' 0.05 '.' 0.1 ' ' 1
```

```
TukeyHSD(res.aov, which = "Week")
```

```
##   Tukey multiple comparisons of means
##     95% family-wise confidence level
##
## Fit: aov(formula = Value ~ Week, data = Caproate_R_AC)
##
## $Week
##                    diff        lwr         upr     p adj
## C2-C1        0.25007392 -1.1427345  1.64288232 0.9979637
## COTR1-C1    -1.46043348 -2.8532419 -0.06762509 0.0364084
## COTR2-C1    -1.29186694 -2.6846753  0.10094146 0.0794254
## COTR3-C1    -1.72697414 -3.1197825 -0.33416575 0.0101163
## TR1-C1      -0.74332487 -2.1361333  0.64948353 0.6004075
## TR2-C1      -1.58343035 -2.9762388 -0.19062196 0.0202454
## TR3-C1      -1.50034498 -2.8931534 -0.10753659 0.0301305
## COTR1-C2    -1.71050740 -3.1033158 -0.31769901 0.0109569
## COTR2-C2    -1.54194086 -2.9347493 -0.14913246 0.0247050
## COTR3-C2    -1.97704807 -3.3698565 -0.58423967 0.0030166
## TR1-C2      -0.99339879 -2.3862072  0.39940961 0.2746802
## TR2-C2      -1.83350428 -3.2263127 -0.44069588 0.0060351
## TR3-C2      -1.75041890 -3.1432273 -0.35761051 0.0090292
## COTR2-COTR1  0.16856655 -1.2242419  1.56137494 0.9998412
## COTR3-COTR1 -0.26654066 -1.6593491  1.12626773 0.9969762
## TR1-COTR1    0.71710861 -0.6756998  2.10991701 0.6391083
## TR2-COTR1   -0.12299687 -1.5158053  1.26981152 0.9999810
## TR3-COTR1   -0.03991150 -1.4327199  1.35289689 1.0000000
```

```
## COTR3-COTR2 -0.43510721 -1.8279156  0.95770119 0.9518765
## TR1-COTR2    0.54854207 -0.8442663  1.94135046 0.8605900
## TR2-COTR2   -0.29156342 -1.6843718  1.10124498 0.9947830
## TR3-COTR2   -0.20847805 -1.6012864  1.18433035 0.9993599
## TR1-COTR3    0.98364928 -0.4091591  2.37645767 0.2847194
## TR2-COTR3    0.14354379 -1.2492646  1.53635219 0.9999458
## TR3-COTR3    0.22662916 -1.1661792  1.61943756 0.9989060
## TR2-TR1     -0.84010549 -2.2329139  0.55270291 0.4605299
## TR3-TR1     -0.75702012 -2.1498285  0.63578828 0.5801752
## TR3-TR2      0.08308537 -1.3097230  1.47589377 0.9999987
```

```r
print("For Roundup in DC")
```

```
## [1] "For Roundup in DC"
```

```r
Caproate_R_DC <- Caproate[Caproate$Arm == 'roundup' & Caproate$Compartment == 'DC',]
res.aov <- aov(Value ~ Week, data = Caproate_R_DC)
summary(res.aov)
```

```
##             Df Sum Sq Mean Sq F value   Pr(>F)    
## Week         7  52.70   7.529   25.24 1.66e-07 ***
## Residuals   16   4.77   0.298                     
## ---
## Signif. codes:  0 '***' 0.001 '**' 0.01 '*' 0.05 '.' 0.1 ' ' 1
```

```r
TukeyHSD(res.aov, which = "Week")
```

```
##   Tukey multiple comparisons of means
##     95% family-wise confidence level
## 
## Fit: aov(formula = Value ~ Week, data = Caproate_R_DC)
## 
## $Week
##                    diff        lwr        upr     p adj
## C2-C1       -0.56228479 -2.1060208  0.9814512 0.9003944
## COTR1-C1    -3.74826238 -5.2919984 -2.2045264 0.0000065
## COTR2-C1    -3.59512946 -5.1388654 -2.0513935 0.0000112
## COTR3-C1    -3.79962034 -5.3433563 -2.2558844 0.0000054
## TR1-C1      -1.84045436 -3.3841903 -0.2967184 0.0139577
## TR2-C1      -3.62380916 -5.1675451 -2.0800732 0.0000101
## TR3-C1      -3.73254966 -5.2762856 -2.1888137 0.0000069
## COTR1-C2    -3.18597759 -4.7297136 -1.6422416 0.0000507
## COTR2-C2    -3.03284467 -4.5765807 -1.4891087 0.0000918
## COTR3-C2    -3.23733554 -4.7810715 -1.6935996 0.0000417
## TR1-C2      -1.27816956 -2.8219055  0.2655664 0.1455761
## TR2-C2      -3.06152436 -4.6052603 -1.5177884 0.0000820
## TR3-C2      -3.17026486 -4.7140008 -1.6265289 0.0000539
## COTR2-COTR1  0.15313292 -1.3906031  1.6968689 0.9999582
## COTR3-COTR1 -0.05135795 -1.5950939  1.4923780 1.0000000
## TR1-COTR1    1.90780802  0.3640720  3.4515440 0.0103991
## TR2-COTR1    0.12445323 -1.4192828  1.6681892 0.9999898
## TR3-COTR1    0.01571272 -1.5280233  1.5594487 1.0000000
## COTR3-COTR2 -0.20449087 -1.7482269  1.3392451 0.9997115
## TR1-COTR2    1.75467510  0.2109391  3.2984111 0.0202752
## TR2-COTR2   -0.02867969 -1.5724157  1.5150563 1.0000000
## TR3-COTR2   -0.13742020 -1.6811562  1.4063158 0.9999799
```

```
## TR1-COTR3    1.95916598  0.4154300  3.5029020 0.0083064
## TR2-COTR3    0.17581118 -1.3679248  1.7195472 0.9998941
## TR3-COTR3    0.06707068 -1.4766653  1.6108067 0.9999999
## TR2-TR1     -1.78335480 -3.3270908 -0.2396188 0.0179002
## TR3-TR1     -1.89209530 -3.4358313 -0.3483593 0.0111388
## TR3-TR2     -0.10874050 -1.6524765  1.4349955 0.9999960
```

```r
print("For Roundup in TC")
```

```
## [1] "For Roundup in TC"
```

```r
Caproate_R_TC <- Caproate[Caproate$Arm == 'roundup' & Caproate$Compartment == 'TC',]
res.aov <- aov(Value ~ Week, data = Caproate_R_TC)
summary(res.aov)
```

```
##             Df Sum Sq Mean Sq F value   Pr(>F)    
## Week         7 15.090   2.156   14.18 8.82e-06 ***
## Residuals   16  2.433   0.152                     
## ---
## Signif. codes:  0 '***' 0.001 '**' 0.01 '*' 0.05 '.' 0.1 ' ' 1
```

```r
TukeyHSD(res.aov, which = "Week")
```

```
##   Tukey multiple comparisons of means
##     95% family-wise confidence level
## 
## Fit: aov(formula = Value ~ Week, data = Caproate_R_TC)
## 
## $Week
##                    diff         lwr         upr     p adj
## C2-C1       -0.26940857 -1.37162894  0.83281179 0.9870878
## COTR1-C1    -1.93162563 -3.03384599 -0.82940527 0.0003416
## COTR2-C1    -1.85170894 -2.95392930 -0.74948857 0.0005430
## COTR3-C1    -2.08958049 -3.19180085 -0.98736013 0.0001396
## TR1-C1      -0.90491879 -2.00713915  0.19730157 0.1516375
## TR2-C1      -1.91225631 -3.01447667 -0.81003595 0.0003820
## TR3-C1      -1.97522810 -3.07744847 -0.87300774 0.0002661
## COTR1-C2    -1.66221706 -2.76443742 -0.55999670 0.0016710
## COTR2-C2    -1.58230036 -2.68452072 -0.48008000 0.0027085
## COTR3-C2    -1.82017192 -2.92239228 -0.71795156 0.0006532
## TR1-C2      -0.63551022 -1.73773058  0.46671015 0.5129135
## TR2-C2      -1.64284774 -2.74506810 -0.54062738 0.0018778
## TR3-C2      -1.70581953 -2.80803989 -0.60359917 0.0012865
## COTR2-COTR1  0.07991670 -1.02230367  1.18213706 0.9999951
## COTR3-COTR1 -0.15795486 -1.26017522  0.94426550 0.9995174
## TR1-COTR1    1.02670684 -0.07551352  2.12892720 0.0774717
## TR2-COTR1    0.01936932 -1.08285104  1.12158968 1.0000000
## TR3-COTR1   -0.04360247 -1.14582283  1.05861789 0.9999999
## COTR3-COTR2 -0.23787156 -1.34009192  0.86434880 0.9937417
## TR1-COTR2    0.94679015 -0.15543022  2.04901051 0.1210813
## TR2-COTR2   -0.06054738 -1.16276774  1.04167298 0.9999993
## TR3-COTR2   -0.12351917 -1.22573953  0.97870119 0.9999050
## TR1-COTR3    1.18466170  0.08244134  2.28688206 0.0306160
## TR2-COTR3    0.17732418 -0.92489618  1.27954454 0.9989824
## TR3-COTR3    0.11435239 -0.98786797  1.21657275 0.9999434
## TR2-TR1     -1.00733752 -2.10955788  0.09488284 0.0864827
```

```
## TR3-TR1     -1.07030931 -2.17252968  0.03191105 0.0602588
## TR3-TR2     -0.06297179 -1.16519215  1.03924857 0.9999990
```

B-SCFA

```
### bSCFA ####
bSCFA <- microbial_activity[microbial_activity$Compound == 'bSCFA',]

G <- ggplot(data = bSCFA, aes(x = Time, y = Value, colour = Time, fill = Time)) +
  geom_boxplot(alpha = 0.5, outlier.color = 'white') +
  stat_summary(aes(group = 1), geom = "point", fun = mean, shape = 17, size = 1) +
  stat_summary(aes(group = 1), geom = "line",  size = 0.2, fun = mean) +
  scale_x_discrete(breaks = c(1,2,3,4,5,6,7,8), labels = c("C", "C", "TR", "TR", "TR", "COTR", "COTR",
  scale_fill_manual(values = c("black", "black", "red", "red", "red", "blue", "blue", "blue")) +
  scale_colour_manual(values = c("black", "black", "red", "red", "red", "blue", "blue", "blue")) +
  ylab("bSCFA (mM)") +
  facet_grid(Compartment ~ Arm, scales = 'free') + theme_bw() +
  theme(axis.text.x = element_text(angle = 90, vjust = 0.5, hjust=1), text = element_text(size = 12)) +
  theme(legend.position = "none", axis.title.x=element_blank(), axis.text.x =element_blank()) + labs(ta

print("For glyphosate in AC")
```

```
## [1] "For glyphosate in AC"
```

```
bSCFA_G_AC <- bSCFA[bSCFA$Arm == 'glyphosate' & bSCFA$Compartment == 'AC',]
res.aov <- aov(Value ~ Week, data = bSCFA_G_AC)
summary(res.aov)
```

```
##             Df  Sum Sq  Mean Sq F value Pr(>F)
## Week         7 0.02155 0.003079   0.331  0.928
## Residuals   16 0.14878 0.009298
```

```
TukeyHSD(res.aov, which = "Week")
```

```
##   Tukey multiple comparisons of means
##     95% family-wise confidence level
##
## Fit: aov(formula = Value ~ Week, data = bSCFA_G_AC)
##
## $Week
##                     diff        lwr       upr     p adj
## C2-C1         0.052523449 -0.2200645 0.3251114 0.9968462
## COTR1-C1      0.010356255 -0.2622317 0.2829442 0.9999999
## COTR2-C1      0.027249179 -0.2453388 0.2998371 0.9999559
## COTR3-C1      0.005080897 -0.2675070 0.2776688 1.0000000
## TR1-C1       -0.044361206 -0.3169491 0.2282267 0.9989049
## TR2-C1       -0.032557910 -0.3051458 0.2400300 0.9998546
## TR3-C1       -0.022902150 -0.2954901 0.2496858 0.9999865
## COTR1-C2     -0.042167194 -0.3147551 0.2304207 0.9992085
## COTR2-C2     -0.025274271 -0.2978622 0.2473137 0.9999735
## COTR3-C2     -0.047442553 -0.3200305 0.2251454 0.9983246
## TR1-C2       -0.096884656 -0.3694726 0.1757033 0.9107965
## TR2-C2       -0.085081359 -0.3576693 0.1875066 0.9520852
## TR3-C2       -0.075425599 -0.3480135 0.1971623 0.9743626
## COTR2-COTR1   0.016892924 -0.2556950 0.2894809 0.9999983
## COTR3-COTR1  -0.005275358 -0.2778633 0.2673126 1.0000000
```

```
## TR1-COTR1   -0.054717461 -0.3273054 0.2178705 0.9959505
## TR2-COTR1   -0.042914165 -0.3155021 0.2296738 0.9991141
## TR3-COTR1   -0.033258405 -0.3058463 0.2393295 0.9998325
## COTR3-COTR2 -0.022168282 -0.2947562 0.2504196 0.9999892
## TR1-COTR2   -0.071610385 -0.3441983 0.2009775 0.9806673
## TR2-COTR2   -0.059807089 -0.3323950 0.2127808 0.9930980
## TR3-COTR2   -0.050151328 -0.3227393 0.2224366 0.9976294
## TR1-COTR3   -0.049442103 -0.3220300 0.2231458 0.9978304
## TR2-COTR3   -0.037638806 -0.3102267 0.2349491 0.9996213
## TR3-COTR3   -0.027983046 -0.3005710 0.2446049 0.9999473
## TR2-TR1      0.011803296 -0.2607846 0.2843912 0.9999999
## TR3-TR1      0.021459056 -0.2511289 0.2940470 0.9999913
## TR3-TR2      0.009655760 -0.2629322 0.2822437 1.0000000
```

```r
print("For glyphosate in DC")
```

```
## [1] "For glyphosate in DC"
```

```r
bSCFA_G_DC <- bSCFA[bSCFA$Arm == 'glyphosate' & bSCFA$Compartment == 'DC',]
res.aov <- aov(Value ~ Week, data = bSCFA_G_DC)
summary(res.aov)
```

```
##             Df  Sum Sq  Mean Sq F value Pr(>F)
## Week         7 0.06117 0.008739   0.813   0.59
## Residuals   16 0.17204 0.010753
```

```r
TukeyHSD(res.aov, which = "Week")
```

```
##   Tukey multiple comparisons of means
##     95% family-wise confidence level
##
## Fit: aov(formula = Value ~ Week, data = bSCFA_G_DC)
##
## $Week
##                     diff        lwr       upr     p adj
## C2-C1        0.0017683608 -0.2913596 0.2948963 1.0000000
## COTR1-C1    -0.0388628909 -0.3319908 0.2542651 0.9997099
## COTR2-C1    -0.0064099890 -0.2995379 0.2867180 1.0000000
## COTR3-C1    -0.0765347786 -0.3696627 0.2165932 0.9813112
## TR1-C1       0.0869873758 -0.2061406 0.3801153 0.9629371
## TR2-C1       0.0237158589 -0.2694121 0.3168438 0.9999895
## TR3-C1      -0.0758688218 -0.3689968 0.2172591 0.9821934
## COTR1-C2    -0.0406312517 -0.3337592 0.2524967 0.9996116
## COTR2-C2    -0.0081783498 -0.3013063 0.2849496 1.0000000
## COTR3-C2    -0.0783031394 -0.3714311 0.2148248 0.9788130
## TR1-C2       0.0852190149 -0.2079089 0.3783470 0.9666882
## TR2-C2       0.0219474981 -0.2711804 0.3150754 0.9999938
## TR3-C2      -0.0776371826 -0.3707651 0.2154908 0.9797808
## COTR2-COTR1  0.0324529019 -0.2606750 0.3255808 0.9999124
## COTR3-COTR1 -0.0376718877 -0.3307998 0.2554561 0.9997637
## TR1-COTR1    0.1258502667 -0.1672777 0.4189782 0.8037878
## TR2-COTR1    0.0625787498 -0.2305492 0.3557067 0.9941326
## TR3-COTR1   -0.0370059309 -0.3301339 0.2561220 0.9997899
## COTR3-COTR2 -0.0701247896 -0.3632527 0.2230032 0.9885775
## TR1-COTR2    0.0933973647 -0.1997306 0.3865253 0.9469153
## TR2-COTR2    0.0301258479 -0.2630021 0.3232538 0.9999469
```

```
## TR3-COTR2   -0.0694588328 -0.3625868 0.2236691 0.9891864
## TR1-COTR3    0.1635221544 -0.1296058 0.4566501 0.5508103
## TR2-COTR3    0.1002506375 -0.1928773 0.3933786 0.9253368
## TR3-COTR3    0.0006659568 -0.2924620 0.2937939 1.0000000
## TR2-TR1     -0.0632715168 -0.3563995 0.2298564 0.9937351
## TR3-TR1     -0.1628561976 -0.4559841 0.1302717 0.5554499
## TR3-TR2     -0.0995846807 -0.3927126 0.1935433 0.9276416
```

```
print("For glyphosate in TC")
```

```
## [1] "For glyphosate in TC"
```

```
bSCFA_G_TC <- bSCFA[bSCFA$Arm == 'glyphosate' & bSCFA$Compartment == 'TC',]
res.aov <- aov(Value ~ Week, data = bSCFA_G_TC)
summary(res.aov)
```

```
##             Df Sum Sq Mean Sq F value Pr(>F)
## Week         7 0.3169 0.04527   5.455 0.0024 **
## Residuals   16 0.1328 0.00830
## ---
## Signif. codes:  0 '***' 0.001 '**' 0.01 '*' 0.05 '.' 0.1 ' ' 1
```

```
TukeyHSD(res.aov, which = "Week")
```

```
##   Tukey multiple comparisons of means
##     95% family-wise confidence level
##
## Fit: aov(formula = Value ~ Week, data = bSCFA_G_TC)
##
## $Week
##                     diff        lwr          upr     p adj
## C2-C1        0.007274058 -0.2502327  0.264780838 1.0000000
## COTR1-C1    -0.088821128 -0.3463279  0.168685652 0.9223035
## COTR2-C1    -0.021109131 -0.2786159  0.236397649 0.9999886
## COTR3-C1    -0.135240781 -0.3927476  0.122265999 0.6178942
## TR1-C1      -0.303018248 -0.5605250 -0.045511468 0.0154897
## TR2-C1      -0.287640519 -0.5451473 -0.030133739 0.0231183
## TR3-C1      -0.171384660 -0.4288914  0.086122120 0.3480900
## COTR1-C2    -0.096095187 -0.3536020  0.161411593 0.8891537
## COTR2-C2    -0.028383189 -0.2858900  0.229123591 0.9999149
## COTR3-C2    -0.142514840 -0.4000216  0.114991940 0.5598252
## TR1-C2      -0.310292306 -0.5677991 -0.052785526 0.0128058
## TR2-C2      -0.294914577 -0.5524214 -0.037407797 0.0191362
## TR3-C2      -0.178658718 -0.4361655  0.078848062 0.3031818
## COTR2-COTR1  0.067711997 -0.1897948  0.325218777 0.9805672
## COTR3-COTR1 -0.046419653 -0.3039264  0.211087127 0.9979122
## TR1-COTR1   -0.214197120 -0.4717039  0.043309660 0.1423285
## TR2-COTR1   -0.198819391 -0.4563262  0.058687389 0.2003499
## TR3-COTR1   -0.082563532 -0.3400703  0.174943248 0.9452501
## COTR3-COTR2 -0.114131651 -0.3716384  0.143375129 0.7792593
## TR1-COTR2   -0.281909117 -0.5394159 -0.024402337 0.0268154
## TR2-COTR2   -0.266531388 -0.5240382 -0.009024608 0.0397862
## TR3-COTR2   -0.150275529 -0.4077823  0.107231251 0.4989236
## TR1-COTR3   -0.167777467 -0.4252842  0.089729313 0.3718333
## TR2-COTR3   -0.152399737 -0.4099065  0.105107043 0.4826191
## TR3-COTR3   -0.036143878 -0.2936507  0.221362902 0.9995785
```

```
## TR2-TR1      0.015377729 -0.2421291  0.272884509 0.9999987
## TR3-TR1      0.131633588 -0.1258732  0.389140368 0.6466437
## TR3-TR2      0.116255859 -0.1412509  0.373762639 0.7641608
```

```r
print("For Roundup in AC")
```

```
## [1] "For Roundup in AC"
```

```r
bSCFA_R_AC <- bSCFA[bSCFA$Arm == 'roundup' & bSCFA$Compartment == 'AC',]
res.aov <- aov(Value ~ Week, data = bSCFA_R_AC)
summary(res.aov)
```

```
##             Df Sum Sq Mean Sq F value Pr(>F)
## Week         7 0.3169 0.04527   5.455 0.0024 **
## Residuals   16 0.1328 0.00830
## ---
## Signif. codes:  0 '***' 0.001 '**' 0.01 '*' 0.05 '.' 0.1 ' ' 1
```

```r
TukeyHSD(res.aov, which = "Week")
```

```
##   Tukey multiple comparisons of means
##     95% family-wise confidence level
##
## Fit: aov(formula = Value ~ Week, data = bSCFA_R_AC)
##
## $Week
##                     diff        lwr          upr     p adj
## C2-C1        0.007274058 -0.2502327  0.264780838 1.0000000
## COTR1-C1    -0.088821128 -0.3463279  0.168685652 0.9223035
## COTR2-C1    -0.021109131 -0.2786159  0.236397649 0.9999886
## COTR3-C1    -0.135240781 -0.3927476  0.122265999 0.6178942
## TR1-C1      -0.303018248 -0.5605250 -0.045511468 0.0154897
## TR2-C1      -0.287640519 -0.5451473 -0.030133739 0.0231183
## TR3-C1      -0.171384660 -0.4288914  0.086122120 0.3480900
## COTR1-C2    -0.096095187 -0.3536020  0.161411593 0.8891537
## COTR2-C2    -0.028383189 -0.2858900  0.229123591 0.9999149
## COTR3-C2    -0.142514840 -0.4000216  0.114991940 0.5598252
## TR1-C2      -0.310292306 -0.5677991 -0.052785526 0.0128058
## TR2-C2      -0.294914577 -0.5524214 -0.037407797 0.0191362
## TR3-C2      -0.178658718 -0.4361655  0.078848062 0.3031818
## COTR2-COTR1  0.067711997 -0.1897948  0.325218777 0.9805672
## COTR3-COTR1 -0.046419653 -0.3039264  0.211087127 0.9979122
## TR1-COTR1   -0.214197120 -0.4717039  0.043309660 0.1423285
## TR2-COTR1   -0.198819391 -0.4563262  0.058687389 0.2003499
## TR3-COTR1   -0.082563532 -0.3400703  0.174943248 0.9452501
## COTR3-COTR2 -0.114131651 -0.3716384  0.143375129 0.7792593
## TR1-COTR2   -0.281909117 -0.5394159 -0.024402337 0.0268154
## TR2-COTR2   -0.266531388 -0.5240382 -0.009024608 0.0397862
## TR3-COTR2   -0.150275529 -0.4077823  0.107231251 0.4989236
## TR1-COTR3   -0.167777467 -0.4252842  0.089729313 0.3718333
## TR2-COTR3   -0.152399737 -0.4099065  0.105107043 0.4826191
## TR3-COTR3   -0.036143878 -0.2936507  0.221362902 0.9995785
## TR2-TR1      0.015377729 -0.2421291  0.272884509 0.9999987
## TR3-TR1      0.131633588 -0.1258732  0.389140368 0.6466437
## TR3-TR2      0.116255859 -0.1412509  0.373762639 0.7641608
```

```r
print("For Roundup in DC")
```

```
## [1] "For Roundup in DC"
```

```r
bSCFA_R_DC <- bSCFA[bSCFA$Arm == 'roundup' & bSCFA$Compartment == 'DC',]
res.aov <- aov(Value ~ Week, data = bSCFA_R_DC)
summary(res.aov)
```

```
##             Df  Sum Sq Mean Sq F value Pr(>F)
## Week         7 0.07133 0.01019   0.637  0.719
## Residuals   16 0.25581 0.01599
```

```r
TukeyHSD(res.aov, which = "Week")
```

```
##   Tukey multiple comparisons of means
##     95% family-wise confidence level
##
## Fit: aov(formula = Value ~ Week, data = bSCFA_R_DC)
##
## $Week
##                     diff        lwr       upr     p adj
## C2-C1        0.027211451 -0.3302282 0.3846511 0.9999931
## COTR1-C1     0.022333148 -0.3351065 0.3797728 0.9999982
## COTR2-C1    -0.060571215 -0.4180108 0.2968684 0.9985842
## COTR3-C1     0.014804062 -0.3426356 0.3722437 0.9999999
## TR1-C1      -0.107358784 -0.4647984 0.2500808 0.9605648
## TR2-C1      -0.099453208 -0.4568928 0.2579864 0.9735906
## TR3-C1      -0.088739295 -0.4461789 0.2687003 0.9858959
## COTR1-C2    -0.004878303 -0.3623179 0.3525613 1.0000000
## COTR2-C2    -0.087782666 -0.4452223 0.2696570 0.9867349
## COTR3-C2    -0.012407388 -0.3698470 0.3450322 1.0000000
## TR1-C2      -0.134570235 -0.4920099 0.2228694 0.8848199
## TR2-C2      -0.126664659 -0.4841043 0.2307750 0.9120008
## TR3-C2      -0.115950746 -0.4733904 0.2414889 0.9420277
## COTR2-COTR1 -0.082904363 -0.4403440 0.2745353 0.9904439
## COTR3-COTR1 -0.007529085 -0.3649687 0.3499105 1.0000000
## TR1-COTR1   -0.129691931 -0.4871315 0.2277477 0.9020929
## TR2-COTR1   -0.121786356 -0.4792260 0.2356533 0.9266453
## TR3-COTR1   -0.111072443 -0.4685121 0.2463672 0.9531376
## COTR3-COTR2  0.075375278 -0.2820643 0.4328149 0.9945485
## TR1-COTR2   -0.046787569 -0.4042272 0.3106520 0.9997333
## TR2-COTR2   -0.038881993 -0.3963216 0.3185576 0.9999221
## TR3-COTR2   -0.028168080 -0.3856077 0.3292715 0.9999913
## TR1-COTR3   -0.122162846 -0.4796025 0.2352768 0.9255732
## TR2-COTR3   -0.114257271 -0.4716969 0.2431823 0.9460622
## TR3-COTR3   -0.103543357 -0.4609830 0.2538963 0.9673082
## TR2-TR1      0.007905576 -0.3495340 0.3653452 1.0000000
## TR3-TR1      0.018619489 -0.3388201 0.3760591 0.9999995
## TR3-TR2      0.010713913 -0.3467257 0.3681535 1.0000000
```

```r
print("For Roundup in TC")
```

```
## [1] "For Roundup in TC"
```

```r
bSCFA_R_TC <- bSCFA[bSCFA$Arm == 'roundup' & bSCFA$Compartment == 'TC',]
res.aov <- aov(Value ~ Week, data = bSCFA_R_TC)
```

```r
summary(res.aov)
```

```
##             Df Sum Sq Mean Sq F value Pr(>F)
## Week         7 0.1078 0.01541   0.823  0.583
## Residuals   16 0.2996 0.01873
```

```r
TukeyHSD(res.aov, which = "Week")
```

```
##   Tukey multiple comparisons of means
##     95% family-wise confidence level
##
## Fit: aov(formula = Value ~ Week, data = bSCFA_R_TC)
##
## $Week
##                    diff        lwr       upr     p adj
## C2-C1       -0.05144013 -0.4382843 0.3354041 0.9997042
## COTR1-C1    -0.02618682 -0.4130310 0.3606574 0.9999969
## COTR2-C1    -0.07982865 -0.4666728 0.3070155 0.9952134
## COTR3-C1    -0.14918562 -0.5360298 0.2376586 0.8723349
## TR1-C1      -0.13552418 -0.5223684 0.2513200 0.9164992
## TR2-C1      -0.21906785 -0.6059120 0.1677763 0.5336387
## TR3-C1      -0.08441421 -0.4712584 0.3024300 0.9933170
## COTR1-C2     0.02525331 -0.3615909 0.4120975 0.9999976
## COTR2-C2    -0.02838852 -0.4152327 0.3584557 0.9999946
## COTR3-C2    -0.09774549 -0.4845897 0.2890987 0.9844274
## TR1-C2      -0.08408405 -0.4709282 0.3027601 0.9934704
## TR2-C2      -0.16762772 -0.5544719 0.2192165 0.7968659
## TR3-C2      -0.03297408 -0.4198183 0.3538701 0.9999851
## COTR2-COTR1 -0.05364183 -0.4404860 0.3332023 0.9996106
## COTR3-COTR1 -0.12299880 -0.5098430 0.2638454 0.9474637
## TR1-COTR1   -0.10933736 -0.4961815 0.2775068 0.9712862
## TR2-COTR1   -0.19288103 -0.5797252 0.1939632 0.6722556
## TR3-COTR1   -0.05822739 -0.4450716 0.3286168 0.9993364
## COTR3-COTR2 -0.06935697 -0.4562012 0.3174872 0.9979819
## TR1-COTR2   -0.05569553 -0.4425397 0.3311487 0.9995025
## TR2-COTR2   -0.13923920 -0.5260834 0.2476050 0.9055557
## TR3-COTR2   -0.00458556 -0.3914297 0.3822586 1.0000000
## TR1-COTR3    0.01366144 -0.3731827 0.4005056 1.0000000
## TR2-COTR3   -0.06988223 -0.4567264 0.3169620 0.9978845
## TR3-COTR3    0.06477141 -0.3220728 0.4516156 0.9986879
## TR2-TR1     -0.08354367 -0.4703879 0.3033005 0.9937156
## TR3-TR1      0.05110997 -0.3357342 0.4379542 0.9997164
## TR3-TR2      0.13465364 -0.2521905 0.5214978 0.9189468
```

Ammonium

```r
### Ammonium ####
Ammonium <- microbial_activity[microbial_activity$Compound == 'Ammonium',]

H <- ggplot(data = Ammonium, aes(x = Time, y = Value, colour = Time, fill = Time)) +
  geom_boxplot(alpha = 0.5, outlier.color = 'white') +
  stat_summary(aes(group = 1), geom = "point", fun = mean, shape = 17, size = 1) +
  stat_summary(aes(group = 1), geom = "line",  size = 0.2, fun = mean) +
  scale_x_discrete(breaks = c(1,2,3,4,5,6,7,8), labels = c("C", "C", "TR", "TR", "TR", "COTR", "COTR",
```

```r
  scale_fill_manual(values = c("black", "black", "red", "red", "red", "blue", "blue", "blue")) +
  scale_colour_manual(values = c("black", "black", "red", "red", "red", "blue", "blue", "blue")) +
  ylab("Ammonium (mg/L)") +
  facet_grid(Compartment ~ Arm, scales = 'free') + theme_bw() +
  theme(axis.text.x = element_text(angle = 90, vjust = 0.5, hjust=1), text = element_text(size = 12)) +
  theme(legend.position = "none", axis.title.x=element_blank(), axis.text.x =element_blank()) + labs(ta

print("For glyphosate in AC")
```

```
## [1] "For glyphosate in AC"
```

```r
Ammonium_G_AC <- Ammonium[Ammonium$Arm == 'glyphosate' & Ammonium$Compartment == 'AC',]
res.aov <- aov(Value ~ Week, data = Ammonium_G_AC)
summary(res.aov)
```

```
##             Df Sum Sq Mean Sq F value Pr(>F)
## Week         7  556.5   79.51   0.416  0.878
## Residuals   16 3056.9  191.05
```

```r
TukeyHSD(res.aov, which = "Week")
```

```
##   Tukey multiple comparisons of means
##     95% family-wise confidence level
##
## Fit: aov(formula = Value ~ Week, data = Ammonium_G_AC)
##
## $Week
##                    diff       lwr      upr     p adj
## C2-C1       -4.04666667 -43.11981 35.02648 0.9999440
## COTR1-C1   -11.17000000 -50.24315 27.90315 0.9695039
## COTR2-C1   -10.27000000 -49.34315 28.80315 0.9806126
## COTR3-C1    -3.45333333 -42.52648 35.61981 0.9999809
## TR1-C1     -11.79333333 -50.86648 27.27981 0.9595652
## TR2-C1     -11.23333333 -50.30648 27.83981 0.9685821
## TR3-C1      -0.05666667 -39.12981 39.01648 1.0000000
## COTR1-C2    -7.12333333 -46.19648 31.94981 0.9977604
## COTR2-C2    -6.22333333 -45.29648 32.84981 0.9990456
## COTR3-C2     0.59333333 -38.47981 39.66648 1.0000000
## TR1-C2      -7.74666667 -46.81981 31.32648 0.9962447
## TR2-C2      -7.18666667 -46.25981 31.88648 0.9976337
## TR3-C2       3.99000000 -35.08315 43.06315 0.9999491
## COTR2-COTR1  0.90000000 -38.17315 39.97315 1.0000000
## COTR3-COTR1  7.71666667 -31.35648 46.78981 0.9963325
## TR1-COTR1   -0.62333333 -39.69648 38.44981 1.0000000
## TR2-COTR1   -0.06333333 -39.13648 39.00981 1.0000000
## TR3-COTR1   11.11333333 -27.95981 50.18648 0.9703124
## COTR3-COTR2  6.81666667 -32.25648 45.88981 0.9982993
## TR1-COTR2   -1.52333333 -40.59648 37.54981 0.9999999
## TR2-COTR2   -0.96333333 -40.03648 38.10981 1.0000000
## TR3-COTR2   10.21333333 -28.85981 49.28648 0.9811950
## TR1-COTR3   -8.34000000 -47.41315 30.73315 0.9941392
## TR2-COTR3   -7.78000000 -46.85315 31.29315 0.9961452
## TR3-COTR3    3.39666667 -35.67648 42.46981 0.9999829
## TR2-TR1      0.56000000 -38.51315 39.63315 1.0000000
## TR3-TR1     11.73666667 -27.33648 50.80981 0.9605501
```

```
## TR3-TR2      11.17666667 -27.89648 50.24981 0.9694078
```

```r
print("For glyphosate in DC")
```

```
## [1] "For glyphosate in DC"
```

```r
Ammonium_G_DC <- Ammonium[Ammonium$Arm == 'glyphosate' & Ammonium$Compartment == 'DC',]
res.aov <- aov(Value ~ Week, data = Ammonium_G_DC)
summary(res.aov)
```

```
##             Df Sum Sq Mean Sq F value Pr(>F)
## Week         7   1103   157.6   0.635  0.721
## Residuals   16   3973   248.3
```

```r
TukeyHSD(res.aov, which = "Week")
```

```
##   Tukey multiple comparisons of means
##     95% family-wise confidence level
##
## Fit: aov(formula = Value ~ Week, data = Ammonium_G_DC)
##
## $Week
##                   diff       lwr      upr     p adj
## C2-C1        -4.4833333 -49.03008 40.06341 0.9999539
## COTR1-C1    -12.2933333 -56.84008 32.25341 0.9747273
## COTR2-C1    -15.2233333 -59.77008 29.32341 0.9256075
## COTR3-C1     -9.1500000 -53.69675 35.39675 0.9953457
## TR1-C1      -12.1700000 -56.71675 32.37675 0.9760639
## TR2-C1        5.3866667 -39.16008 49.93341 0.9998421
## TR3-C1       -0.7800000 -45.32675 43.76675 1.0000000
## COTR1-C2     -7.8100000 -52.35675 36.73675 0.9982458
## COTR2-C2    -10.7400000 -55.28675 33.80675 0.9880582
## COTR3-C2     -4.6666667 -49.21341 39.88008 0.9999395
## TR1-C2       -7.6866667 -52.23341 36.86008 0.9984129
## TR2-C2        9.8700000 -34.67675 54.41675 0.9926871
## TR3-C2        3.7033333 -40.84341 48.25008 0.9999874
## COTR2-COTR1  -2.9300000 -47.47675 41.61675 0.9999975
## COTR3-COTR1   3.1433333 -41.40341 47.69008 0.9999959
## TR1-COTR1     0.1233333 -44.42341 44.67008 1.0000000
## TR2-COTR1    17.6800000 -26.86675 62.22675 0.8560826
## TR3-COTR1    11.5133333 -33.03341 56.06008 0.9823340
## COTR3-COTR2   6.0733333 -38.47341 50.62008 0.9996515
## TR1-COTR2     3.0533333 -41.49341 47.60008 0.9999966
## TR2-COTR2    20.6100000 -23.93675 65.15675 0.7431224
## TR3-COTR2    14.4433333 -30.10341 58.99008 0.9421704
## TR1-COTR3    -3.0200000 -47.56675 41.52675 0.9999969
## TR2-COTR3    14.5366667 -30.01008 59.08341 0.9403282
## TR3-COTR3     8.3700000 -36.17675 52.91675 0.9972994
## TR2-TR1      17.5566667 -26.99008 62.10341 0.8601804
## TR3-TR1      11.3900000 -33.15675 55.93675 0.9833610
## TR3-TR2      -6.1666667 -50.71341 38.38008 0.9996149
```

```r
print("For glyphosate in TC")
```

```
## [1] "For glyphosate in TC"
```

```r
Ammonium_G_TC <- Ammonium[Ammonium$Arm == 'glyphosate' & Ammonium$Compartment == 'TC',]
res.aov <- aov(Value ~ Week, data = Ammonium_G_TC)
summary(res.aov)
```

```
##             Df Sum Sq Mean Sq F value Pr(>F)
## Week         7   1726   246.6   1.052  0.436
## Residuals   16   3751   234.4
```

```r
TukeyHSD(res.aov, which = "Week")
```

```
##   Tukey multiple comparisons of means
##     95% family-wise confidence level
##
## Fit: aov(formula = Value ~ Week, data = Ammonium_G_TC)
##
## $Week
##                    diff       lwr      upr     p adj
## C2-C1        23.3166667 -19.96664 66.59998 0.5900924
## COTR1-C1      5.1666667 -38.11664 48.44998 0.9998551
## COTR2-C1     -1.7766667 -45.05998 41.50664 0.9999999
## COTR3-C1      9.1500000 -34.13331 52.43331 0.9944672
## TR1-C1       21.4466667 -21.83664 64.72998 0.6785313
## TR2-C1        8.2766667 -35.00664 51.55998 0.9969906
## TR3-C1        7.2233333 -36.05998 50.50664 0.9987150
## COTR1-C2    -18.1500000 -61.43331 25.13331 0.8207096
## COTR2-C2    -25.0933333 -68.37664 18.18998 0.5065617
## COTR3-C2    -14.1666667 -57.44998 29.11664 0.9394562
## TR1-C2       -1.8700000 -45.15331 41.41331 0.9999999
## TR2-C2      -15.0400000 -58.32331 28.24331 0.9195965
## TR3-C2      -16.0933333 -59.37664 27.18998 0.8909101
## COTR2-COTR1  -6.9433333 -50.22664 36.33998 0.9990010
## COTR3-COTR1   3.9833333 -39.29998 47.26664 0.9999748
## TR1-COTR1    16.2800000 -27.00331 59.56331 0.8852924
## TR2-COTR1     3.1100000 -40.17331 46.39331 0.9999954
## TR3-COTR1     2.0566667 -41.22664 45.33998 0.9999997
## COTR3-COTR2  10.9266667 -32.35664 54.20998 0.9845063
## TR1-COTR2    23.2233333 -20.05998 66.50664 0.5945305
## TR2-COTR2    10.0533333 -33.22998 53.33664 0.9903651
## TR3-COTR2     9.0000000 -34.28331 52.28331 0.9949894
## TR1-COTR3    12.2966667 -30.98664 55.57998 0.9704923
## TR2-COTR3    -0.8733333 -44.15664 42.40998 1.0000000
## TR3-COTR3    -1.9266667 -45.20998 41.35664 0.9999998
## TR2-TR1     -13.1700000 -56.45331 30.11331 0.9578652
## TR3-TR1     -14.2233333 -57.50664 29.05998 0.9382749
## TR3-TR2      -1.0533333 -44.33664 42.22998 1.0000000
```

```r
print("For Roundup in AC")
```

```
## [1] "For Roundup in AC"
```

```r
Ammonium_R_AC <- Ammonium[Ammonium$Arm == 'roundup' & Ammonium$Compartment == 'AC',]
res.aov <- aov(Value ~ Week, data = Ammonium_R_AC)
summary(res.aov)
```

```
##             Df  Sum Sq Mean Sq F value   Pr(>F)
## Week         7 1487241  212463   121.9 1.12e-12 ***
```

```
## Residuals   16   27876    1742
## ---
## Signif. codes:  0 '***' 0.001 '**' 0.01 '*' 0.05 '.' 0.1 ' ' 1
```

```r
TukeyHSD(res.aov, which = "Week")
```

```
##   Tukey multiple comparisons of means
##     95% family-wise confidence level
##
## Fit: aov(formula = Value ~ Week, data = Ammonium_R_AC)
##
## $Week
##                     diff         lwr        upr     p adj
## C2-C1         -14.4700000 -132.461874 103.521874 0.9998267
## COTR1-C1      589.2500000  471.258126 707.241874 0.0000000
## COTR2-C1      577.0800000  459.088126 695.071874 0.0000000
## COTR3-C1      577.8266667  459.834792 695.818541 0.0000000
## TR1-C1        473.8666667  355.874792 591.858541 0.0000000
## TR2-C1        570.2333333  452.241459 688.225208 0.0000000
## TR3-C1        583.9600000  465.968126 701.951874 0.0000000
## COTR1-C2      603.7200000  485.728126 721.711874 0.0000000
## COTR2-C2      591.5500000  473.558126 709.541874 0.0000000
## COTR3-C2      592.2966667  474.304792 710.288541 0.0000000
## TR1-C2        488.3366667  370.344792 606.328541 0.0000000
## TR2-C2        584.7033333  466.711459 702.695208 0.0000000
## TR3-C2        598.4300000  480.438126 716.421874 0.0000000
## COTR2-COTR1   -12.1700000 -130.161874 105.821874 0.9999456
## COTR3-COTR1   -11.4233333 -129.415208 106.568541 0.9999645
## TR1-COTR1    -115.3833333 -233.375208   2.608541 0.0576693
## TR2-COTR1     -19.0166667 -137.008541  98.975208 0.9989706
## TR3-COTR1      -5.2900000 -123.281874 112.701874 0.9999998
## COTR3-COTR2     0.7466667 -117.245208 118.738541 1.0000000
## TR1-COTR2    -103.2133333 -221.205208  14.778541 0.1100718
## TR2-COTR2      -6.8466667 -124.838541 111.145208 0.9999989
## TR3-COTR2       6.8800000 -111.111874 124.871874 0.9999989
## TR1-COTR3    -103.9600000 -221.951874  14.031874 0.1059081
## TR2-COTR3      -7.5933333 -125.585208 110.398541 0.9999978
## TR3-COTR3       6.1333333 -111.858541 124.125208 0.9999995
## TR2-TR1        96.3666667  -21.625208 214.358541 0.1554557
## TR3-TR1       110.0933333   -7.898541 228.085208 0.0767105
## TR3-TR2        13.7266667 -104.265208 131.718541 0.9998780
```

```r
print("For Roundup in DC")
```

```
## [1] "For Roundup in DC"
```

```r
Ammonium_R_DC <- Ammonium[Ammonium$Arm == 'roundup' & Ammonium$Compartment == 'DC',]
res.aov <- aov(Value ~ Week, data = Ammonium_R_DC)
summary(res.aov)
```

```
##             Df  Sum Sq Mean Sq F value   Pr(>F)
## Week         7 1133500  161929   29.95 4.82e-08 ***
## Residuals   16   86500    5406
## ---
## Signif. codes:  0 '***' 0.001 '**' 0.01 '*' 0.05 '.' 0.1 ' ' 1
```

```r
TukeyHSD(res.aov, which = "Week")
```

```
##   Tukey multiple comparisons of means
##     95% family-wise confidence level
##
## Fit: aov(formula = Value ~ Week, data = Ammonium_R_DC)
##
## $Week
##                    diff        lwr       upr     p adj
## C2-C1         -4.950000 -212.79911 202.89911 1.0000000
## COTR1-C1     507.666667  299.81756 715.51578 0.0000060
## COTR2-C1     495.186667  287.33756 703.03578 0.0000083
## COTR3-C1     508.726667  300.87756 716.57578 0.0000059
## TR1-C1       269.210000   61.36089 477.05911 0.0069633
## TR2-C1       499.263333  291.41422 707.11244 0.0000075
## TR3-C1       521.483333  313.63422 729.33244 0.0000042
## COTR1-C2     512.616667  304.76756 720.46578 0.0000053
## COTR2-C2     500.136667  292.28756 707.98578 0.0000073
## COTR3-C2     513.676667  305.82756 721.52578 0.0000052
## TR1-C2       274.160000   66.31089 482.00911 0.0059291
## TR2-C2       504.213333  296.36422 712.06244 0.0000066
## TR3-C2       526.433333  318.58422 734.28244 0.0000037
## COTR2-COTR1  -12.480000 -220.32911 195.36911 0.9999987
## COTR3-COTR1    1.060000 -206.78911 208.90911 1.0000000
## TR1-COTR1   -238.456667 -446.30578 -30.60756 0.0188826
## TR2-COTR1     -8.403333 -216.25244 199.44578 0.9999999
## TR3-COTR1     13.816667 -194.03244 221.66578 0.9999973
## COTR3-COTR2   13.540000 -194.30911 221.38911 0.9999976
## TR1-COTR2   -225.976667 -433.82578 -18.12756 0.0281951
## TR2-COTR2      4.076667 -203.77244 211.92578 1.0000000
## TR3-COTR2     26.296667 -181.55244 234.14578 0.9997869
## TR1-COTR3   -239.516667 -447.36578 -31.66756 0.0182474
## TR2-COTR3     -9.463333 -217.31244 198.38578 0.9999998
## TR3-COTR3     12.756667 -195.09244 220.60578 0.9999984
## TR2-TR1      230.053333   22.20422 437.90244 0.0247453
## TR3-TR1      252.273333   44.42422 460.12244 0.0120723
## TR3-TR2       22.220000 -185.62911 230.06911 0.9999307
```

```r
print("For Roundup in TC")
```

```
## [1] "For Roundup in TC"
```

```r
Ammonium_R_TC <- Ammonium[Ammonium$Arm == 'roundup' & Ammonium$Compartment == 'TC',]
res.aov <- aov(Value ~ Week, data = Ammonium_R_TC)
summary(res.aov)
```

```
##             Df  Sum Sq Mean Sq F value   Pr(>F)
## Week         7 1119887  159984   36.31 1.17e-08 ***
## Residuals   16   70491    4406
## ---
## Signif. codes:  0 '***' 0.001 '**' 0.01 '*' 0.05 '.' 0.1 ' ' 1
```

```r
TukeyHSD(res.aov, which = "Week")
```

```
##   Tukey multiple comparisons of means
##     95% family-wise confidence level
```

```
##
## Fit: aov(formula = Value ~ Week, data = Ammonium_R_TC)
##
## $Week
##                   diff         lwr       upr     p adj
## C2-C1         -5.600000 -193.232431 182.03243 1.0000000
## COTR1-C1     501.413333  313.780902 689.04576 0.0000018
## COTR2-C1     505.930000  318.297569 693.56243 0.0000016
## COTR3-C1     508.820000  321.187569 696.45243 0.0000015
## TR1-C1       334.576667  146.944235 522.20910 0.0002814
## TR2-C1       510.563333  322.930902 698.19576 0.0000014
## TR3-C1       512.620000  324.987569 700.25243 0.0000014
## COTR1-C2     507.013333  319.380902 694.64576 0.0000016
## COTR2-C2     511.530000  323.897569 699.16243 0.0000014
## COTR3-C2     514.420000  326.787569 702.05243 0.0000013
## TR1-C2       340.176667  152.544235 527.80910 0.0002333
## TR2-C2       516.163333  328.530902 703.79576 0.0000012
## TR3-C2       518.220000  330.587569 705.85243 0.0000012
## COTR2-COTR1    4.516667 -183.115765 192.14910 1.0000000
## COTR3-COTR1    7.406667 -180.225765 195.03910 0.9999999
## TR1-COTR1   -166.836667 -354.469098  20.79576 0.1007900
## TR2-COTR1      9.150000 -178.482431 196.78243 0.9999997
## TR3-COTR1     11.206667 -176.425765 198.83910 0.9999987
## COTR3-COTR2    2.890000 -184.742431 190.52243 1.0000000
## TR1-COTR2   -171.353333 -358.985765  16.27910 0.0868484
## TR2-COTR2      4.633333 -182.999098 192.26576 1.0000000
## TR3-COTR2      6.690000 -180.942431 194.32243 1.0000000
## TR1-COTR3   -174.243333 -361.875765  13.38910 0.0788711
## TR2-COTR3      1.743333 -185.889098 189.37576 1.0000000
## TR3-COTR3      3.800000 -183.832431 191.43243 1.0000000
## TR2-TR1      175.986667  -11.645765 363.61910 0.0743892
## TR3-TR1      178.043333   -9.589098 365.67576 0.0694038
## TR3-TR2        2.056667 -185.575765 189.68910 1.0000000
```

Lactate

```
### Lactate ####
Lactate <- microbial_activity[microbial_activity$Compound == 'Lactate',]

I <- ggplot(data = Lactate, aes(x = Time, y = Value, colour = Time, fill = Time)) +
  geom_boxplot(alpha = 0.5, outlier.color = 'white') +
  stat_summary(aes(group = 1), geom = "point", fun = mean, shape = 17, size = 1) +
  stat_summary(aes(group = 1), geom = "line",  size = 0.2, fun = mean) +
  scale_x_discrete(breaks = c(1,2,3,4,5,6,7,8), labels = c("C", "C", "TR", "TR", "TR", "COTR", "COTR",
  scale_fill_manual(values = c("black", "black", "red", "red", "red", "blue", "blue", "blue")) +
  scale_colour_manual(values = c("black", "black", "red", "red", "red", "blue", "blue", "blue")) +
  ylab("Lactate (mM)") +
  facet_grid(Compartment ~ Arm, scales = 'free') + theme_bw() +
  theme(axis.text.x = element_text(angle = 90, vjust = 0.5, hjust=1), text = element_text(size = 12)) +
  theme(legend.position = "none", axis.title.x=element_blank(), axis.text.x =element_blank()) + labs(ta

print("For glyphosate in AC")
```

```
## [1] "For glyphosate in AC"
```

```r
Lactate_G_AC <- Lactate[Lactate$Arm == 'glyphosate' & Lactate$Compartment == 'AC',]
res.aov <- aov(Value ~ Week, data = Lactate_G_AC)
summary(res.aov)
```

```
##             Df  Sum Sq   Mean Sq F value  Pr(>F)
## Week         7 0.013355 0.0019079   4.073 0.00951 **
## Residuals   16 0.007495 0.0004684
## ---
## Signif. codes:  0 '***' 0.001 '**' 0.01 '*' 0.05 '.' 0.1 ' ' 1
```

```r
TukeyHSD(res.aov, which = "Week")
```

```
##   Tukey multiple comparisons of means
##     95% family-wise confidence level
##
## Fit: aov(formula = Value ~ Week, data = Lactate_G_AC)
##
## $Week
##                     diff          lwr         upr     p adj
## C2-C1        0.015527611 -0.045655029 0.076710252 0.9840381
## COTR1-C1    -0.016691918 -0.077874558 0.044490723 0.9762410
## COTR2-C1    -0.023783601 -0.084966241 0.037399039 0.8679776
## COTR3-C1    -0.033394420 -0.094577060 0.027788221 0.5754606
## TR1-C1       0.046445234 -0.014737406 0.107627874 0.2152024
## TR2-C1      -0.009515557 -0.070698198 0.051667083 0.9991807
## TR3-C1      -0.011939431 -0.073122071 0.049243209 0.9965911
## COTR1-C2    -0.032219529 -0.093402169 0.028963111 0.6149740
## COTR2-C2    -0.039311212 -0.100493852 0.021871428 0.3876197
## COTR3-C2    -0.048922031 -0.110104671 0.012260609 0.1715703
## TR1-C2       0.030917623 -0.030265017 0.092100263 0.6585873
## TR2-C2      -0.025043169 -0.086225809 0.036139472 0.8369501
## TR3-C2      -0.027467042 -0.088649683 0.033715598 0.7688338
## COTR2-COTR1 -0.007091683 -0.068274324 0.054090957 0.9998809
## COTR3-COTR1 -0.016702502 -0.077885142 0.044480138 0.9761594
## TR1-COTR1    0.063137152  0.001954511 0.124319792 0.0406029
## TR2-COTR1    0.007176360 -0.054006280 0.068359000 0.9998711
## TR3-COTR1    0.004752486 -0.056430154 0.065935127 0.9999921
## COTR3-COTR2 -0.009610819 -0.070793459 0.051571822 0.9991266
## TR1-COTR2    0.070228835  0.009046195 0.131411475 0.0188071
## TR2-COTR2    0.014268044 -0.046914597 0.075450684 0.9901381
## TR3-COTR2    0.011844170 -0.049338470 0.073026810 0.9967544
## TR1-COTR3    0.079839654  0.018657013 0.141022294 0.0065209
## TR2-COTR3    0.023878862 -0.037303778 0.085061503 0.8657479
## TR3-COTR3    0.021454988 -0.039727652 0.082637629 0.9161273
## TR2-TR1     -0.055960791 -0.117143432 0.005221849 0.0860876
## TR3-TR1     -0.058384665 -0.119567305 0.002797975 0.0670740
## TR3-TR2     -0.002423874 -0.063606514 0.058758766 0.9999999
```

```r
print("For glyphosate in DC")
```

```
## [1] "For glyphosate in DC"
```

```r
Lactate_G_DC <- Lactate[Lactate$Arm == 'glyphosate' & Lactate$Compartment == 'DC',]
res.aov <- aov(Value ~ Week, data = Lactate_G_DC)
summary(res.aov)
```

```
##              Df   Sum Sq   Mean Sq F value Pr(>F)  
## Week          7 0.006629 0.0009469   2.957 0.0343 *
## Residuals    16 0.005123 0.0003202                 
## ---
## Signif. codes:  0 '***' 0.001 '**' 0.01 '*' 0.05 '.' 0.1 ' ' 1
```

```r
TukeyHSD(res.aov, which = "Week")
```

```
##   Tukey multiple comparisons of means
##     95% family-wise confidence level
## 
## Fit: aov(formula = Value ~ Week, data = Lactate_G_DC)
## 
## $Week
##                     diff           lwr         upr     p adj
## C2-C1        0.005990885 -0.0445922709 0.056574040 0.9998625
## COTR1-C1     0.034410541 -0.0161726143 0.084993697 0.3241776
## COTR2-C1     0.010701033 -0.0398821230 0.061284188 0.9944428
## COTR3-C1     0.051144797  0.0005616417 0.101727953 0.0465215
## TR1-C1       0.022640464 -0.0279426918 0.073223620 0.7713047
## TR2-C1       0.003567011 -0.0470161448 0.054150167 0.9999959
## TR3-C1       0.028557256 -0.0220258992 0.079140412 0.5371563
## COTR1-C2     0.028419657 -0.0221634990 0.079002812 0.5426846
## COTR2-C2     0.004710148 -0.0458730078 0.055293304 0.9999727
## COTR3-C2     0.045153913 -0.0054292431 0.095737068 0.0986455
## TR1-C2       0.016649579 -0.0339335766 0.067232735 0.9377810
## TR2-C2      -0.002423874 -0.0530070296 0.048159282 0.9999997
## TR3-C2       0.022566372 -0.0280167840 0.073149527 0.7739820
## COTR2-COTR1 -0.023709509 -0.0742926644 0.026873647 0.7314878
## COTR3-COTR1  0.016734256 -0.0338488998 0.067317412 0.9362412
## TR1-COTR1   -0.011770078 -0.0623532332 0.038813078 0.9902639
## TR2-COTR1   -0.030843531 -0.0814266862 0.019739625 0.4479072
## TR3-COTR1   -0.005853285 -0.0564364407 0.044729871 0.9998822
## COTR3-COTR2  0.040443765 -0.0101393911 0.091026920 0.1716256
## TR1-COTR2    0.011939431 -0.0386437245 0.062522587 0.9894263
## TR2-COTR2   -0.007134022 -0.0577171775 0.043449134 0.9995651
## TR3-COTR2    0.017856224 -0.0327269320 0.068439379 0.9135326
## TR1-COTR3   -0.028504333 -0.0790874892 0.022078822 0.5392811
## TR2-COTR3   -0.047577786 -0.0981609422 0.003005369 0.0731534
## TR3-COTR3   -0.022587541 -0.0731706966 0.027995615 0.7732183
## TR2-TR1     -0.019073453 -0.0696566087 0.031509703 0.8840407
## TR3-TR1      0.005916793 -0.0446663631 0.056499948 0.9998734
## TR3-TR2      0.024990246 -0.0255929101 0.075573401 0.6814578
```

```r
print("For glyphosate in TC")
```

```
## [1] "For glyphosate in TC"
```

```r
Lactate_G_TC <- Lactate[Lactate$Arm == 'glyphosate' & Lactate$Compartment == 'TC',]
res.aov <- aov(Value ~ Week, data = Lactate_G_TC)
summary(res.aov)
```

```
##             Df  Sum Sq  Mean Sq F value Pr(>F)  
## Week         7 0.06260 0.008944   3.027 0.0315 *
## Residuals   16 0.04728 0.002955                 
## ---
```

```
## Signif. codes:  0 '***' 0.001 '**' 0.01 '*' 0.05 '.' 0.1 ' ' 1
```

```r
TukeyHSD(res.aov, which = "Week")
```

```
##   Tukey multiple comparisons of means
##     95% family-wise confidence level
##
## Fit: aov(formula = Value ~ Week, data = Lactate_G_TC)
##
## $Week
##                      diff          lwr        upr     p adj
## C2-C1          0.083364326 -0.070300833 0.23702949 0.5822603
## COTR1-C1      -0.016670748 -0.170335908 0.13699441 0.9999235
## COTR2-C1       0.045259759 -0.108405401 0.19892492 0.9643503
## COTR3-C1       0.053536917 -0.100128242 0.20720208 0.9186042
## TR1-C1         0.158377401  0.004712242 0.31204256 0.0409446
## TR2-C1         0.089312873 -0.064352287 0.24297803 0.5036252
## TR3-C1         0.051155382 -0.102509778 0.20482054 0.9342989
## COTR1-C2      -0.100035075 -0.253700234 0.05363009 0.3727881
## COTR2-C2      -0.038104568 -0.191769727 0.11556059 0.9859894
## COTR3-C2      -0.029827409 -0.183492568 0.12383775 0.9967006
## TR1-C2         0.075013075 -0.078652084 0.22867823 0.6932599
## TR2-C2         0.005948546 -0.147716613 0.15961371 0.9999999
## TR3-C2        -0.032208944 -0.185874104 0.12145622 0.9947425
## COTR2-COTR1    0.061930507 -0.091734653 0.21559567 0.8467867
## COTR3-COTR1    0.070207666 -0.083457494 0.22387283 0.7540480
## TR1-COTR1      0.175048150  0.021382990 0.32871331 0.0199370
## TR2-COTR1      0.105983621 -0.047681539 0.25964878 0.3094423
## TR3-COTR1      0.067826130 -0.085839029 0.22149129 0.7825561
## COTR3-COTR2    0.008277159 -0.145388001 0.16194232 0.9999994
## TR1-COTR2      0.113117643 -0.040547517 0.26678280 0.2436368
## TR2-COTR2      0.044053114 -0.109612046 0.19771827 0.9690466
## TR3-COTR2      0.005895623 -0.147769536 0.15956078 0.9999999
## TR1-COTR3      0.104840484 -0.048824676 0.25850564 0.3210309
## TR2-COTR3      0.035775955 -0.117889204 0.18944111 0.9902323
## TR3-COTR3     -0.002381535 -0.156046695 0.15128362 1.0000000
## TR2-TR1       -0.069064529 -0.222729688 0.08460063 0.7678898
## TR3-TR1       -0.107222019 -0.260887179 0.04644314 0.2972106
## TR3-TR2       -0.038157491 -0.191822650 0.11550767 0.9858792
```

```r
print("For Roundup in AC")
```

```
## [1] "For Roundup in AC"
```

```r
Lactate_R_AC <- Lactate[Lactate$Arm == 'roundup' & Lactate$Compartment == 'AC',]
res.aov <- aov(Value ~ Week, data = Lactate_R_AC)
summary(res.aov)
```

```
##             Df Sum Sq Mean Sq F value   Pr(>F)
## Week         7  4.678  0.6683   8.202 0.000264 ***
## Residuals   16  1.304  0.0815
## ---
## Signif. codes:  0 '***' 0.001 '**' 0.01 '*' 0.05 '.' 0.1 ' ' 1
```

```r
TukeyHSD(res.aov, which = "Week")
```

```
##   Tukey multiple comparisons of means
```

```
##     95% family-wise confidence level
##
## Fit: aov(formula = Value ~ Week, data = Lactate_R_AC)
##
## $Week
##                      diff         lwr       upr     p adj
## C2-C1         0.076251474 -0.73066541 0.8831684 0.9999699
## COTR1-C1      0.736730645 -0.07018624 1.5436475 0.0869676
## COTR2-C1      0.647428357 -0.15948853 1.4543452 0.1688990
## COTR3-C1      0.745081896 -0.06183499 1.5519988 0.0815235
## TR1-C1        0.960467964  0.15355108 1.7673849 0.0141388
## TR2-C1        1.352934426  0.54601754 2.1598513 0.0005547
## TR3-C1        1.124561051  0.31764416 1.9314779 0.0035870
## COTR1-C2      0.660479171 -0.14643772 1.4673961 0.1538384
## COTR2-C2      0.571176883 -0.23574000 1.3780938 0.2823782
## COTR3-C2      0.668830422 -0.13808646 1.4757473 0.1448094
## TR1-C2        0.884216491  0.07729960 1.6911334 0.0266319
## TR2-C2        1.276682952  0.46976607 2.0835998 0.0010252
## TR3-C2        1.048309577  0.24139269 1.8552265 0.0067807
## COTR2-COTR1 -0.089302288 -0.89621918 0.7176146 0.9999126
## COTR3-COTR1  0.008351251 -0.79856564 0.8152681 1.0000000
## TR1-COTR1    0.223737319 -0.58317957 1.0306542 0.9740768
## TR2-COTR1    0.616203781 -0.19071311 1.4231207 0.2099215
## TR3-COTR1    0.387830406 -0.41908648 1.1947473 0.7082157
## COTR3-COTR2  0.097653539 -0.70926335 0.9045704 0.9998413
## TR1-COTR2    0.313039607 -0.49387728 1.1199565 0.8690956
## TR2-COTR2    0.705506069 -0.10141082 1.5124230 0.1103575
## TR3-COTR2    0.477132694 -0.32978419 1.2840496 0.4836503
## TR1-COTR3    0.215386068 -0.59153082 1.0223030 0.9789016
## TR2-COTR3    0.607852530 -0.19906436 1.4147694 0.2221454
## TR3-COTR3    0.379479155 -0.42743773 1.1863960 0.7284708
## TR2-TR1      0.392466461 -0.41445043 1.1993833 0.6968151
## TR3-TR1      0.164093086 -0.64282380 0.9710100 0.9956186
## TR3-TR2     -0.228373375 -1.03529026 0.5785435 0.9710805
```

```r
print("For Roundup in DC")
```

```
## [1] "For Roundup in DC"
```

```r
Lactate_R_DC <- Lactate[Lactate$Arm == 'roundup' & Lactate$Compartment == 'DC',]
res.aov <- aov(Value ~ Week, data = Lactate_R_DC)
summary(res.aov)
```

```
##             Df Sum Sq Mean Sq F value   Pr(>F)
## Week         7 16.827  2.4038   19.67 9.62e-07 ***
## Residuals   16  1.955  0.1222
## ---
## Signif. codes:  0 '***' 0.001 '**' 0.01 '*' 0.05 '.' 0.1 ' ' 1
```

```r
TukeyHSD(res.aov, which = "Week")
```

```
##   Tukey multiple comparisons of means
##     95% family-wise confidence level
##
## Fit: aov(formula = Value ~ Week, data = Lactate_R_DC)
##
```

```
## $Week
##                      diff        lwr       upr     p adj
## C2-C1       -0.002265105 -0.9905029 0.9859727 1.0000000
## COTR1-C1     1.994477744  1.0062399 2.9827156 0.0000665
## COTR2-C1     2.000320444  1.0120826 2.9885583 0.0000642
## COTR3-C1     1.923052849  0.9348150 2.9112907 0.0001028
## TR1-C1       1.393293514  0.4050557 2.3815313 0.0032169
## TR2-C1       1.902613982  0.9143762 2.8908518 0.0001165
## TR3-C1       2.065733285  1.0774955 3.0539711 0.0000434
## COTR1-C2     1.996742849  1.0085050 2.9849807 0.0000656
## COTR2-C2     2.002585549  1.0143477 2.9908234 0.0000633
## COTR3-C2     1.925317954  0.9370801 2.9135558 0.0001013
## TR1-C2       1.395558618  0.4073208 2.3837965 0.0031678
## TR2-C2       1.904879087  0.9166413 2.8931169 0.0001149
## TR3-C2       2.067998390  1.0797606 3.0562362 0.0000428
## COTR2-COTR1  0.005842700 -0.9823951 0.9940805 1.0000000
## COTR3-COTR1 -0.071424895 -1.0596627 0.9168129 0.9999952
## TR1-COTR1   -0.601184230 -1.5894221 0.3870536 0.4506147
## TR2-COTR1   -0.091863762 -1.0801016 0.8963741 0.9999731
## TR3-COTR1    0.071255541 -0.9169823 1.0594934 0.9999952
## COTR3-COTR2 -0.077267595 -1.0655054 0.9109702 0.9999917
## TR1-COTR2   -0.607026931 -1.5952648 0.3812109 0.4393848
## TR2-COTR2   -0.097706462 -1.0859443 0.8905314 0.9999591
## TR3-COTR2    0.065412841 -0.9228250 1.0536507 0.9999974
## TR1-COTR3   -0.529759335 -1.5179972 0.4584785 0.5955142
## TR2-COTR3   -0.020438867 -1.0086767 0.9677990 1.0000000
## TR3-COTR3    0.142680436 -0.8455574 1.1309183 0.9994934
## TR2-TR1      0.509320469 -0.4789174 1.4975583 0.6380480
## TR3-TR1      0.672439772 -0.3157981 1.6606776 0.3239133
## TR3-TR2      0.163119303 -0.8251185 1.1513571 0.9988016
```

```r
print("For Roundup in TC")
```

```
## [1] "For Roundup in TC"
```

```r
Lactate_R_TC <- Lactate[Lactate$Arm == 'roundup' & Lactate$Compartment == 'TC',]
res.aov <- aov(Value ~ Week, data = Lactate_R_TC)
summary(res.aov)
```

```
##             Df Sum Sq Mean Sq F value   Pr(>F)    
## Week         7 12.664  1.8091   29.81 4.99e-08 ***
## Residuals   16  0.971  0.0607                     
## ---
## Signif. codes:  0 '***' 0.001 '**' 0.01 '*' 0.05 '.' 0.1 ' ' 1
```

```r
TukeyHSD(res.aov, which = "Week")
```

```
##   Tukey multiple comparisons of means
##     95% family-wise confidence level
##
## Fit: aov(formula = Value ~ Week, data = Lactate_R_TC)
##
## $Week
##                    diff         lwr       upr     p adj
## C2-C1        0.12504649 -0.57137811 0.8214711 0.9979631
## COTR1-C1     1.74921134  1.05278675 2.4456359 0.0000042
```

```
## COTR2-C1     1.76592443  1.06949983 2.4623490 0.0000037
## COTR3-C1     1.72197716  1.02555256 2.4184018 0.0000051
## TR1-C1       1.32891796  0.63249337 2.0253426 0.0001294
## TR2-C1       1.68468761  0.98826301 2.3811122 0.0000068
## TR3-C1       1.93713037  1.24070577 2.6335550 0.0000011
## COTR1-C2     1.62416485  0.92774026 2.3205895 0.0000110
## COTR2-C2     1.64087794  0.94445334 2.3373025 0.0000096
## COTR3-C2     1.59693067  0.90050608 2.2933553 0.0000136
## TR1-C2       1.20387147  0.50744688 1.9002961 0.0003976
## TR2-C2       1.55964112  0.86321652 2.2560657 0.0000184
## TR3-C2       1.81208388  1.11565928 2.5085085 0.0000026
## COTR2-COTR1  0.01671309 -0.67971151 0.7131377 1.0000000
## COTR3-COTR1 -0.02723418 -0.72365878 0.6691904 0.9999999
## TR1-COTR1   -0.42029338 -1.11671798 0.2761312 0.4598979
## TR2-COTR1   -0.06452373 -0.76094833 0.6319009 0.9999737
## TR3-COTR1    0.18791903 -0.50850557 0.8843436 0.9776175
## COTR3-COTR2 -0.04394727 -0.74037187 0.6524773 0.9999981
## TR1-COTR2   -0.43700647 -1.13343107 0.2594181 0.4147105
## TR2-COTR2   -0.08123682 -0.77766142 0.6151878 0.9998758
## TR3-COTR2    0.17120594 -0.52521866 0.8676305 0.9866587
## TR1-COTR3   -0.39305920 -1.08948380 0.3033654 0.5374907
## TR2-COTR3   -0.03728955 -0.73371415 0.6591350 0.9999994
## TR3-COTR3    0.21515321 -0.48127139 0.9115778 0.9544907
## TR2-TR1      0.35576965 -0.34065495 1.0521942 0.6473253
## TR3-TR1      0.60821241 -0.08821219 1.3046370 0.1110217
## TR3-TR2      0.25244276 -0.44398184 0.9488674 0.9025171
```
