# Supplementary material for: Alterations in infant gut microbiome composition and metabolism after exposure to glyphosate and Roundup and/or a spore-based formulation using the SHIME technology
Source: Gut Microbiome (Camb). 2022 Jul 26;3:e6. doi: 10.1017/gmb.2022.5 (PMC11406414; doi:10.1017/gmb.2022.5)
Supplement: Supplementary file 1 [file gmbsup.zip › S2632289722000056sup001.docx]

**SUPPLEMENTARY FIGURES AND TABLES**


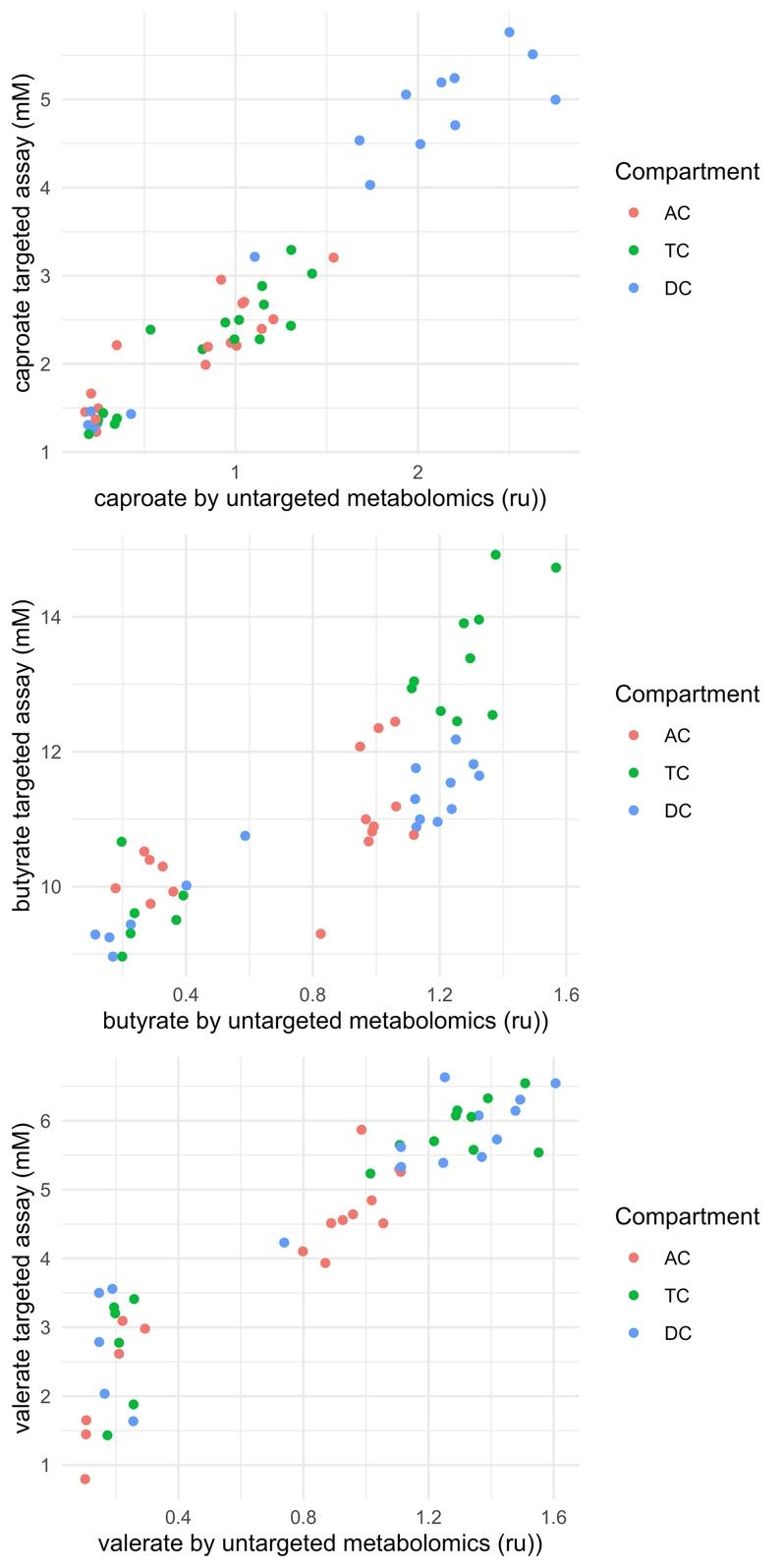


**Supplementary Figure 1. Absolute SCFA concentrations correlate to their relative abundance measured by untargeted metabolomics.** Concentrations of SCFA caproate, butyrate and valerate correlate well with their relative abundance by global untargeted metabolomics in the ascending (AC), transverse (TC) and descending (DC) colon areas of the SHIME system.


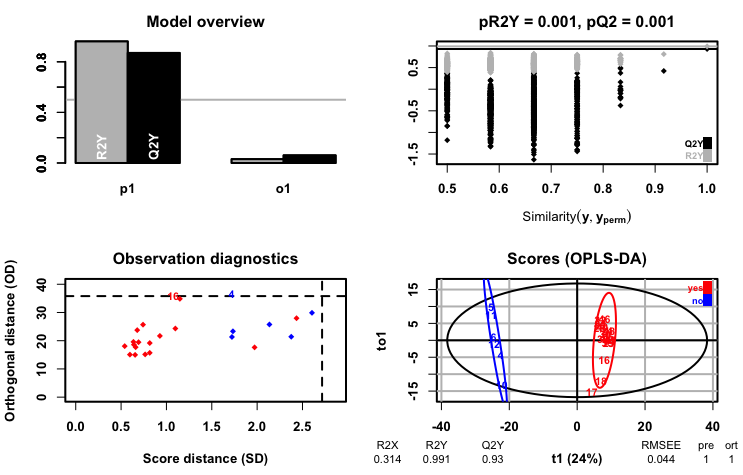


**Supplementary Figure 2. OPLS-DA model separating the samples exposed to Roundup from the rest of the samples**. The figure presents an overview of the predictive ability of the model (top left), the results of the permutation analysis (top right), observation diagnostics (bottom left), and the scores for individual samples showing the clustering by group (bottom right)

**
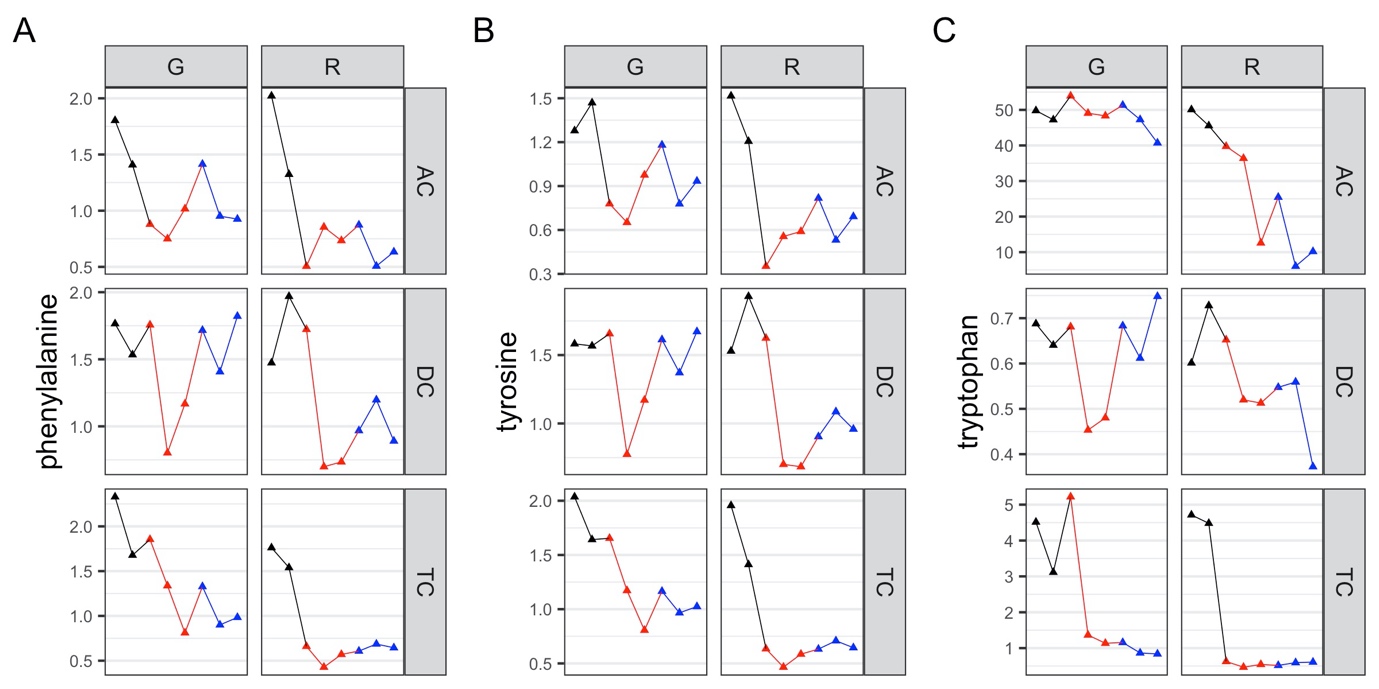
**

**Supplementary Figure 3. Aromatic amino acids, which have their levels decreased in plants following the shikimate pathway inhibition by glyphosate, were relatively unchanged.**


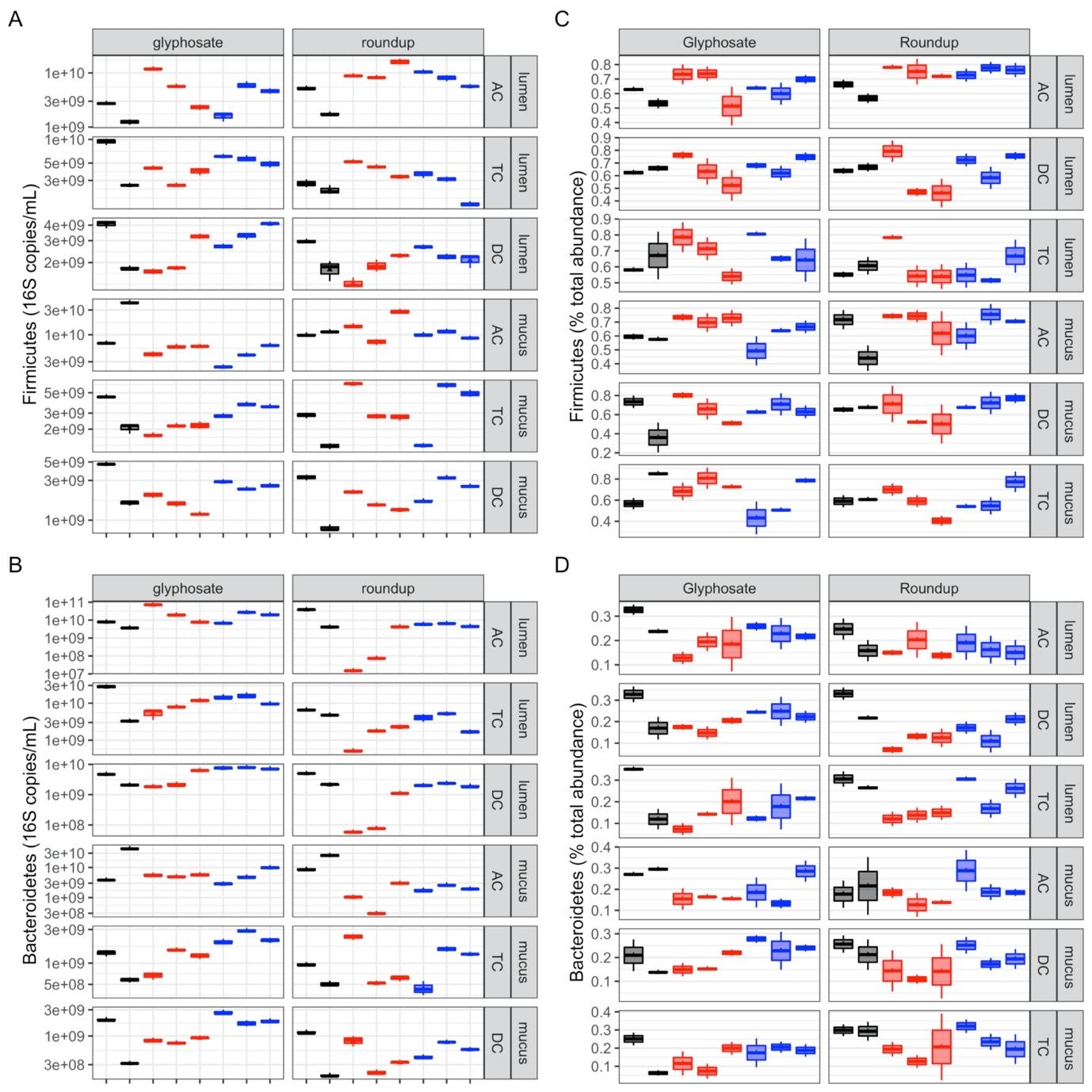


**Supplementary Figure 4. Analysis of the effect of glyphosate and Roundup on Firmicutes and Bacteroidetes levels.** A qPCR analysis was used to measure the number of 16S RNA gene copies per ml for the two major phyla Firmicutes (**A**) and Bacteroidetes (**C**), and the results were compared to those obtained by 16S rRNA gene sequencing for the same taxa of Firmicutes (**B**) and Bacteroidetes (**D**) in the ascending (AC), transverse (TC) and descending (DC) colon compartments. Average weekly levels during control (C1-C2, black), treatment (TR1-TR3, red) and co-treatment (COTR1-COTR3, blue) weeks are shown.

**
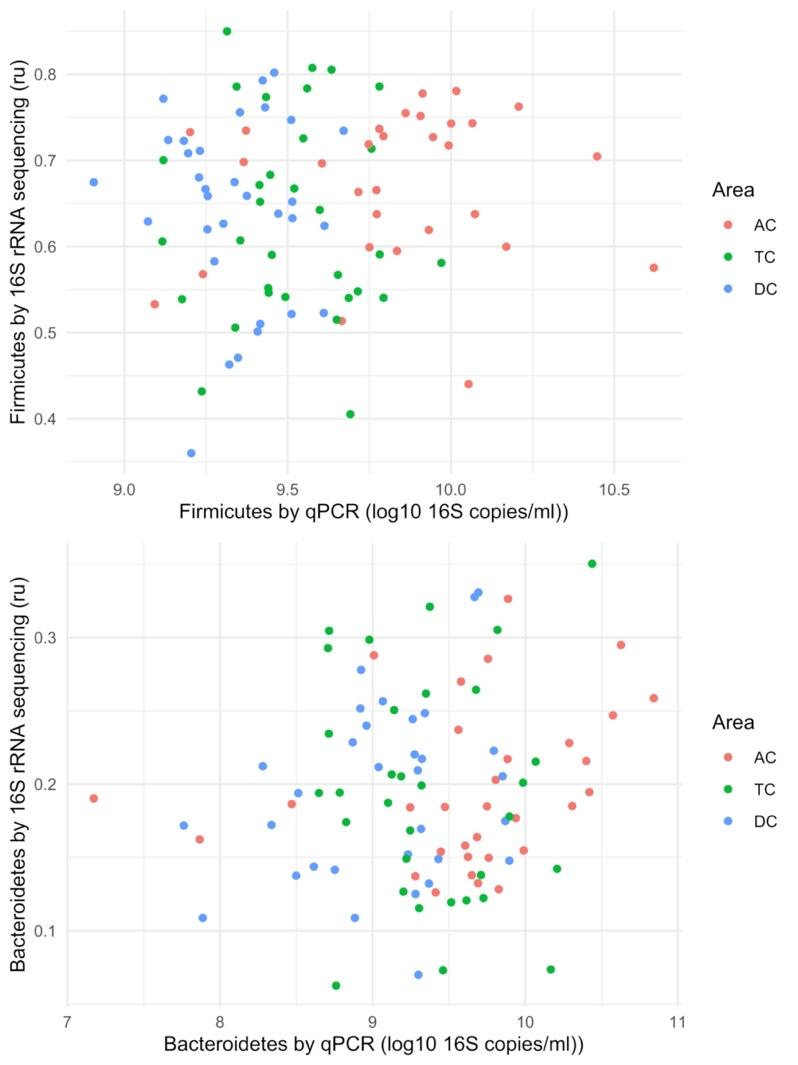
**

**Supplementary Figure 5. Bacteroidetes and Firmicutes levels poorly correlate between the qPCR and the 16S RNA gene sequencing results**.

**
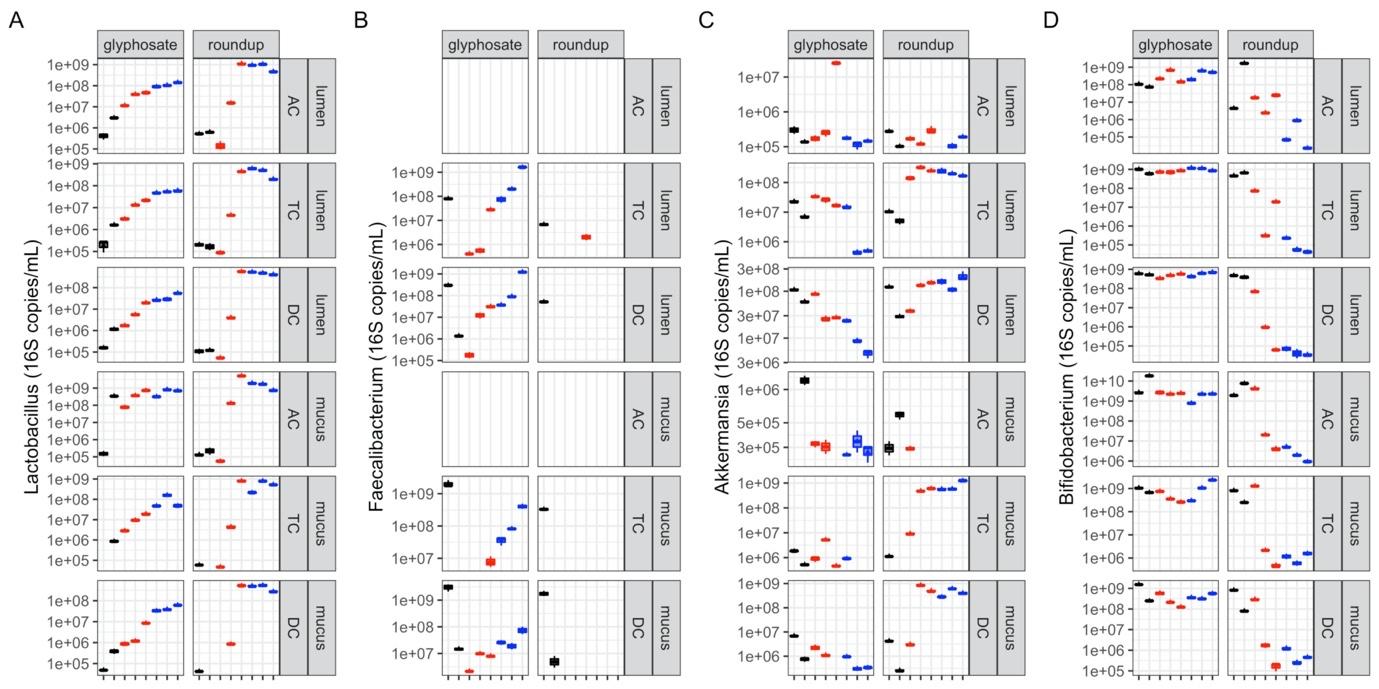
**

**Supplementary Figure 6. Analysis of the effects of glyphosate and Roundup on *Lactobacillus spp*, *Faecalibacterium prausnitzii,* *Akkermansia muciniphila*, *Bifidobacterium spp.,* levels by qPCR.** A qPCR analysis was used to measure the number of 16S gene copies per ml for bacterial species with known roles in health, *Lactobacillus spp*, *Faecalibacterium prausnitzii,* *Akkermansia muciniphila*, *Bifidobacterium spp.*, in the ascending (AC), transverse (TC) and descending (DC) colon compartments. Average weekly levels during control (C1-C2, black), treatment (TR1-TR3, red) and co-treatment (COTR1-COTR3, blue) weeks are reported.


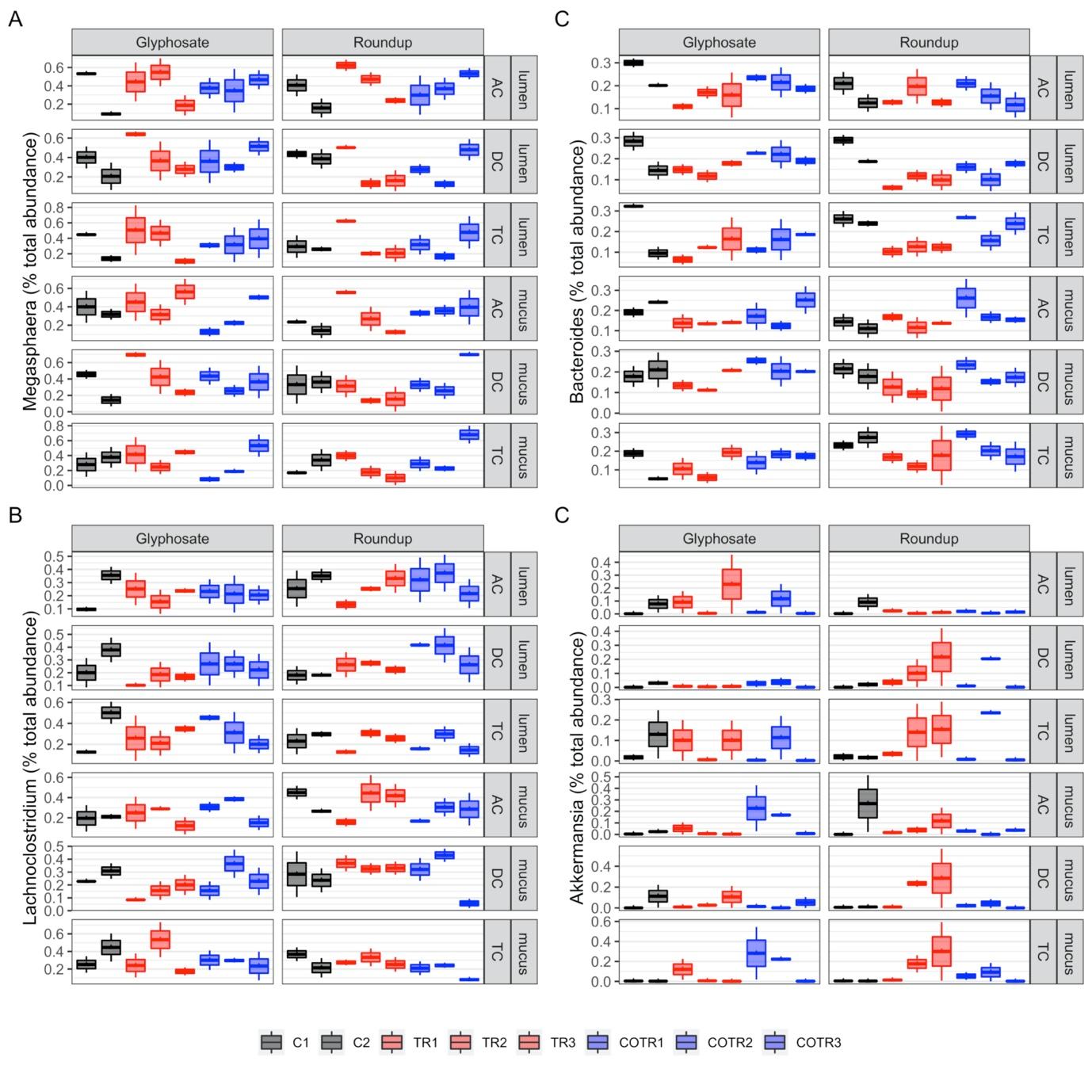


**Supplementary Figure 7. Analysis of the effects of glyphosate and Roundup on the 4 most frequent bacterial genera identified by 16S rRNA gene sequencing.** Effects of the treatments are represented for Megasphaera (**A**), Bacteroides (**B**), Lachnoclostridium (**C**) and Akkermansia (**D**) in the ascending (AC), transverse (TC) and descending (DC) colon simulation chambers. Average weekly levels during control (C1-C2, black), treatment (TR1-TR3, red) and co-treatment (COTR1-COTR3, blue) weeks are reported.

**
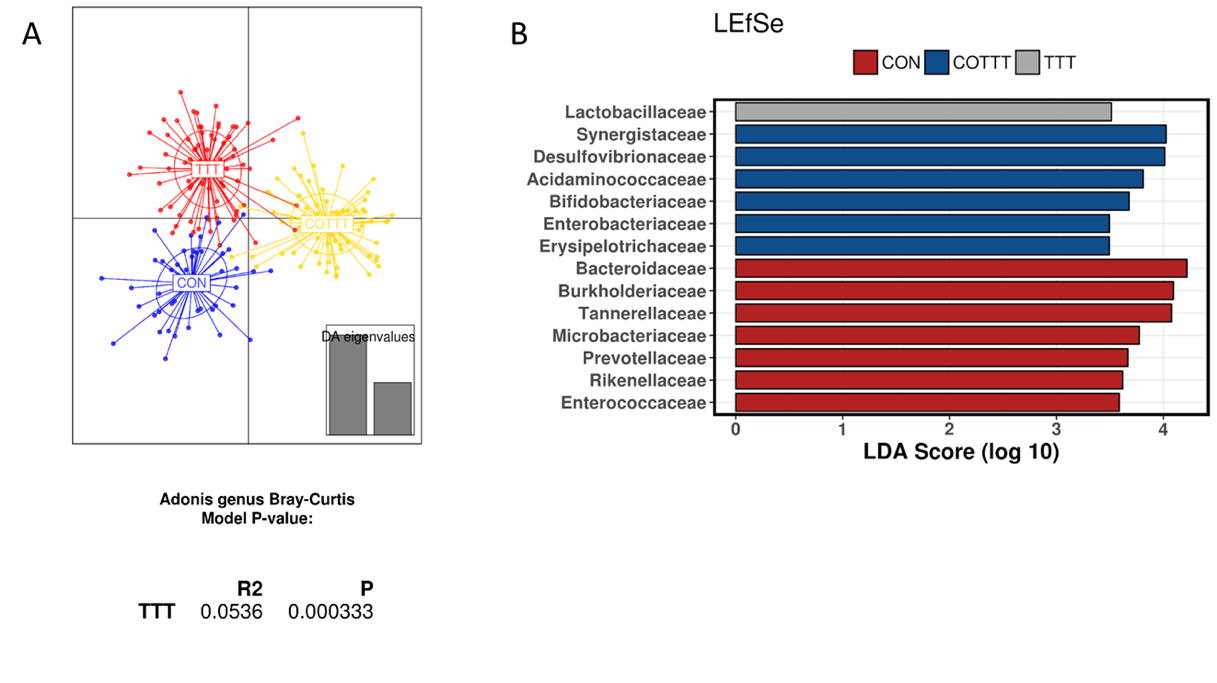
Supplementary Figure S8. Effect of control, treatment and co-treatment conditions on microbial strucutre.** (**A**) Discriminant analysis of principal components and Adonis test based on Bray-Curtis distance at genus level. (**B**) Linear discriminant analysis Effect Size considering control, treatment (glyphosate and Roundup), and co-treatment (glyphosate and Roundup + MegaSporeBiotic probiotic) period.

**
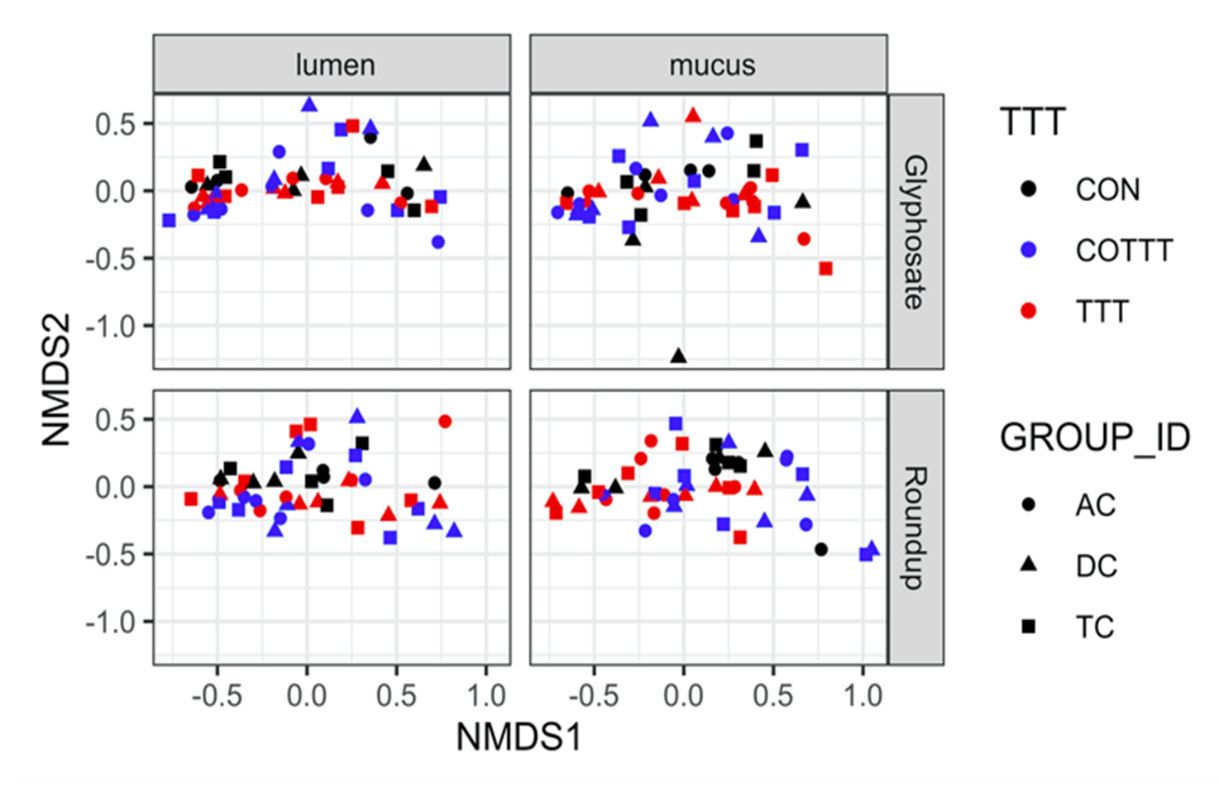
**

**Supplementary Figure S9. Non-metric multi-dimensional scaling including treatment conditions, luminal and mucus compartment and colonic regions. AC = ascending colon, TC = transverse colon, DC = descending colon.**

***Supplementary Table S1****. Primers used to quantify Firmicutes, Bacteroidetes, Bifidobacterium spp., Lactobacillus spp., Enterobacteriaceae,*

*Akkermansia muciniphila and Faecalibacterium prausnitzii by qPCR.*

| **Targeted group** | **Primer name** | **Sequence (5’ – 3’)** | **Reference** |
| --- | --- | --- | --- |
| **Firmicutes (phylum)** | Firm934F | GGAGYATGTGGTTTAATTCGAAGCA | Guo et al. (2008) |
|  | Firm1060R | AGCTGACGACAACCATGCAC |  |
| **Bacteroidetes (phylum)** | Bact934F | GGARCATGTGGTTTAATTCGATGAT | (Guo et al. 2008) |
|  | Bact1060R | AGCTGACGACAACCATGCAG |  |
| ***Enterobacteriaceae*** | Ent-F | GTTGTAAAGCACTTTCAGTGGTGAGGAAGG | (NAKANO et al. 2003) |
|  | ENT-R | GCCTCAAGGGCACAACCTCCAAG |  |
| ***Bifidobacterium* spp.** | Bif243F | TCGCGTCYGGTGTGAAAG | (Rinttilä et al. 2004) |
|  | Bif243R | CCACATCCAGCRTCCAC |  |
| ***Lactobacillus* spp.** | F_lacto_05 | AGCAGTAGGGAATCTTCCA | (Furet et al. 2009) |
|  | R_lacto_04 | CGCCACTGGTGTTCYTCCATATA |  |
| ***Akkermansia muciniphila*** | AM1 | CAGCACGTGAAGGTGGGGAC | (Collado et al. 2007) |
|  | AM2 | CCTTGCGGTTGGCTTCAGAT |  |
| ***Faecalibacterium prausnitzii*** | F. prau-F | CCATGAATTGCCTTCAAAACTGTT | (Sokol et al. 2009) |
|  | F. prau-R | GAGCCTCAGCGTCAGTTGGT |  |

***Supplementary Table S2.*** *qPCR conditions.*

| Program | Cycle | Temperature (°C) | Heating (hh:mm:ss) | Ramp (°C/s) |
| --- | --- | --- | --- | --- |
| Pre-incubation | 1 | 95 | 00:10:00 | 1.6 |
| Amplification | 40 | 95 | 00:00:15 | 1.6 |
|  |  | 60 | 00:00:30 | 1.55 |
|  |  | 72 | 00:00:30 | 1.6 |
| Melting curves | 1 | 95 | 00:00:15 | 1.6 |
|  |  | 60 | 00:01:00 | 1.55 |
|  |  | 75 | 00:00:15 | 0.075 |

***Supplementary Table 3. Effects of a range of glyphosate concentrations on the human gut microbiota during a short-term colonic incubation of 48h****. Measurements assessed change in pH, change in gas pressure, total SCFA, acetate, propionate, butyrate, branched SCFA and lactate production during the 0-48h time interval upon treatment with different concentrations of glyphosate. Enterobacteriaceae concentrations (16S RNA gene copies/mL) after 0h and 48h of incubation upon treatment with different concentrations of glyphosate are also measured.*

| **Glyphosate (mg/L)** | **0** | **0.5** | **1** | **3** | **10** | **100** | **1000** |
| --- | --- | --- | --- | --- | --- | --- | --- |
| pH change | -0.47 | -0.49 | -0.52 | -0.35 | -0.46 | -0.54 | -0.32 |
| Gas (pKa) | 116.6 | 111.2 | 110.9 | 112.4 | 112.4 | 111.5 | 130.1 |
| Total SCFA (mM) | 44.0 | 44.1 | 43.8 | 44.1 | 44.4 | 42.8 | 41.2 |
| Acetate (mM) | 16.5 | 17.1 | 16.7 | 16.7 | 17.1 | 16.2 | 14.3 |
| Propionate (mM) | 13.7 | 13.7 | 13.6 | 13.7 | 13.9 | 13.4 | 12.0 |
| Butyrate (mM) | 12.9 | 12.7 | 12.6 | 12.4 | 12.3 | 12.3 | 14.0 |
| Branched SCFA (mM) | 0.29 | 0.36 | 0.31 | 0.34 | 0.40 | 0.33 | 0.20 |
| Lactate (mM) | 0.65 | 0.71 | 0.79 | 0.54 | 0.65 | 0.38 | 0.56 |
| *Enterobacteriaceae* 0h | 1.3x10^8^ | 8.8x10^7^ | 1.1x10^8^ | 1.0x10^8^ | 1.0x10^8^ | 1.4x10^8^ | 1.5x10^8^ |
| *Enterobacteriaceae* 48h | 2.0x10^10^ | 2.4x10^10^ | 1.9x10^10^ | 2.3x10^10^ | 2.1x10^10^ | 2.2x10^10^ | 1.8x10^10^ |

***Supplementary Table S4.*** *Firmicutes/Bacteroidetes ratio in ascending, transverse and descending colon compartments during the different experimental periods. Values represent the average ± standard deviation (control n = 6; treatment n = 9; co-treatment n = 9). Statistically significant differences are marked in bold.*

|  |  | Firmicutes/Bacteroidetes ratio | | | | | | | | | | | | | | | | | |
| --- | --- | --- | --- | --- | --- | --- | --- | --- | --- | --- | --- | --- | --- | --- | --- | --- | --- | --- | --- |
|  |  | Lumen | | | | | | | | | Mucus | | | | | | | | |
|  |  | Control | | | Treatment | | | Co-treatment | | | Control | | | Treatment | | | Co-treatment | | |
| AC | Glyphosate | 0,35 | ± | 0,03 | **0,25** | **±** | **0,07** | **0,23** | **±** | **0,03** | 1,33 | ± | 0,45 | **0,98** | **±** | **0,22** | **0,77** | **±** | **0,13** |
|  | Roundup | 0,24 | ± | 0,16 | 63,13 | ± | 272,12 | **1,42** | **±** | **0,32** | 0,72 | ± | 0,39 | **14,95** | **±** | **7,05** | **4,87** | **±** | **0,71** |
| TC | Glyphosate | 0,52 | ± | 0,25 | 0,45 | ± | 0,32 | 0,42 | ± | 0,09 | 3,42 | ± | 0,25 | **1,86** | **±** | **0,55** | **1,45** | **±** | **0,21** |
|  | Roundup | 0,45 | ± | 0,07 | 3,36 | ± | 4,07 | **0,79** | **±** | **0,24** | 2,76 | ± | 0,34 | **3,98** | **±** | **1,27** | **3,48** | **±** | **0,52** |
| DC | Glyphosate | 0,87 | ± | 0,04 | **0,69** | **±** | **0,05** | **0,44** | **±** | **0,10** | 3,47 | ± | 1,51 | **1,77** | **±** | **0,19** | **1,27** | **±** | **0,21** |
|  | Roundup | 0,71 | ± | 0,19 | **3,36** | **±** | **4,07** | **1,12** | **±** | **0,21** | 3,44 | ± | 0,86 | 3,98 | ± | 1,27 | **4,30** | **±** | **0,21** |
